# Supplementary material for: Novel insights into neuropathy: The impact of prolonged hyperglycemia on long non-coding RNA expression
Source: PLoS One. 2025 Oct 27;20(10):e0334245. doi: 10.1371/journal.pone.0334245 (PMC12558608; doi:10.1371/journal.pone.0334245)
Supplement: S1 Table — (DOCX) [file pone.0334245.s001.docx]

| Supplementary Table 1. A table containing a complete list of lncRNAs that were differentially expressed in the T1D lumbar spinal cord in relation to control | | | | | | | | | | | |
| --- | --- | --- | --- | --- | --- | --- | --- | --- | --- | --- | --- |
| **gene_id** | **gene_name** | **gene_biotype** | **lncRNA_transcripts** | **transcript_biotype** | **transcript_name** | **baseMean** | **log2FoldChange** | **lfcSE** | **stat** | **pvalue** | **padj** |
| ENSMUSG00000000791 | Il12rb1 | protein_coding | ENSMUST00000212826 | retained_intron | Il12rb1-206 | 112,11195585626 | 2,38682617121467 | 0,34468224607615 | 6,92471456939316 | 4,36855431374071e-12 | 9,02340344203756e-09 |
| ENSMUSG00000002769 | Gnmt | protein_coding | ENSMUST00000233086 | processed_transcript | Gnmt-203 | 53,5226442140214 | 1,08393411573288 | 0,31730231337395 | 3,41609269786645 | 0,000635266179264528 | 0,0125178221138777 |
| ENSMUSG00000002910 | Arrdc2 | protein_coding | ENSMUST00000212855 | processed_transcript | Arrdc2-205 | 1368,88047948948 | 1,75737066596319 | 0,32117301096134 | 5,47172584864152 | 4,45673903711938e-08 | 1,59295642174031e-05 |
| ENSMUSG00000006542 | Prkag3 | protein_coding | ENSMUST00000159728 | nonsense_mediated_decay | Prkag3-203 | 28,8954936391336 | -1,63625062026008 | 0,378980013155979 | -4,31751164562507 | 1,57798085476615e-05 | 0,0012717672726791 |
| ENSMUSG00000008307 | 1700109H08Rik | protein_coding | ENSMUST00000156973 | nonsense_mediated_decay | 1700109H08Rik-204 | 151,944073684356 | 1,07353099496827 | 0,277704066222263 | 3,8657374001469 | 0,000110754056624653 | 0,00435519028819403 |
| ENSMUSG00000008307 | 1700109H08Rik | protein_coding | ENSMUST00000198134 | retained_intron | 1700109H08Rik-205 | 151,944073684356 | 1,07353099496827 | 0,277704066222263 | 3,8657374001469 | 0,000110754056624653 | 0,00435519028819403 |
| ENSMUSG00000012123 | Crybg2 | protein_coding | ENSMUST00000137529 | retained_intron | Crybg2-204 | 32,010230605246 | 1,42812775376128 | 0,38348991741872 | 3,72402946959867 | 0,000196068023915479 | 0,0061939803014259 |
| ENSMUSG00000012123 | Crybg2 | protein_coding | ENSMUST00000149080 | retained_intron | Crybg2-208 | 32,010230605246 | 1,42812775376128 | 0,38348991741872 | 3,72402946959867 | 0,000196068023915479 | 0,0061939803014259 |
| ENSMUSG00000017493 | Igfbp4 | protein_coding | ENSMUST00000177105 | processed_transcript | Igfbp4-206 | 404,698921354841 | -1,18397859638492 | 0,267673274425402 | -4,42322304655367 | 9,72392601233807e-06 | 0,000915806055400415 |
| ENSMUSG00000017697 | Ada | protein_coding | ENSMUST00000156939 | processed_transcript | Ada-202 | 84,5446760063086 | 1,52806201545738 | 0,322500207295926 | 4,73817374652174 | 2,15652878689541e-06 | 0,000341456752700289 |
| ENSMUSG00000019232 | Etnppl | protein_coding | ENSMUST00000199940 | retained_intron | Etnppl-206 | 3691,03096444669 | 1,15203642574389 | 0,19493991518416 | 5,90970004606579 | 3,42731234864535e-09 | 2,4528589016785e-06 |
| ENSMUSG00000019970 | Sgk1 | protein_coding | ENSMUST00000143094 | processed_transcript | Sgk1-211 | 6256,74639161818 | 1,7592345523436 | 0,225426103933625 | 7,80404097682316 | 5,99557107913424e-15 | 3,57575859159566e-11 |
| ENSMUSG00000022096 | Hr | protein_coding | ENSMUST00000159959 | processed_transcript | Hr-202 | 1556,28016406667 | 1,33043657178908 | 0,318391301003941 | 4,17862098491381 | 2,93281969477017e-05 | 0,00189436859129342 |
| ENSMUSG00000022096 | Hr | protein_coding | ENSMUST00000161468 | processed_transcript | Hr-204 | 1556,28016406667 | 1,33043657178908 | 0,318391301003941 | 4,17862098491381 | 2,93281969477017e-05 | 0,00189436859129342 |
| ENSMUSG00000022639 | Dubr | lncRNA | ENSMUST00000243162 | lncRNA | Dubr-208 | 250,773869882011 | -1,3342463474496 | 0,246301436096674 | -5,41712776260833 | 6,05640645344307e-08 | 1,93502186187506e-05 |
| ENSMUSG00000022639 | Dubr | lncRNA | ENSMUST00000191079 | lncRNA | Dubr-205 | 250,773869882011 | -1,3342463474496 | 0,246301436096674 | -5,41712776260833 | 6,05640645344307e-08 | 1,93502186187506e-05 |
| ENSMUSG00000022639 | Dubr | lncRNA | ENSMUST00000243192 | lncRNA | Dubr-209 | 250,773869882011 | -1,3342463474496 | 0,246301436096674 | -5,41712776260833 | 6,05640645344307e-08 | 1,93502186187506e-05 |
| ENSMUSG00000022639 | Dubr | lncRNA | ENSMUST00000243372 | lncRNA | Dubr-211 | 250,773869882011 | -1,3342463474496 | 0,246301436096674 | -5,41712776260833 | 6,05640645344307e-08 | 1,93502186187506e-05 |
| ENSMUSG00000022639 | Dubr | lncRNA | ENSMUST00000186944 | lncRNA | Dubr-204 | 250,773869882011 | -1,3342463474496 | 0,246301436096674 | -5,41712776260833 | 6,05640645344307e-08 | 1,93502186187506e-05 |
| ENSMUSG00000022639 | Dubr | lncRNA | ENSMUST00000186535 | lncRNA | Dubr-202 | 250,773869882011 | -1,3342463474496 | 0,246301436096674 | -5,41712776260833 | 6,05640645344307e-08 | 1,93502186187506e-05 |
| ENSMUSG00000022639 | Dubr | lncRNA | ENSMUST00000241647 | lncRNA | Dubr-206 | 250,773869882011 | -1,3342463474496 | 0,246301436096674 | -5,41712776260833 | 6,05640645344307e-08 | 1,93502186187506e-05 |
| ENSMUSG00000022639 | Dubr | lncRNA | ENSMUST00000242044 | lncRNA | Dubr-207 | 250,773869882011 | -1,3342463474496 | 0,246301436096674 | -5,41712776260833 | 6,05640645344307e-08 | 1,93502186187506e-05 |
| ENSMUSG00000022639 | Dubr | lncRNA | ENSMUST00000246984 | lncRNA | Dubr-212 | 250,773869882011 | -1,3342463474496 | 0,246301436096674 | -5,41712776260833 | 6,05640645344307e-08 | 1,93502186187506e-05 |
| ENSMUSG00000022639 | Dubr | lncRNA | ENSMUST00000186806 | lncRNA | Dubr-203 | 250,773869882011 | -1,3342463474496 | 0,246301436096674 | -5,41712776260833 | 6,05640645344307e-08 | 1,93502186187506e-05 |
| ENSMUSG00000022639 | Dubr | lncRNA | ENSMUST00000243362 | lncRNA | Dubr-210 | 250,773869882011 | -1,3342463474496 | 0,246301436096674 | -5,41712776260833 | 6,05640645344307e-08 | 1,93502186187506e-05 |
| ENSMUSG00000022639 | Dubr | lncRNA | ENSMUST00000169791 | lncRNA | Dubr-201 | 250,773869882011 | -1,3342463474496 | 0,246301436096674 | -5,41712776260833 | 6,05640645344307e-08 | 1,93502186187506e-05 |
| ENSMUSG00000023067 | Cdkn1a | protein_coding | ENSMUST00000232979 | processed_transcript | Cdkn1a-204 | 2259,28236794958 | 2,25283349611184 | 0,512195006726316 | 4,39839019616919 | 1,09056778304411e-05 | 0,000988378662274465 |
| ENSMUSG00000024190 | Dusp1 | protein_coding | ENSMUST00000011196 | processed_transcript | Dusp1-201 | 404,476967742013 | 1,00828384991245 | 0,141916807483515 | 7,1047529027143 | 1,20538003277161e-12 | 3,08095136376423e-09 |
| ENSMUSG00000024222 | Fkbp5 | protein_coding | ENSMUST00000233917 | processed_transcript | Fkbp5-215 | 1226,96776101702 | 1,42215378544029 | 0,226821499997589 | 6,26992496502939 | 3,61222133080019e-10 | 4,23891539925392e-07 |
| ENSMUSG00000024222 | Fkbp5 | protein_coding | ENSMUST00000232690 | processed_transcript | Fkbp5-207 | 1226,96776101702 | 1,42215378544029 | 0,226821499997589 | 6,26992496502939 | 3,61222133080019e-10 | 4,23891539925392e-07 |
| ENSMUSG00000024222 | Fkbp5 | protein_coding | ENSMUST00000147716 | retained_intron | Fkbp5-204 | 1226,96776101702 | 1,42215378544029 | 0,226821499997589 | 6,26992496502939 | 3,61222133080019e-10 | 4,23891539925392e-07 |
| ENSMUSG00000024658 | Gm9750 | lncRNA | ENSMUST00000142025 | lncRNA | Gm9750-201 | 25,2967075119461 | 1,09372996790861 | 0,297002926782257 | 3,68255619484336 | 0,000230906946632407 | 0,00685138820754067 |
| ENSMUSG00000025479 | Cyp2e1 | protein_coding | ENSMUST00000210403 | retained_intron | Cyp2e1-204 | 24,4155676273889 | -2,79063731954252 | 0,591818855639871 | -4,7153572295788 | 2,41287035514251e-06 | 0,000359260636389764 |
| ENSMUSG00000025915 | Sgk3 | protein_coding | ENSMUST00000188830 | processed_transcript | Sgk3-210 | 934,971221850332 | 1,46373199715834 | 0,361679958469441 | 4,04703651082181 | 5,18701531865121e-05 | 0,0026666390365333 |
| ENSMUSG00000025915 | Sgk3 | protein_coding | ENSMUST00000188486 | retained_intron | Sgk3-206 | 934,971221850332 | 1,46373199715834 | 0,361679958469441 | 4,04703651082181 | 5,18701531865121e-05 | 0,0026666390365333 |
| ENSMUSG00000025915 | Sgk3 | protein_coding | ENSMUST00000189050 | retained_intron | Sgk3-211 | 934,971221850332 | 1,46373199715834 | 0,361679958469441 | 4,04703651082181 | 5,18701531865121e-05 | 0,0026666390365333 |
| ENSMUSG00000025915 | Sgk3 | protein_coding | ENSMUST00000188625 | retained_intron | Sgk3-207 | 934,971221850332 | 1,46373199715834 | 0,361679958469441 | 4,04703651082181 | 5,18701531865121e-05 | 0,0026666390365333 |
| ENSMUSG00000025915 | Sgk3 | protein_coding | ENSMUST00000191338 | retained_intron | Sgk3-212 | 934,971221850332 | 1,46373199715834 | 0,361679958469441 | 4,04703651082181 | 5,18701531865121e-05 | 0,0026666390365333 |
| ENSMUSG00000026072 | Il1r1 | protein_coding | ENSMUST00000195402 | processed_transcript | Il1r1-203 | 154,21806100969 | 1,17320142860888 | 0,365817945243592 | 3,20706363332631 | 0,00134097330196922 | 0,021101754018323 |
| ENSMUSG00000026669 | Mcm10 | protein_coding | ENSMUST00000139882 | retained_intron | Mcm10-207 | 95,0630659378715 | 1,24819019273993 | 0,389534357059873 | 3,20431348382469 | 0,00135384978409942 | 0,0211931370206308 |
| ENSMUSG00000026822 | Lcn2 | protein_coding | ENSMUST00000144569 | retained_intron | Lcn2-203 | 219,55384400513 | 2,46262100365066 | 0,839617484333909 | 2,93302730064551 | 0,00335674373862047 | 0,0362789571385527 |
| ENSMUSG00000027360 | Hdc | protein_coding | ENSMUST00000132382 | processed_transcript | Hdc-203 | 61,2122381761554 | 1,21766489019888 | 0,334699400775236 | 3,6380850619347 | 0,000274672715291489 | 0,00771074757949509 |
| ENSMUSG00000027360 | Hdc | protein_coding | ENSMUST00000124396 | processed_transcript | Hdc-202 | 61,2122381761554 | 1,21766489019888 | 0,334699400775236 | 3,6380850619347 | 0,000274672715291489 | 0,00771074757949509 |
| ENSMUSG00000027525 | Phactr3 | protein_coding | ENSMUST00000141272 | retained_intron | Phactr3-207 | 2620,99851429189 | 1,00024540665259 | 0,228042374286296 | 4,38622606777824 | 1,15334252834052e-05 | 0,00103178022585343 |
| ENSMUSG00000028012 | Rrh | protein_coding | ENSMUST00000196281 | retained_intron | Rrh-203 | 17,514187850826 | 2,47793569588731 | 0,526008761750109 | 4,71082589507225 | 2,46714937652646e-06 | 0,000361821611842716 |
| ENSMUSG00000028017 | Egf | protein_coding | ENSMUST00000199272 | retained_intron | Egf-208 | 98,8749610166114 | 1,13708216326383 | 0,262762647097677 | 4,32741173763992 | 1,50871771702961e-05 | 0,00122683720700664 |
| ENSMUSG00000028017 | Egf | protein_coding | ENSMUST00000197250 | retained_intron | Egf-203 | 98,8749610166114 | 1,13708216326383 | 0,262762647097677 | 4,32741173763992 | 1,50871771702961e-05 | 0,00122683720700664 |
| ENSMUSG00000028141 | Oaz3 | protein_coding | ENSMUST00000199318 | retained_intron | Oaz3-203 | 10,4809642915412 | 2,29216689538953 | 0,748997358307141 | 3,06031372469752 | 0,00221105251795897 | 0,0284400802669461 |
| ENSMUSG00000028655 | Mfsd2a | protein_coding | ENSMUST00000138964 | processed_transcript | Mfsd2a-203 | 1222,73739604297 | 1,0171369115501 | 0,169518647885278 | 6,00014761938435 | 1,97138224809102e-09 | 1,67961767537355e-06 |
| ENSMUSG00000028862 | Map3k6 | protein_coding | ENSMUST00000123612 | processed_transcript | Map3k6-202 | 198,418021910477 | 2,35148806640012 | 0,485314536511059 | 4,84528669449104 | 1,26428978997272e-06 | 0,000238112346549389 |
| ENSMUSG00000029368 | Alb | protein_coding | ENSMUST00000201737 | processed_transcript | Alb-203 | 110,43766539561 | -2,93916008520841 | 0,801321460478437 | -3,66789138962228 | 0,000244559020684134 | 0,00713809135086544 |
| ENSMUSG00000029368 | Alb | protein_coding | ENSMUST00000201356 | processed_transcript | Alb-202 | 110,43766539561 | -2,93916008520841 | 0,801321460478437 | -3,66789138962228 | 0,000244559020684134 | 0,00713809135086544 |
| ENSMUSG00000029716 | Tfr2 | protein_coding | ENSMUST00000197705 | retained_intron | Tfr2-205 | 139,739032160862 | 1,42463173150309 | 0,314567469038516 | 4,52885905798688 | 5,93030383727414e-06 | 0,000659037243829248 |
| ENSMUSG00000029716 | Tfr2 | protein_coding | ENSMUST00000200190 | retained_intron | Tfr2-211 | 139,739032160862 | 1,42463173150309 | 0,314567469038516 | 4,52885905798688 | 5,93030383727414e-06 | 0,000659037243829248 |
| ENSMUSG00000029862 | Clcn1 | protein_coding | ENSMUST00000163235 | nonsense_mediated_decay | Clcn1-203 | 112,767551003201 | 1,16708906641438 | 0,334377387455126 | 3,49033490361548 | 0,000482415581090234 | 0,0108298363574234 |
| ENSMUSG00000029862 | Clcn1 | protein_coding | ENSMUST00000169902 | retained_intron | Clcn1-209 | 112,767551003201 | 1,16708906641438 | 0,334377387455126 | 3,49033490361548 | 0,000482415581090234 | 0,0108298363574234 |
| ENSMUSG00000033102 | Cdc14b | protein_coding | ENSMUST00000221788 | retained_intron | Cdc14b-208 | 483,899135637867 | 1,06056036148817 | 0,28278599910012 | 3,75039911757681 | 0,00017655332666037 | 0,0057665014321032 |
| ENSMUSG00000033102 | Cdc14b | protein_coding | ENSMUST00000221217 | nonsense_mediated_decay | Cdc14b-205 | 483,899135637867 | 1,06056036148817 | 0,28278599910012 | 3,75039911757681 | 0,00017655332666037 | 0,0057665014321032 |
| ENSMUSG00000036151 | Tm6sf2 | protein_coding | ENSMUST00000148015 | retained_intron | Tm6sf2-204 | 33,6933773875504 | -1,47465769970433 | 0,444524255286059 | -3,31738410709527 | 0,000908646033825394 | 0,016207552684851 |
| ENSMUSG00000037336 | Mfsd2b | protein_coding | ENSMUST00000150764 | retained_intron | Mfsd2b-207 | 99,6385089322313 | 1,19234198924197 | 0,376122661586807 | 3,17008814148993 | 0,00152392702825786 | 0,0226462644431808 |
| ENSMUSG00000037336 | Mfsd2b | protein_coding | ENSMUST00000147241 | nonsense_mediated_decay | Mfsd2b-206 | 99,6385089322313 | 1,19234198924197 | 0,376122661586807 | 3,17008814148993 | 0,00152392702825786 | 0,0226462644431808 |
| ENSMUSG00000037370 | Enpp1 | protein_coding | ENSMUST00000142974 | retained_intron | Enpp1-204 | 456,575243015339 | -1,47745917616986 | 0,17324422405921 | -8,52818721197252 | 1,48658824653321e-17 | 1,32990184534861e-13 |
| ENSMUSG00000040714 | Klc3 | protein_coding | ENSMUST00000125246 | retained_intron | Klc3-205 | 64,5828208018814 | 1,11167789150017 | 0,298223753277723 | 3,72766380706407 | 0,0001932629461605 | 0,00614184837069923 |
| ENSMUSG00000040728 | Esrp1 | protein_coding | ENSMUST00000127399 | processed_transcript | Esrp1-205 | 18,3170409123284 | -1,94242303736375 | 0,494348560452201 | -3,92925800286935 | 8,52083790362572e-05 | 0,00369878464187535 |
| ENSMUSG00000043747 | 1520401A03Rik | protein_coding | ENSMUST00000062827 | retained_intron | 1520401A03Rik-201 | 19,5502915212875 | 1,89058830375059 | 0,654809603480166 | 2,88723362287684 | 0,00388645537072935 | 0,0394909232125324 |
| ENSMUSG00000043747 | 1520401A03Rik | protein_coding | ENSMUST00000177300 | retained_intron | 1520401A03Rik-203 | 19,5502915212875 | 1,89058830375059 | 0,654809603480166 | 2,88723362287684 | 0,00388645537072935 | 0,0394909232125324 |
| ENSMUSG00000045238 | A730035I17Rik | lncRNA | ENSMUST00000057889 | lncRNA | A730035I17Rik-201 | 10,6337079209701 | 1,55795569998402 | 0,556736159350455 | 2,79837347335529 | 0,00513606875927403 | 0,0466092095944125 |
| ENSMUSG00000045238 | A730035I17Rik | lncRNA | ENSMUST00000205851 | pseudogene | A730035I17Rik-205 | 10,6337079209701 | 1,55795569998402 | 0,556736159350455 | 2,79837347335529 | 0,00513606875927403 | 0,0466092095944125 |
| ENSMUSG00000045238 | A730035I17Rik | lncRNA | ENSMUST00000197382 | lncRNA | A730035I17Rik-202 | 10,6337079209701 | 1,55795569998402 | 0,556736159350455 | 2,79837347335529 | 0,00513606875927403 | 0,0466092095944125 |
| ENSMUSG00000045238 | A730035I17Rik | lncRNA | ENSMUST00000197527 | lncRNA | A730035I17Rik-203 | 10,6337079209701 | 1,55795569998402 | 0,556736159350455 | 2,79837347335529 | 0,00513606875927403 | 0,0466092095944125 |
| ENSMUSG00000045238 | A730035I17Rik | lncRNA | ENSMUST00000198172 | lncRNA | A730035I17Rik-204 | 10,6337079209701 | 1,55795569998402 | 0,556736159350455 | 2,79837347335529 | 0,00513606875927403 | 0,0466092095944125 |
| ENSMUSG00000045467 | Ttll13 | protein_coding | ENSMUST00000206240 | retained_intron | Ttll13-205 | 42,7395953376573 | 1,17852901164167 | 0,300275409641244 | 3,92482692155754 | 8,6792117419114e-05 | 0,00370805866576039 |
| ENSMUSG00000045802 | Hsf3 | protein_coding | ENSMUST00000146965 | retained_intron | Hsf3-203 | 14,2597872907635 | 1,85779016506495 | 0,615755297858963 | 3,01709164586103 | 0,00255212640558061 | 0,0308115017872121 |
| ENSMUSG00000045968 | Teddm2 | protein_coding | ENSMUST00000190620 | retained_intron | Teddm2-203 | 37,200338019974 | 1,40606221109163 | 0,463624088671962 | 3,03276349406101 | 0,00242325437222407 | 0,0298190283547683 |
| ENSMUSG00000046764 | A530053G22Rik | lncRNA | ENSMUST00000203367 | retained_intron | A530053G22Rik-202 | 35,8150160910904 | 2,37463488028336 | 0,441229348374741 | 5,3818606786477 | 7,37198142250128e-08 | 2,18446810225216e-05 |
| ENSMUSG00000046764 | A530053G22Rik | lncRNA | ENSMUST00000204370 | lncRNA | A530053G22Rik-203 | 35,8150160910904 | 2,37463488028336 | 0,441229348374741 | 5,3818606786477 | 7,37198142250128e-08 | 2,18446810225216e-05 |
| ENSMUSG00000046764 | A530053G22Rik | lncRNA | ENSMUST00000205153 | lncRNA | A530053G22Rik-205 | 35,8150160910904 | 2,37463488028336 | 0,441229348374741 | 5,3818606786477 | 7,37198142250128e-08 | 2,18446810225216e-05 |
| ENSMUSG00000046764 | A530053G22Rik | lncRNA | ENSMUST00000204579 | lncRNA | A530053G22Rik-204 | 35,8150160910904 | 2,37463488028336 | 0,441229348374741 | 5,3818606786477 | 7,37198142250128e-08 | 2,18446810225216e-05 |
| ENSMUSG00000046764 | A530053G22Rik | lncRNA | ENSMUST00000060147 | lncRNA | A530053G22Rik-201 | 35,8150160910904 | 2,37463488028336 | 0,441229348374741 | 5,3818606786477 | 7,37198142250128e-08 | 2,18446810225216e-05 |
| ENSMUSG00000047369 | Dnah14 | protein_coding | ENSMUST00000194470 | processed_transcript | Dnah14-206 | 132,121281598783 | 1,50118982184439 | 0,175427833519481 | 8,55730696621576 | 1,15534605014637e-17 | 1,32990184534861e-13 |
| ENSMUSG00000047369 | Dnah14 | protein_coding | ENSMUST00000193365 | processed_transcript | Dnah14-205 | 132,121281598783 | 1,50118982184439 | 0,175427833519481 | 8,55730696621576 | 1,15534605014637e-17 | 1,32990184534861e-13 |
| ENSMUSG00000047798 | Cd300lf | protein_coding | ENSMUST00000146254 | processed_transcript | Cd300lf-206 | 30,8952607743637 | 2,38608403249738 | 0,635293513700025 | 3,75587658466799 | 0,000172735805866963 | 0,00570219379810278 |
| ENSMUSG00000048782 | Insc | protein_coding | ENSMUST00000139670 | retained_intron | Insc-204 | 283,385424761975 | -1,00077086274145 | 0,25897594363919 | -3,86433909141671 | 0,000111390390557175 | 0,00437060716633548 |
| ENSMUSG00000051811 | Cox6b2 | protein_coding | ENSMUST00000183334 | nonsense_mediated_decay | Cox6b2-207 | 46,760238647966 | -1,44247530946474 | 0,267238022541001 | -5,39771734481918 | 6,74940871119799e-08 | 2,1186038712413e-05 |
| ENSMUSG00000051811 | Cox6b2 | protein_coding | ENSMUST00000182272 | retained_intron | Cox6b2-205 | 46,760238647966 | -1,44247530946474 | 0,267238022541001 | -5,39771734481918 | 6,74940871119799e-08 | 2,1186038712413e-05 |
| ENSMUSG00000053113 | Socs3 | protein_coding | ENSMUST00000123255 | processed_transcript | Socs3-202 | 85,3591224983861 | 1,25876280626237 | 0,455680005602572 | 2,76238323118399 | 0,00573810827561851 | 0,049920630066186 |
| ENSMUSG00000053113 | Socs3 | protein_coding | ENSMUST00000132295 | processed_transcript | Socs3-203 | 85,3591224983861 | 1,25876280626237 | 0,455680005602572 | 2,76238323118399 | 0,00573810827561851 | 0,049920630066186 |
| ENSMUSG00000053399 | Adamts18 | protein_coding | ENSMUST00000212527 | processed_transcript | Adamts18-203 | 45,8700784243632 | 1,00632435495362 | 0,292456967530665 | 3,4409313734271 | 0,00057971554594365 | 0,01183557538382 |
| ENSMUSG00000054717 | Hmgb2 | protein_coding | ENSMUST00000211198 | processed_transcript | Hmgb2-203 | 237,337820954459 | 1,29596015563764 | 0,368302351636504 | 3,51873983394134 | 0,000433601695904469 | 0,0102166694077786 |
| ENSMUSG00000056032 | BC018473 | lncRNA | ENSMUST00000132056 | lncRNA | BC018473-201 | 20,2433990081263 | 2,31073004380197 | 0,647325969850268 | 3,56965447305701 | 0,000357452389514883 | 0,00909605408573461 |
| ENSMUSG00000056032 | BC018473 | lncRNA | ENSMUST00000145570 | lncRNA | BC018473-202 | 20,2433990081263 | 2,31073004380197 | 0,647325969850268 | 3,56965447305701 | 0,000357452389514883 | 0,00909605408573461 |
| ENSMUSG00000056032 | BC018473 | lncRNA | ENSMUST00000147508 | lncRNA | BC018473-203 | 20,2433990081263 | 2,31073004380197 | 0,647325969850268 | 3,56965447305701 | 0,000357452389514883 | 0,00909605408573461 |
| ENSMUSG00000056032 | BC018473 | lncRNA | ENSMUST00000156293 | lncRNA | BC018473-204 | 20,2433990081263 | 2,31073004380197 | 0,647325969850268 | 3,56965447305701 | 0,000357452389514883 | 0,00909605408573461 |
| ENSMUSG00000057246 | BC051142 | protein_coding | ENSMUST00000149121 | processed_transcript | BC051142-209 | 116,90419801958 | 1,17307031140815 | 0,191773789323222 | 6,11694807485406 | 9,53844291917806e-10 | 8,53309103549669e-07 |
| ENSMUSG00000057246 | BC051142 | protein_coding | ENSMUST00000131998 | processed_transcript | BC051142-204 | 116,90419801958 | 1,17307031140815 | 0,191773789323222 | 6,11694807485406 | 9,53844291917806e-10 | 8,53309103549669e-07 |
| ENSMUSG00000071350 | Setdb2 | protein_coding | ENSMUST00000160904 | processed_transcript | Setdb2-204 | 292,101635199688 | 1,35562756276361 | 0,441141061933082 | 3,07300244693441 | 0,00211916750056743 | 0,0277463753487745 |
| ENSMUSG00000071537 | Klrg2 | protein_coding | ENSMUST00000202245 | nonsense_mediated_decay | Klrg2-203 | 62,9255454552091 | 1,46857664101283 | 0,368929197800524 | 3,98064628597618 | 6,87281538446537e-05 | 0,00318571017769053 |
| ENSMUSG00000074580 | 4931440P22Rik | transcribed_processed_pseudogene | ENSMUST00000194448 | transcribed_processed_pseudogene | 4931440P22Rik-205 | 45,40887518412 | 1,01374353675704 | 0,365187052935568 | 2,77595694756435 | 0,00550394961345087 | 0,0485585140452973 |
| ENSMUSG00000074580 | 4931440P22Rik | transcribed_processed_pseudogene | ENSMUST00000147424 | processed_transcript | 4931440P22Rik-202 | 45,40887518412 | 1,01374353675704 | 0,365187052935568 | 2,77595694756435 | 0,00550394961345087 | 0,0485585140452973 |
| ENSMUSG00000074580 | 4931440P22Rik | transcribed_processed_pseudogene | ENSMUST00000184377 | processed_transcript | 4931440P22Rik-203 | 45,40887518412 | 1,01374353675704 | 0,365187052935568 | 2,77595694756435 | 0,00550394961345087 | 0,0485585140452973 |
| ENSMUSG00000075555 | Gm10863 | lncRNA | ENSMUST00000244725 | lncRNA | Gm10863-208 | 68,3067015411181 | -1,3252864488116 | 0,329110040815277 | -4,02687941555528 | 5,65220005790225e-05 | 0,00282483696748567 |
| ENSMUSG00000075555 | Gm10863 | lncRNA | ENSMUST00000144651 | lncRNA | Gm10863-203 | 68,3067015411181 | -1,3252864488116 | 0,329110040815277 | -4,02687941555528 | 5,65220005790225e-05 | 0,00282483696748567 |
| ENSMUSG00000075555 | Gm10863 | lncRNA | ENSMUST00000229207 | lncRNA | Gm10863-206 | 68,3067015411181 | -1,3252864488116 | 0,329110040815277 | -4,02687941555528 | 5,65220005790225e-05 | 0,00282483696748567 |
| ENSMUSG00000075555 | Gm10863 | lncRNA | ENSMUST00000130745 | lncRNA | Gm10863-201 | 68,3067015411181 | -1,3252864488116 | 0,329110040815277 | -4,02687941555528 | 5,65220005790225e-05 | 0,00282483696748567 |
| ENSMUSG00000075555 | Gm10863 | lncRNA | ENSMUST00000242492 | lncRNA | Gm10863-207 | 68,3067015411181 | -1,3252864488116 | 0,329110040815277 | -4,02687941555528 | 5,65220005790225e-05 | 0,00282483696748567 |
| ENSMUSG00000075555 | Gm10863 | lncRNA | ENSMUST00000147620 | lncRNA | Gm10863-204 | 68,3067015411181 | -1,3252864488116 | 0,329110040815277 | -4,02687941555528 | 5,65220005790225e-05 | 0,00282483696748567 |
| ENSMUSG00000075555 | Gm10863 | lncRNA | ENSMUST00000246193 | lncRNA | Gm10863-209 | 68,3067015411181 | -1,3252864488116 | 0,329110040815277 | -4,02687941555528 | 5,65220005790225e-05 | 0,00282483696748567 |
| ENSMUSG00000075555 | Gm10863 | lncRNA | ENSMUST00000246786 | lncRNA | Gm10863-210 | 68,3067015411181 | -1,3252864488116 | 0,329110040815277 | -4,02687941555528 | 5,65220005790225e-05 | 0,00282483696748567 |
| ENSMUSG00000075555 | Gm10863 | lncRNA | ENSMUST00000148028 | lncRNA | Gm10863-205 | 68,3067015411181 | -1,3252864488116 | 0,329110040815277 | -4,02687941555528 | 5,65220005790225e-05 | 0,00282483696748567 |
| ENSMUSG00000075555 | Gm10863 | lncRNA | ENSMUST00000131710 | lncRNA | Gm10863-202 | 68,3067015411181 | -1,3252864488116 | 0,329110040815277 | -4,02687941555528 | 5,65220005790225e-05 | 0,00282483696748567 |
| ENSMUSG00000079484 | Phyhd1 | protein_coding | ENSMUST00000147204 | nonsense_mediated_decay | Phyhd1-210 | 804,862285498638 | 1,21370073727995 | 0,214532375003973 | 5,65742460669384 | 1,53661280129807e-08 | 7,11845749621544e-06 |
| ENSMUSG00000079484 | Phyhd1 | protein_coding | ENSMUST00000154988 | retained_intron | Phyhd1-213 | 804,862285498638 | 1,21370073727995 | 0,214532375003973 | 5,65742460669384 | 1,53661280129807e-08 | 7,11845749621544e-06 |
| ENSMUSG00000082361 | Btc | protein_coding | ENSMUST00000200860 | nonsense_mediated_decay | Btc-202 | 29,881094084721 | -1,65373908395613 | 0,433368618576867 | -3,81601023485923 | 0,000135626880902574 | 0,00492218286634657 |
| ENSMUSG00000082361 | Btc | protein_coding | ENSMUST00000201090 | processed_transcript | Btc-203 | 29,881094084721 | -1,65373908395613 | 0,433368618576867 | -3,81601023485923 | 0,000135626880902574 | 0,00492218286634657 |
| ENSMUSG00000083674 | Zfp133-ps | transcribed_unitary_pseudogene | ENSMUST00000119058 | transcribed_unitary_pseudogene | Zfp133-ps-201 | 52,0130151118641 | -1,03746330847504 | 0,345640964302632 | -3,00156351712602 | 0,00268596997844586 | 0,0317001153392832 |
| ENSMUSG00000083674 | Zfp133-ps | transcribed_unitary_pseudogene | ENSMUST00000149523 | processed_transcript | Zfp133-ps-204 | 52,0130151118641 | -1,03746330847504 | 0,345640964302632 | -3,00156351712602 | 0,00268596997844586 | 0,0317001153392832 |
| ENSMUSG00000085156 | Snhg15 | lncRNA | ENSMUST00000246248 | lncRNA | Snhg15-209 | 86,9776183631282 | 1,01993657047094 | 0,268649943561795 | 3,79652627857833 | 0,000146737814229915 | 0,00522674322507177 |
| ENSMUSG00000085156 | Snhg15 | lncRNA | ENSMUST00000129570 | lncRNA | Snhg15-202 | 86,9776183631282 | 1,01993657047094 | 0,268649943561795 | 3,79652627857833 | 0,000146737814229915 | 0,00522674322507177 |
| ENSMUSG00000085156 | Snhg15 | lncRNA | ENSMUST00000245113 | lncRNA | Snhg15-207 | 86,9776183631282 | 1,01993657047094 | 0,268649943561795 | 3,79652627857833 | 0,000146737814229915 | 0,00522674322507177 |
| ENSMUSG00000085156 | Snhg15 | lncRNA | ENSMUST00000245397 | lncRNA | Snhg15-208 | 86,9776183631282 | 1,01993657047094 | 0,268649943561795 | 3,79652627857833 | 0,000146737814229915 | 0,00522674322507177 |
| ENSMUSG00000085156 | Snhg15 | lncRNA | ENSMUST00000241845 | lncRNA | Snhg15-205 | 86,9776183631282 | 1,01993657047094 | 0,268649943561795 | 3,79652627857833 | 0,000146737814229915 | 0,00522674322507177 |
| ENSMUSG00000085156 | Snhg15 | lncRNA | ENSMUST00000147762 | lncRNA | Snhg15-204 | 86,9776183631282 | 1,01993657047094 | 0,268649943561795 | 3,79652627857833 | 0,000146737814229915 | 0,00522674322507177 |
| ENSMUSG00000085156 | Snhg15 | lncRNA | ENSMUST00000134527 | lncRNA | Snhg15-203 | 86,9776183631282 | 1,01993657047094 | 0,268649943561795 | 3,79652627857833 | 0,000146737814229915 | 0,00522674322507177 |
| ENSMUSG00000085156 | Snhg15 | lncRNA | ENSMUST00000124818 | lncRNA | Snhg15-201 | 86,9776183631282 | 1,01993657047094 | 0,268649943561795 | 3,79652627857833 | 0,000146737814229915 | 0,00522674322507177 |
| ENSMUSG00000085156 | Snhg15 | lncRNA | ENSMUST00000242258 | lncRNA | Snhg15-206 | 86,9776183631282 | 1,01993657047094 | 0,268649943561795 | 3,79652627857833 | 0,000146737814229915 | 0,00522674322507177 |
| ENSMUSG00000085287 | 4833418N02Rik | lncRNA | ENSMUST00000246232 | lncRNA | 4833418N02Rik-206 | 39,2608779886552 | 1,14236096770078 | 0,409710037190219 | 2,78821816408273 | 0,00529988397207346 | 0,047389067480429 |
| ENSMUSG00000085287 | 4833418N02Rik | lncRNA | ENSMUST00000160917 | lncRNA | 4833418N02Rik-203 | 39,2608779886552 | 1,14236096770078 | 0,409710037190219 | 2,78821816408273 | 0,00529988397207346 | 0,047389067480429 |
| ENSMUSG00000085287 | 4833418N02Rik | lncRNA | ENSMUST00000241208 | lncRNA | 4833418N02Rik-205 | 39,2608779886552 | 1,14236096770078 | 0,409710037190219 | 2,78821816408273 | 0,00529988397207346 | 0,047389067480429 |
| ENSMUSG00000085287 | 4833418N02Rik | lncRNA | ENSMUST00000146560 | lncRNA | 4833418N02Rik-202 | 39,2608779886552 | 1,14236096770078 | 0,409710037190219 | 2,78821816408273 | 0,00529988397207346 | 0,047389067480429 |
| ENSMUSG00000085287 | 4833418N02Rik | lncRNA | ENSMUST00000137539 | lncRNA | 4833418N02Rik-201 | 39,2608779886552 | 1,14236096770078 | 0,409710037190219 | 2,78821816408273 | 0,00529988397207346 | 0,047389067480429 |
| ENSMUSG00000085287 | 4833418N02Rik | lncRNA | ENSMUST00000161759 | lncRNA | 4833418N02Rik-204 | 39,2608779886552 | 1,14236096770078 | 0,409710037190219 | 2,78821816408273 | 0,00529988397207346 | 0,047389067480429 |
| ENSMUSG00000085511 | Gm11738 | lncRNA | ENSMUST00000134069 | lncRNA | Gm11738-201 | 18,2203460228148 | 2,77477881999758 | 0,74191256687543 | 3,74003480178747 | 0,000183994778164444 | 0,00592092548726299 |
| ENSMUSG00000085601 | Meiosin | protein_coding | ENSMUST00000127433 | nonsense_mediated_decay | Meiosin-201 | 43,2541027968514 | 1,14474981627378 | 0,324280115753511 | 3,53012645753439 | 0,000415361085919072 | 0,0099334243166446 |
| ENSMUSG00000085603 | Gm11346 | transcribed_processed_pseudogene | ENSMUST00000118346 | transcribed_processed_pseudogene | Gm11346-201 | 17,199210478849 | 1,54356433595937 | 0,467066893484178 | 3,3048035677392 | 0,000950429869289832 | 0,0167295176186676 |
| ENSMUSG00000085603 | Gm11346 | transcribed_processed_pseudogene | ENSMUST00000135889 | retained_intron | Gm11346-203 | 17,199210478849 | 1,54356433595937 | 0,467066893484178 | 3,3048035677392 | 0,000950429869289832 | 0,0167295176186676 |
| ENSMUSG00000085603 | Gm11346 | transcribed_processed_pseudogene | ENSMUST00000149751 | processed_transcript | Gm11346-204 | 17,199210478849 | 1,54356433595937 | 0,467066893484178 | 3,3048035677392 | 0,000950429869289832 | 0,0167295176186676 |
| ENSMUSG00000085603 | Gm11346 | transcribed_processed_pseudogene | ENSMUST00000127863 | processed_transcript | Gm11346-202 | 17,199210478849 | 1,54356433595937 | 0,467066893484178 | 3,3048035677392 | 0,000950429869289832 | 0,0167295176186676 |
| ENSMUSG00000086015 | 4833417C18Rik | lncRNA | ENSMUST00000146868 | lncRNA | 4833417C18Rik-201 | 17,5911101443068 | 1,63879131073583 | 0,39532167059083 | 4,14546287909431 | 3,39127939547771e-05 | 0,0020429889206696 |
| ENSMUSG00000086166 | Gm14342 | lncRNA | ENSMUST00000137629 | lncRNA | Gm14342-201 | 16,1441748374127 | 2,33280237952829 | 0,618682526568443 | 3,77059683981591 | 0,000162857589890395 | 0,00556411433790473 |
| ENSMUSG00000086645 | Gm15743 | lncRNA | ENSMUST00000182690 | lncRNA | Gm15743-202 | 45,855360829304 | 1,50433611268473 | 0,420077858725114 | 3,58108879446827 | 0,000342165333791678 | 0,00888537322525501 |
| ENSMUSG00000086645 | Gm15743 | lncRNA | ENSMUST00000129645 | lncRNA | Gm15743-201 | 45,855360829304 | 1,50433611268473 | 0,420077858725114 | 3,58108879446827 | 0,000342165333791678 | 0,00888537322525501 |
| ENSMUSG00000087050 | Dhrs13os | lncRNA | ENSMUST00000150471 | lncRNA | Dhrs13os-201 | 26,7499187750231 | 1,13169214757037 | 0,400297717617072 | 2,82712615577028 | 0,00469678205342945 | 0,0442311766098546 |
| ENSMUSG00000087278 | A930006I01Rik | lncRNA | ENSMUST00000139065 | lncRNA | A930006I01Rik-201 | 31,9885241551933 | -1,75634806537792 | 0,628984887958921 | -2,79235336015359 | 0,00523261788595395 | 0,0469989955901044 |
| ENSMUSG00000087373 | Gm15892 | lncRNA | ENSMUST00000153213 | lncRNA | Gm15892-202 | 38,7916581094283 | 1,29665661883858 | 0,371294902758212 | 3,4922553722289 | 0,000478960092020007 | 0,0108004666430917 |
| ENSMUSG00000087373 | Gm15892 | lncRNA | ENSMUST00000152627 | lncRNA | Gm15892-201 | 38,7916581094283 | 1,29665661883858 | 0,371294902758212 | 3,4922553722289 | 0,000478960092020007 | 0,0108004666430917 |
| ENSMUSG00000087373 | Gm15892 | lncRNA | ENSMUST00000156149 | lncRNA | Gm15892-203 | 38,7916581094283 | 1,29665661883858 | 0,371294902758212 | 3,4922553722289 | 0,000478960092020007 | 0,0108004666430917 |
| ENSMUSG00000087478 | 4930506C21Rik | lncRNA | ENSMUST00000244203 | lncRNA | 4930506C21Rik-205 | 123,833773576364 | -1,50680389539327 | 0,310963832879318 | -4,84559211095797 | 1,26234627374402e-06 | 0,000238112346549389 |
| ENSMUSG00000087478 | 4930506C21Rik | lncRNA | ENSMUST00000244163 | lncRNA | 4930506C21Rik-204 | 123,833773576364 | -1,50680389539327 | 0,310963832879318 | -4,84559211095797 | 1,26234627374402e-06 | 0,000238112346549389 |
| ENSMUSG00000087478 | 4930506C21Rik | lncRNA | ENSMUST00000130639 | lncRNA | 4930506C21Rik-203 | 123,833773576364 | -1,50680389539327 | 0,310963832879318 | -4,84559211095797 | 1,26234627374402e-06 | 0,000238112346549389 |
| ENSMUSG00000087478 | 4930506C21Rik | lncRNA | ENSMUST00000123009 | lncRNA | 4930506C21Rik-201 | 123,833773576364 | -1,50680389539327 | 0,310963832879318 | -4,84559211095797 | 1,26234627374402e-06 | 0,000238112346549389 |
| ENSMUSG00000087543 | Gm16576 | lncRNA | ENSMUST00000128342 | lncRNA | Gm16576-201 | 52,9357678022943 | -1,12692045878241 | 0,269184042670704 | -4,18643114057465 | 2,83374849376296e-05 | 0,00188481145168799 |
| ENSMUSG00000089633 | A230009B12Rik | lncRNA | ENSMUST00000160738 | lncRNA | A230009B12Rik-203 | 43,7381626196551 | -1,29767856211347 | 0,344559567943154 | -3,76619511644954 | 0,000165754273783887 | 0,0055745779446265 |
| ENSMUSG00000089633 | A230009B12Rik | lncRNA | ENSMUST00000159422 | lncRNA | A230009B12Rik-201 | 43,7381626196551 | -1,29767856211347 | 0,344559567943154 | -3,76619511644954 | 0,000165754273783887 | 0,0055745779446265 |
| ENSMUSG00000089633 | A230009B12Rik | lncRNA | ENSMUST00000159602 | lncRNA | A230009B12Rik-202 | 43,7381626196551 | -1,29767856211347 | 0,344559567943154 | -3,76619511644954 | 0,000165754273783887 | 0,0055745779446265 |
| ENSMUSG00000089633 | A230009B12Rik | lncRNA | ENSMUST00000160763 | lncRNA | A230009B12Rik-204 | 43,7381626196551 | -1,29767856211347 | 0,344559567943154 | -3,76619511644954 | 0,000165754273783887 | 0,0055745779446265 |
| ENSMUSG00000089633 | A230009B12Rik | lncRNA | ENSMUST00000247569 | lncRNA | A230009B12Rik-205 | 43,7381626196551 | -1,29767856211347 | 0,344559567943154 | -3,76619511644954 | 0,000165754273783887 | 0,0055745779446265 |
| ENSMUSG00000089736 | Tgfbr3l | protein_coding | ENSMUST00000208029 | processed_transcript | Tgfbr3l-204 | 277,842178330088 | 1,36066915002123 | 0,305432297187174 | 4,45489610153242 | 8,39338318821261e-06 | 0,000825134131887363 |
| ENSMUSG00000089736 | Tgfbr3l | protein_coding | ENSMUST00000209051 | retained_intron | Tgfbr3l-205 | 277,842178330088 | 1,36066915002123 | 0,305432297187174 | 4,45489610153242 | 8,39338318821261e-06 | 0,000825134131887363 |
| ENSMUSG00000089736 | Tgfbr3l | protein_coding | ENSMUST00000142865 | retained_intron | Tgfbr3l-203 | 277,842178330088 | 1,36066915002123 | 0,305432297187174 | 4,45489610153242 | 8,39338318821261e-06 | 0,000825134131887363 |
| ENSMUSG00000090257 | Gm4524 | lncRNA | ENSMUST00000161614 | lncRNA | Gm4524-201 | 31,4548336343884 | -1,73407353850968 | 0,576962790192753 | -3,00552057773146 | 0,0026512665590904 | 0,0314983142597911 |
| ENSMUSG00000092536 | Gm20501 | lncRNA | ENSMUST00000211303 | lncRNA | Gm20501-202 | 89,0907885752652 | 1,14301288393029 | 0,257222767247281 | 4,44366918279615 | 8,84375534445377e-06 | 0,000859959079472646 |
| ENSMUSG00000092536 | Gm20501 | lncRNA | ENSMUST00000173624 | lncRNA | Gm20501-201 | 89,0907885752652 | 1,14301288393029 | 0,257222767247281 | 4,44366918279615 | 8,84375534445377e-06 | 0,000859959079472646 |
| ENSMUSG00000092536 | Gm20501 | lncRNA | ENSMUST00000211692 | lncRNA | Gm20501-203 | 89,0907885752652 | 1,14301288393029 | 0,257222767247281 | 4,44366918279615 | 8,84375534445377e-06 | 0,000859959079472646 |
| ENSMUSG00000093577 | Gm20632 | lncRNA | ENSMUST00000176760 | lncRNA | Gm20632-201 | 100,949613791324 | 1,32767888346638 | 0,401907354655437 | 3,30344510516515 | 0,000955046658436436 | 0,0167555647509101 |
| ENSMUSG00000097237 | Gm26519 | lncRNA | ENSMUST00000181193 | lncRNA | Gm26519-201 | 22,4329291043356 | -1,42471875841379 | 0,505312537744677 | -2,81948032552809 | 0,00481014796522741 | 0,044754023140866 |
| ENSMUSG00000097378 | B230208H11Rik | lncRNA | ENSMUST00000180529 | lncRNA | B230208H11Rik-201 | 13,2491307958218 | 1,7701506375181 | 0,532176609020165 | 3,32624660218959 | 0,000880240144948254 | 0,0159083400741557 |
| ENSMUSG00000097378 | B230208H11Rik | lncRNA | ENSMUST00000181073 | lncRNA | B230208H11Rik-202 | 13,2491307958218 | 1,7701506375181 | 0,532176609020165 | 3,32624660218959 | 0,000880240144948254 | 0,0159083400741557 |
| ENSMUSG00000097476 | Gm26583 | lncRNA | ENSMUST00000181436 | lncRNA | Gm26583-201 | 14,7027065026977 | 2,16763146527952 | 0,612694227005977 | 3,53786827055965 | 0,000403371243398215 | 0,009752862549839 |
| ENSMUSG00000097574 | C920006O11Rik | transcribed_unprocessed_pseudogene | ENSMUST00000238210 | transcribed_unprocessed_pseudogene | C920006O11Rik-202 | 162,288521077346 | 1,00456425993644 | 0,260587992295482 | 3,85499059679371 | 0,000115734077457374 | 0,00448800404299244 |
| ENSMUSG00000097574 | C920006O11Rik | transcribed_unprocessed_pseudogene | ENSMUST00000241112 | processed_transcript | C920006O11Rik-203 | 162,288521077346 | 1,00456425993644 | 0,260587992295482 | 3,85499059679371 | 0,000115734077457374 | 0,00448800404299244 |
| ENSMUSG00000097574 | C920006O11Rik | transcribed_unprocessed_pseudogene | ENSMUST00000243960 | processed_transcript | C920006O11Rik-205 | 162,288521077346 | 1,00456425993644 | 0,260587992295482 | 3,85499059679371 | 0,000115734077457374 | 0,00448800404299244 |
| ENSMUSG00000097574 | C920006O11Rik | transcribed_unprocessed_pseudogene | ENSMUST00000243712 | processed_transcript | C920006O11Rik-204 | 162,288521077346 | 1,00456425993644 | 0,260587992295482 | 3,85499059679371 | 0,000115734077457374 | 0,00448800404299244 |
| ENSMUSG00000097574 | C920006O11Rik | transcribed_unprocessed_pseudogene | ENSMUST00000244552 | processed_transcript | C920006O11Rik-206 | 162,288521077346 | 1,00456425993644 | 0,260587992295482 | 3,85499059679371 | 0,000115734077457374 | 0,00448800404299244 |
| ENSMUSG00000097596 | Gm26673 | lncRNA | ENSMUST00000181126 | lncRNA | Gm26673-201 | 282,720209848273 | -1,14697043175846 | 0,35241098413119 | -3,25463871276919 | 0,00113536699504394 | 0,0189673074466164 |
| ENSMUSG00000097640 | Gm20033 | lncRNA | ENSMUST00000192228 | retained_intron | Gm20033-205 | 85,7526506012741 | 1,07177459793317 | 0,239837082832351 | 4,46876098256394 | 7,86739814527364e-06 | 0,000790806110197955 |
| ENSMUSG00000097640 | Gm20033 | lncRNA | ENSMUST00000180470 | lncRNA | Gm20033-201 | 85,7526506012741 | 1,07177459793317 | 0,239837082832351 | 4,46876098256394 | 7,86739814527364e-06 | 0,000790806110197955 |
| ENSMUSG00000097640 | Gm20033 | lncRNA | ENSMUST00000181929 | retained_intron | Gm20033-204 | 85,7526506012741 | 1,07177459793317 | 0,239837082832351 | 4,46876098256394 | 7,86739814527364e-06 | 0,000790806110197955 |
| ENSMUSG00000097640 | Gm20033 | lncRNA | ENSMUST00000180799 | lncRNA | Gm20033-202 | 85,7526506012741 | 1,07177459793317 | 0,239837082832351 | 4,46876098256394 | 7,86739814527364e-06 | 0,000790806110197955 |
| ENSMUSG00000097640 | Gm20033 | lncRNA | ENSMUST00000180912 | lncRNA | Gm20033-203 | 85,7526506012741 | 1,07177459793317 | 0,239837082832351 | 4,46876098256394 | 7,86739814527364e-06 | 0,000790806110197955 |
| ENSMUSG00000097640 | Gm20033 | lncRNA | ENSMUST00000244117 | lncRNA | Gm20033-207 | 85,7526506012741 | 1,07177459793317 | 0,239837082832351 | 4,46876098256394 | 7,86739814527364e-06 | 0,000790806110197955 |
| ENSMUSG00000097640 | Gm20033 | lncRNA | ENSMUST00000244894 | lncRNA | Gm20033-208 | 85,7526506012741 | 1,07177459793317 | 0,239837082832351 | 4,46876098256394 | 7,86739814527364e-06 | 0,000790806110197955 |
| ENSMUSG00000097736 | 9530059O14Rik | lncRNA | ENSMUST00000181107 | lncRNA | 9530059O14Rik-201 | 34,0249427540447 | 1,57869958288534 | 0,361855746504548 | 4,36278709992934 | 1,28415898272838e-05 | 0,00109410345328458 |
| ENSMUSG00000097736 | 9530059O14Rik | lncRNA | ENSMUST00000181682 | lncRNA | 9530059O14Rik-202 | 34,0249427540447 | 1,57869958288534 | 0,361855746504548 | 4,36278709992934 | 1,28415898272838e-05 | 0,00109410345328458 |
| ENSMUSG00000097736 | 9530059O14Rik | lncRNA | ENSMUST00000215759 | lncRNA | 9530059O14Rik-204 | 34,0249427540447 | 1,57869958288534 | 0,361855746504548 | 4,36278709992934 | 1,28415898272838e-05 | 0,00109410345328458 |
| ENSMUSG00000097736 | 9530059O14Rik | lncRNA | ENSMUST00000181719 | lncRNA | 9530059O14Rik-203 | 34,0249427540447 | 1,57869958288534 | 0,361855746504548 | 4,36278709992934 | 1,28415898272838e-05 | 0,00109410345328458 |
| ENSMUSG00000097848 | Gm807 | lncRNA | ENSMUST00000181742 | lncRNA | Gm807-201 | 13,587049816279 | 1,82726509747931 | 0,5247791634373 | 3,48196960700714 | 0,000497740182382539 | 0,0110217417613718 |
| ENSMUSG00000097848 | Gm807 | lncRNA | ENSMUST00000224929 | lncRNA | Gm807-202 | 13,587049816279 | 1,82726509747931 | 0,5247791634373 | 3,48196960700714 | 0,000497740182382539 | 0,0110217417613718 |
| ENSMUSG00000098120 | Gm5914 | lncRNA | ENSMUST00000182046 | lncRNA | Gm5914-201 | 54,4272234997137 | 1,05118342407136 | 0,361648112324002 | 2,90664706450785 | 0,00365325127411471 | 0,0381576017492472 |
| ENSMUSG00000098120 | Gm5914 | lncRNA | ENSMUST00000182863 | lncRNA | Gm5914-202 | 54,4272234997137 | 1,05118342407136 | 0,361648112324002 | 2,90664706450785 | 0,00365325127411471 | 0,0381576017492472 |
| ENSMUSG00000098708 | Gm27252 | lncRNA | ENSMUST00000246492 | lncRNA | Gm27252-203 | 40,8813188492565 | 1,20583595822439 | 0,330929967937891 | 3,64377987807591 | 0,000268663211539602 | 0,00762707693940825 |
| ENSMUSG00000098708 | Gm27252 | lncRNA | ENSMUST00000184360 | lncRNA | Gm27252-202 | 40,8813188492565 | 1,20583595822439 | 0,330929967937891 | 3,64377987807591 | 0,000268663211539602 | 0,00762707693940825 |
| ENSMUSG00000098708 | Gm27252 | lncRNA | ENSMUST00000183782 | lncRNA | Gm27252-201 | 40,8813188492565 | 1,20583595822439 | 0,330929967937891 | 3,64377987807591 | 0,000268663211539602 | 0,00762707693940825 |
| ENSMUSG00000099170 | 5730403I07Rik | lncRNA | ENSMUST00000183878 | lncRNA | 5730403I07Rik-201 | 13,4942135306025 | 1,53978505460434 | 0,545842192214421 | 2,82093446891236 | 0,00478839833741683 | 0,0446218870068031 |
| ENSMUSG00000099839 | Gm29374 | lncRNA | ENSMUST00000187198 | lncRNA | Gm29374-201 | 32,7937395473774 | 1,18857380111754 | 0,336828112173293 | 3,52872506231321 | 0,000417566709105848 | 0,0099334243166446 |
| ENSMUSG00000100075 | 1700018L02Rik | lncRNA | ENSMUST00000187897 | lncRNA | 1700018L02Rik-201 | 38,4739974267885 | 1,15388725595987 | 0,414365361118052 | 2,78470973743178 | 0,00535756592847457 | 0,0477378334622843 |
| ENSMUSG00000100546 | Gm29483 | lncRNA | ENSMUST00000244657 | lncRNA | Gm29483-204 | 20,0896726052901 | -1,14266657174426 | 0,356759312675843 | -3,20290608021914 | 0,00136048342272203 | 0,0212321585379524 |
| ENSMUSG00000100546 | Gm29483 | lncRNA | ENSMUST00000243412 | lncRNA | Gm29483-203 | 20,0896726052901 | -1,14266657174426 | 0,356759312675843 | -3,20290608021914 | 0,00136048342272203 | 0,0212321585379524 |
| ENSMUSG00000100546 | Gm29483 | lncRNA | ENSMUST00000243065 | lncRNA | Gm29483-202 | 20,0896726052901 | -1,14266657174426 | 0,356759312675843 | -3,20290608021914 | 0,00136048342272203 | 0,0212321585379524 |
| ENSMUSG00000100546 | Gm29483 | lncRNA | ENSMUST00000188188 | lncRNA | Gm29483-201 | 20,0896726052901 | -1,14266657174426 | 0,356759312675843 | -3,20290608021914 | 0,00136048342272203 | 0,0212321585379524 |
| ENSMUSG00000100627 | A830008E24Rik | lncRNA | ENSMUST00000246704 | lncRNA | A830008E24Rik-207 | 18,8168448610741 | 1,1419673543852 | 0,389720666475007 | 2,93022016182565 | 0,0033872192458734 | 0,0364835260259321 |
| ENSMUSG00000100627 | A830008E24Rik | lncRNA | ENSMUST00000246803 | lncRNA | A830008E24Rik-208 | 18,8168448610741 | 1,1419673543852 | 0,389720666475007 | 2,93022016182565 | 0,0033872192458734 | 0,0364835260259321 |
| ENSMUSG00000100627 | A830008E24Rik | lncRNA | ENSMUST00000245448 | lncRNA | A830008E24Rik-205 | 18,8168448610741 | 1,1419673543852 | 0,389720666475007 | 2,93022016182565 | 0,0033872192458734 | 0,0364835260259321 |
| ENSMUSG00000100627 | A830008E24Rik | lncRNA | ENSMUST00000245729 | lncRNA | A830008E24Rik-206 | 18,8168448610741 | 1,1419673543852 | 0,389720666475007 | 2,93022016182565 | 0,0033872192458734 | 0,0364835260259321 |
| ENSMUSG00000100627 | A830008E24Rik | lncRNA | ENSMUST00000244115 | lncRNA | A830008E24Rik-204 | 18,8168448610741 | 1,1419673543852 | 0,389720666475007 | 2,93022016182565 | 0,0033872192458734 | 0,0364835260259321 |
| ENSMUSG00000100627 | A830008E24Rik | lncRNA | ENSMUST00000185465 | lncRNA | A830008E24Rik-201 | 18,8168448610741 | 1,1419673543852 | 0,389720666475007 | 2,93022016182565 | 0,0033872192458734 | 0,0364835260259321 |
| ENSMUSG00000100627 | A830008E24Rik | lncRNA | ENSMUST00000241523 | lncRNA | A830008E24Rik-203 | 18,8168448610741 | 1,1419673543852 | 0,389720666475007 | 2,93022016182565 | 0,0033872192458734 | 0,0364835260259321 |
| ENSMUSG00000100627 | A830008E24Rik | lncRNA | ENSMUST00000190485 | lncRNA | A830008E24Rik-202 | 18,8168448610741 | 1,1419673543852 | 0,389720666475007 | 2,93022016182565 | 0,0033872192458734 | 0,0364835260259321 |
| ENSMUSG00000100671 | Gm28322 | lncRNA | ENSMUST00000185627 | lncRNA | Gm28322-201 | 17,1990013516518 | -1,85022736882488 | 0,602182050663464 | -3,07253822458899 | 0,00212246648140987 | 0,0277463753487745 |
| ENSMUSG00000100747 | 1700084E18Rik | lncRNA | ENSMUST00000187343 | lncRNA | 1700084E18Rik-201 | 20,0417132752739 | 1,45127819392838 | 0,524908786810125 | 2,76481977516096 | 0,00569542532462155 | 0,0496843246748556 |
| ENSMUSG00000100747 | 1700084E18Rik | lncRNA | ENSMUST00000205624 | pseudogene | 1700084E18Rik-202 | 20,0417132752739 | 1,45127819392838 | 0,524908786810125 | 2,76481977516096 | 0,00569542532462155 | 0,0496843246748556 |
| ENSMUSG00000100783 | 2310047D07Rik | lncRNA | ENSMUST00000189493 | lncRNA | 2310047D07Rik-201 | 11,6697879543556 | -1,53773712872128 | 0,46591361220884 | -3,30047693054309 | 0,000965206485679314 | 0,0169142746736281 |
| ENSMUSG00000100783 | 2310047D07Rik | lncRNA | ENSMUST00000242212 | lncRNA | 2310047D07Rik-203 | 11,6697879543556 | -1,53773712872128 | 0,46591361220884 | -3,30047693054309 | 0,000965206485679314 | 0,0169142746736281 |
| ENSMUSG00000100783 | 2310047D07Rik | lncRNA | ENSMUST00000242946 | lncRNA | 2310047D07Rik-204 | 11,6697879543556 | -1,53773712872128 | 0,46591361220884 | -3,30047693054309 | 0,000965206485679314 | 0,0169142746736281 |
| ENSMUSG00000100783 | 2310047D07Rik | lncRNA | ENSMUST00000189639 | lncRNA | 2310047D07Rik-202 | 11,6697879543556 | -1,53773712872128 | 0,46591361220884 | -3,30047693054309 | 0,000965206485679314 | 0,0169142746736281 |
| ENSMUSG00000100783 | 2310047D07Rik | lncRNA | ENSMUST00000245001 | lncRNA | 2310047D07Rik-206 | 11,6697879543556 | -1,53773712872128 | 0,46591361220884 | -3,30047693054309 | 0,000965206485679314 | 0,0169142746736281 |
| ENSMUSG00000100783 | 2310047D07Rik | lncRNA | ENSMUST00000244559 | lncRNA | 2310047D07Rik-205 | 11,6697879543556 | -1,53773712872128 | 0,46591361220884 | -3,30047693054309 | 0,000965206485679314 | 0,0169142746736281 |
| ENSMUSG00000100891 | 2810049E08Rik | lncRNA | ENSMUST00000185600 | lncRNA | 2810049E08Rik-201 | 100,39831002255 | 1,08938291194321 | 0,226972320723678 | 4,79962890836127 | 1,58959893381285e-06 | 0,000276056545377585 |
| ENSMUSG00000103174 | Gm37168 | lncRNA | ENSMUST00000192965 | lncRNA | Gm37168-201 | 39,3676096875702 | -1,7153905581689 | 0,262039858339778 | -6,54629631170311 | 5,89814999875817e-11 | 7,53783569841294e-08 |
| ENSMUSG00000103174 | Gm37168 | lncRNA | ENSMUST00000246071 | lncRNA | Gm37168-202 | 39,3676096875702 | -1,7153905581689 | 0,262039858339778 | -6,54629631170311 | 5,89814999875817e-11 | 7,53783569841294e-08 |
| ENSMUSG00000103502 | 9330121J05Rik | lncRNA | ENSMUST00000192267 | lncRNA | 9330121J05Rik-201 | 22,8601826320245 | 1,12168586032415 | 0,352571387426741 | 3,18144324901357 | 0,0014654323075443 | 0,022127752243551 |
| ENSMUSG00000103804 | Gm37062 | lncRNA | ENSMUST00000195028 | lncRNA | Gm37062-201 | 13,078053834887 | 1,08271753912594 | 0,367274032826786 | 2,94798282032798 | 0,00319854839484275 | 0,0350879386146698 |
| ENSMUSG00000107605 | Gm44117 | lncRNA | ENSMUST00000204442 | lncRNA | Gm44117-201 | 19,5952841782997 | 1,73342689943668 | 0,55666016590235 | 3,11397690299391 | 0,00184583938742238 | 0,0254631906860148 |
| ENSMUSG00000110125 | Gm33148 | lncRNA | ENSMUST00000210835 | lncRNA | Gm33148-201 | 57,6350072027274 | 1,06068985097111 | 0,311485704708706 | 3,40526012891361 | 0,000661010559349261 | 0,0128412605080097 |
| ENSMUSG00000110279 | Gm45552 | lncRNA | ENSMUST00000209340 | lncRNA | Gm45552-201 | 29,6702044800792 | -1,62252310595412 | 0,567272497860911 | -2,86021817040732 | 0,00423349652834614 | 0,0416643123680798 |
| ENSMUSG00000110588 | Gm45774 | lncRNA | ENSMUST00000211992 | lncRNA | Gm45774-201 | 62,6037256069391 | 1,22974518842673 | 0,386675208864123 | 3,18030522835731 | 0,00147120001363829 | 0,0221943588248966 |
| ENSMUSG00000111097 | Gm34069 | lncRNA | ENSMUST00000214060 | lncRNA | Gm34069-201 | 16,3640273186335 | 2,79620226752375 | 0,667615342536044 | 4,18834333090957 | 2,80998192171587e-05 | 0,00187597748296046 |
| ENSMUSG00000111246 | 4930517E14Rik | lncRNA | ENSMUST00000214795 | lncRNA | 4930517E14Rik-201 | 21,0870823499634 | 1,09484128050048 | 0,327894094495281 | 3,33900884121059 | 0,000840778883017451 | 0,0153502201785186 |
| ENSMUSG00000111360 | Gm38642 | lncRNA | ENSMUST00000216718 | lncRNA | Gm38642-203 | 129,120453467319 | 3,29854625457783 | 0,431493793154107 | 7,64448135039514 | 2,0978921250144e-14 | 9,3838714751894e-11 |
| ENSMUSG00000111360 | Gm38642 | lncRNA | ENSMUST00000215107 | lncRNA | Gm38642-202 | 129,120453467319 | 3,29854625457783 | 0,431493793154107 | 7,64448135039514 | 2,0978921250144e-14 | 9,3838714751894e-11 |
| ENSMUSG00000111498 | Gm47059 | lncRNA | ENSMUST00000213406 | lncRNA | Gm47059-201 | 41,3366505274375 | 1,37740827404611 | 0,398831212090591 | 3,45361203509129 | 0,000553132493864851 | 0,0115885791337587 |
| ENSMUSG00000111498 | Gm47059 | lncRNA | ENSMUST00000217104 | lncRNA | Gm47059-202 | 41,3366505274375 | 1,37740827404611 | 0,398831212090591 | 3,45361203509129 | 0,000553132493864851 | 0,0115885791337587 |
| ENSMUSG00000112112 | Gm48508 | lncRNA | ENSMUST00000219735 | lncRNA | Gm48508-201 | 64,1196086231906 | -1,50436230679013 | 0,324420915469114 | -4,63706942141759 | 3,5338374936164e-06 | 0,000468351262487293 |
| ENSMUSG00000112112 | Gm48508 | lncRNA | ENSMUST00000242034 | lncRNA | Gm48508-202 | 64,1196086231906 | -1,50436230679013 | 0,324420915469114 | -4,63706942141759 | 3,5338374936164e-06 | 0,000468351262487293 |
| ENSMUSG00000112352 | Gm40617 | lncRNA | ENSMUST00000218840 | lncRNA | Gm40617-201 | 16,4005262096214 | 1,36986108779123 | 0,496058795020283 | 2,76148936687075 | 0,00575383901620053 | 0,0499261336944034 |
| ENSMUSG00000112830 | Gm47765 | lncRNA | ENSMUST00000220316 | lncRNA | Gm47765-201 | 13,0681097351327 | 1,75675640614687 | 0,537438848783418 | 3,26875589683102 | 0,00108021449412016 | 0,0183022705765132 |
| ENSMUSG00000113622 | Gm49749 | lncRNA | ENSMUST00000223212 | lncRNA | Gm49749-201 | 15,362035447709 | -2,12562019064698 | 0,727555671919864 | -2,92159112035773 | 0,00348248390491715 | 0,0371769701830415 |
| ENSMUSG00000113918 | Gm6566 | lncRNA | ENSMUST00000221468 | lncRNA | Gm6566-201 | 18,7162834879202 | 2,98681850298719 | 0,972194205893891 | 3,07224470674657 | 0,00212455478581607 | 0,0277463753487745 |
| ENSMUSG00000114210 | A330084C13Rik | lncRNA | ENSMUST00000224731 | lncRNA | A330084C13Rik-201 | 52,6264832720869 | 1,00669469086619 | 0,333145286466952 | 3,02178878633499 | 0,00251285817959627 | 0,0304607442746182 |
| ENSMUSG00000114709 | Gm47920 | lncRNA | ENSMUST00000225058 | lncRNA | Gm47920-201 | 19,3901249905555 | 1,38095784269903 | 0,486542454938624 | 2,83830902870178 | 0,00453532495646606 | 0,0435800398072453 |
| ENSMUSG00000115756 | Gm49519 | lncRNA | ENSMUST00000227732 | lncRNA | Gm49519-204 | 84,1621498375741 | -1,04490902533527 | 0,337143866435895 | -3,09929715281934 | 0,00193980358156766 | 0,0262533779738341 |
| ENSMUSG00000115756 | Gm49519 | lncRNA | ENSMUST00000227235 | lncRNA | Gm49519-203 | 84,1621498375741 | -1,04490902533527 | 0,337143866435895 | -3,09929715281934 | 0,00193980358156766 | 0,0262533779738341 |
| ENSMUSG00000115756 | Gm49519 | lncRNA | ENSMUST00000226673 | lncRNA | Gm49519-201 | 84,1621498375741 | -1,04490902533527 | 0,337143866435895 | -3,09929715281934 | 0,00193980358156766 | 0,0262533779738341 |
| ENSMUSG00000115756 | Gm49519 | lncRNA | ENSMUST00000227146 | lncRNA | Gm49519-202 | 84,1621498375741 | -1,04490902533527 | 0,337143866435895 | -3,09929715281934 | 0,00193980358156766 | 0,0262533779738341 |
| ENSMUSG00000116305 | Lncppara | lncRNA | ENSMUST00000230913 | lncRNA | Lncppara-202 | 185,76317482976 | 1,59729316382994 | 0,242789923730906 | 6,57891043946408 | 4,73908102883328e-11 | 6,5224336744527e-08 |
| ENSMUSG00000116305 | Lncppara | lncRNA | ENSMUST00000241017 | lncRNA | Lncppara-203 | 185,76317482976 | 1,59729316382994 | 0,242789923730906 | 6,57891043946408 | 4,73908102883328e-11 | 6,5224336744527e-08 |
| ENSMUSG00000116305 | Lncppara | lncRNA | ENSMUST00000242676 | lncRNA | Lncppara-204 | 185,76317482976 | 1,59729316382994 | 0,242789923730906 | 6,57891043946408 | 4,73908102883328e-11 | 6,5224336744527e-08 |
| ENSMUSG00000116305 | Lncppara | lncRNA | ENSMUST00000245684 | lncRNA | Lncppara-206 | 185,76317482976 | 1,59729316382994 | 0,242789923730906 | 6,57891043946408 | 4,73908102883328e-11 | 6,5224336744527e-08 |
| ENSMUSG00000116305 | Lncppara | lncRNA | ENSMUST00000246157 | lncRNA | Lncppara-209 | 185,76317482976 | 1,59729316382994 | 0,242789923730906 | 6,57891043946408 | 4,73908102883328e-11 | 6,5224336744527e-08 |
| ENSMUSG00000116305 | Lncppara | lncRNA | ENSMUST00000245493 | lncRNA | Lncppara-205 | 185,76317482976 | 1,59729316382994 | 0,242789923730906 | 6,57891043946408 | 4,73908102883328e-11 | 6,5224336744527e-08 |
| ENSMUSG00000116305 | Lncppara | lncRNA | ENSMUST00000245865 | lncRNA | Lncppara-207 | 185,76317482976 | 1,59729316382994 | 0,242789923730906 | 6,57891043946408 | 4,73908102883328e-11 | 6,5224336744527e-08 |
| ENSMUSG00000116305 | Lncppara | lncRNA | ENSMUST00000245866 | lncRNA | Lncppara-208 | 185,76317482976 | 1,59729316382994 | 0,242789923730906 | 6,57891043946408 | 4,73908102883328e-11 | 6,5224336744527e-08 |
| ENSMUSG00000116635 | Gm49699 | transcribed_unprocessed_pseudogene | ENSMUST00000232444 | processed_transcript | Gm49699-202 | 28,4420720913365 | 1,12096429854061 | 0,377733551405176 | 2,9676058543664 | 0,00300128918456774 | 0,0337942517874676 |
| ENSMUSG00000117255 | Gm49864 | lncRNA | ENSMUST00000233925 | lncRNA | Gm49864-201 | 35,3692194841013 | 2,17108764807604 | 0,384369120729897 | 5,64844450551402 | 1,6190609837338e-08 | 7,11845749621544e-06 |
| ENSMUSG00000117628 | Gm50012 | lncRNA | ENSMUST00000234698 | lncRNA | Gm50012-201 | 49,4166282856714 | 1,42276159555844 | 0,312099243862155 | 4,55868325072531 | 5,14753382558025e-06 | 0,000598049839008324 |
| ENSMUSG00000118154 | 9330117O12Rik | lncRNA | ENSMUST00000237954 | lncRNA | 9330117O12Rik-201 | 135,492497580405 | -1,02000493326711 | 0,368539138256458 | -2,7676977215845 | 0,00564537897927704 | 0,0493804736213513 |
| ENSMUSG00000118633 | Gm21104 | transcribed_unprocessed_pseudogene | ENSMUST00000239354 | processed_transcript | Gm21104-203 | 11,7025413432191 | 1,74277177229696 | 0,575293802552649 | 3,02935954561664 | 0,00245072828630285 | 0,0300126149887273 |
| ENSMUSG00000118633 | Gm21104 | transcribed_unprocessed_pseudogene | ENSMUST00000239308 | transcribed_unprocessed_pseudogene | Gm21104-202 | 11,7025413432191 | 1,74277177229696 | 0,575293802552649 | 3,02935954561664 | 0,00245072828630285 | 0,0300126149887273 |
| ENSMUSG00000118633 | Gm21104 | transcribed_unprocessed_pseudogene | ENSMUST00000239230 | processed_transcript | Gm21104-201 | 11,7025413432191 | 1,74277177229696 | 0,575293802552649 | 3,02935954561664 | 0,00245072828630285 | 0,0300126149887273 |
| ENSMUSG00000120016 | NA | lncRNA | ENSMUST00000241040 | lncRNA | NA | 26,4994743135773 | 1,54418478400219 | 0,502815269346469 | 3,07107774592692 | 0,00213287606373815 | 0,0277739581749658 |
| ENSMUSG00000120194 | NA | lncRNA | ENSMUST00000241830 | lncRNA | NA | 33,002595405815 | -1,01999413782229 | 0,302366569740492 | -3,37336941282137 | 0,00074254258571996 | 0,0138535682416074 |
| ENSMUSG00000120226 | NA | lncRNA | ENSMUST00000244508 | lncRNA | NA | 11,9791416093725 | 4,75613164875756 | 1,11420782143933 | 4,26862166755712 | 1,9668453774893e-05 | 0,00149451208231358 |
| ENSMUSG00000120226 | NA | lncRNA | ENSMUST00000247176 | lncRNA | NA | 11,9791416093725 | 4,75613164875756 | 1,11420782143933 | 4,26862166755712 | 1,9668453774893e-05 | 0,00149451208231358 |
| ENSMUSG00000120226 | NA | lncRNA | ENSMUST00000246450 | lncRNA | NA | 11,9791416093725 | 4,75613164875756 | 1,11420782143933 | 4,26862166755712 | 1,9668453774893e-05 | 0,00149451208231358 |
| ENSMUSG00000120226 | NA | lncRNA | ENSMUST00000243317 | lncRNA | NA | 11,9791416093725 | 4,75613164875756 | 1,11420782143933 | 4,26862166755712 | 1,9668453774893e-05 | 0,00149451208231358 |
| ENSMUSG00000120226 | NA | lncRNA | ENSMUST00000241977 | lncRNA | NA | 11,9791416093725 | 4,75613164875756 | 1,11420782143933 | 4,26862166755712 | 1,9668453774893e-05 | 0,00149451208231358 |
| ENSMUSG00000120226 | NA | lncRNA | ENSMUST00000245155 | lncRNA | NA | 11,9791416093725 | 4,75613164875756 | 1,11420782143933 | 4,26862166755712 | 1,9668453774893e-05 | 0,00149451208231358 |
| ENSMUSG00000120226 | NA | lncRNA | ENSMUST00000245924 | lncRNA | NA | 11,9791416093725 | 4,75613164875756 | 1,11420782143933 | 4,26862166755712 | 1,9668453774893e-05 | 0,00149451208231358 |
| ENSMUSG00000120232 | NA | lncRNA | ENSMUST00000242010 | lncRNA | NA | 38,5031705764365 | -1,27971811141861 | 0,326590616814856 | -3,91841665231944 | 8,91325390524356e-05 | 0,00378802705160612 |
| ENSMUSG00000120232 | NA | lncRNA | ENSMUST00000243227 | lncRNA | NA | 38,5031705764365 | -1,27971811141861 | 0,326590616814856 | -3,91841665231944 | 8,91325390524356e-05 | 0,00378802705160612 |
| ENSMUSG00000120232 | NA | lncRNA | ENSMUST00000242867 | lncRNA | NA | 38,5031705764365 | -1,27971811141861 | 0,326590616814856 | -3,91841665231944 | 8,91325390524356e-05 | 0,00378802705160612 |
| ENSMUSG00000120456 | NA | lncRNA | ENSMUST00000243420 | lncRNA | NA | 25,8619747549948 | 1,53064167072464 | 0,484267971992472 | 3,16073281581469 | 0,00157372781798867 | 0,023057821941987 |
| ENSMUSG00000120485 | NA | lncRNA | ENSMUST00000246418 | lncRNA | NA | 17,8939629982765 | 1,75781225452205 | 0,352081026969458 | 4,99263555793517 | 5,95608815276496e-07 | 0,000126864677653894 |
| ENSMUSG00000120485 | NA | lncRNA | ENSMUST00000245894 | lncRNA | NA | 17,8939629982765 | 1,75781225452205 | 0,352081026969458 | 4,99263555793517 | 5,95608815276496e-07 | 0,000126864677653894 |
| ENSMUSG00000120485 | NA | lncRNA | ENSMUST00000243627 | lncRNA | NA | 17,8939629982765 | 1,75781225452205 | 0,352081026969458 | 4,99263555793517 | 5,95608815276496e-07 | 0,000126864677653894 |
| ENSMUSG00000120669 | NA | lncRNA | ENSMUST00000245039 | lncRNA | NA | 17,6204850854677 | 2,18983019794701 | 0,599449827344124 | 3,65306669225196 | 0,000259126893173612 | 0,00741927199801215 |
| ENSMUSG00000121135 | Hmga2-ps1 | lncRNA | ENSMUST00000247245 | lncRNA | Hmga2-ps1-201 | 19,1331271681729 | -1,62087666691258 | 0,450109633990409 | -3,60107081588731 | 0,000316909234592821 | 0,00846289556020111 |
| ENSMUSG00000121236 | NA | lncRNA | ENSMUST00000247546 | lncRNA | NA | 14,4766154412872 | 2,20119978526226 | 0,636655970283382 | 3,45743994874106 | 0,000545333766348815 | 0,0115060280041427 |
