# Supplementary material for: Novel insights into neuropathy: The impact of prolonged hyperglycemia on long non-coding RNA expression
Source: PLoS One. 2025 Oct 27;20(10):e0334245. doi: 10.1371/journal.pone.0334245 (PMC12558608; doi:10.1371/journal.pone.0334245)
Supplement: S2 Table — (DOCX) [file pone.0334245.s002.docx]

| Supplementary Table 2. We found that RNA-RNA interactions are associated with GO-terms involved in biological process (BP), cellular component (CC) and molecular function (MF) | | | | | | | | | | | | | | | | |
| --- | --- | --- | --- | --- | --- | --- | --- | --- | --- | --- | --- | --- | --- | --- | --- | --- |
|  | query | significant | p_value | term_size | query_size | intersection_size | precision | recall | term_id | source | term_name | effective_domain_size | source_order | parents | evidence_codes | intersection |
| 1 | query_1 | TRUE | 0.0499035862713232 | 1 | 5 | 1 | 0.2 | 1 | CORUM:1995 | CORUM | Sgk3 homodimer complex | 1082 | 218 | CORUM:0000000 | CORUM | ENSMUST00000171265 |
| 2 | query_1 | TRUE | 9.96966345387554e-05 | 6865 | 161 | 72 | 0.447204968944099 | 0.0104879825200291 | GO:0032502 | GO:BP | developmental process | 27205 | 8032 | GO:0008150 | IMP IBA,IMP IGI IBA,IMP,IDA IGI IBA,IMP ISO IEA,IMP IGI,IDA IMP,IDA IMP ISO IBA IEA,IMP IBA,IEA,IDA IMP ISO IEA,IDA ISO IEA,IEA,IEA,IEA,IMP,IMP ISO IBA IEA,IMP ISO IEA,ISO IBA,IEA,IDA,IMP,ISS ISO,ISO IEA,IDA IMP IGI ISS ISO IBA IEA,IMP ISO IEA,IDA IMP IBA,ISO IBA IEA,IMP ISS ISO IEA,IMP IGI,IEA,ISO,IMP IEA,IBA,ISS ISO IEA,IDA IMP ISS ISO IEA,IDA IMP IGI ISS ISO IBA IEA,IDA IMP IGI,ISS,IDA IMP IGI ISO IEA,IBA,IDA IMP,ISO IEA,ISO,IDA IMP ISS ISO IBA IEA,IEA,IBA,IMP,IDA IGI ISO IEA,IMP,IBA,IMP IBA,IMP,IMP,IMP,IDA ISO IEA,ISO,IMP,IDA,NAS,IMP ISS ISO IEA,IEA,NAS IEA,IMP ISO,IMP IEA,IMP TAS,IDA IMP IGI ISO IEA,ISO NAS,IEA,IMP,IDA,IMP IEA | ENSMUST00000072631,ENSMUST00000163691,ENSMUST00000229842,ENSMUST00000096232,ENSMUST00000044752,ENSMUST00000173623,ENSMUST00000176059,ENSMUST00000107571,ENSMUST00000052478,ENSMUST00000097921,ENSMUST00000127047,ENSMUST00000196245,ENSMUST00000188970,ENSMUST00000153833,ENSMUST00000151464,ENSMUST00000162069,ENSMUST00000121862,ENSMUST00000210871,ENSMUST00000124203,ENSMUST00000204980,ENSMUST00000033310,ENSMUST00000027629,ENSMUST00000020308,ENSMUST00000108793,ENSMUST00000000095,ENSMUST00000021413,ENSMUST00000138970,ENSMUST00000055458,ENSMUST00000114278,ENSMUST00000108313,ENSMUST00000227683,ENSMUST00000121044,ENSMUST00000108458,ENSMUST00000110993,ENSMUST00000129905,ENSMUST00000051241,ENSMUST00000128241,ENSMUST00000054002,ENSMUST00000161459,ENSMUST00000078508,ENSMUST00000073536,ENSMUST00000106248,ENSMUST00000093113,ENSMUST00000114795,ENSMUST00000028020,ENSMUST00000105520,ENSMUST00000025025,ENSMUST00000023829,ENSMUST00000199615,ENSMUST00000061427,ENSMUST00000167323,ENSMUST00000107348,ENSMUST00000034232,ENSMUST00000040312,ENSMUST00000141380,ENSMUST00000136850,ENSMUST00000050785,ENSMUST00000039164,ENSMUST00000149655,ENSMUST00000105393,ENSMUST00000064265,ENSMUST00000066237,ENSMUST00000235000,ENSMUST00000017841,ENSMUST00000015664,ENSMUST00000057944,ENSMUST00000029846,ENSMUST00000174143,ENSMUST00000035244,ENSMUST00000005607,ENSMUST00000051264,ENSMUST00000029547 |
| 3 | query_1 | TRUE | 0.000154421720948987 | 6228 | 161 | 67 | 0.416149068322981 | 0.0107578676942839 | GO:0048856 | GO:BP | anatomical structure development | 27205 | 13840 | GO:0032502 | IMP,IMP IGI IBA,IMP,IDA IGI,IMP ISO IEA,IMP IGI,IDA IMP ISO IBA IEA,IMP IBA,IEA,IDA IMP ISO IEA,ISO IEA,IEA,IEA,IEA,IMP,IMP ISO IBA IEA,IMP ISO IEA,ISO IBA,IDA,IMP,ISS ISO,ISO IEA,IDA IMP IGI ISS ISO IEA,IMP,IDA IMP,ISO IBA IEA,IMP ISS ISO IEA,IMP IGI,IEA,IMP,IBA,ISS ISO IEA,IMP ISS ISO IEA,IDA IMP IGI ISS ISO IBA IEA,IMP,ISS,IDA IMP IGI ISO IEA,IBA,IDA IMP,ISO IEA,ISO,IDA IMP ISS ISO IEA,IEA,IBA,IMP,IDA IGI ISO IEA,IMP,IBA,IMP IBA,IMP,IMP,IDA ISO IEA,ISO,IMP,IDA,NAS,IMP ISO IEA,IEA,NAS IEA,IMP ISO,IMP IEA,IMP TAS,IDA IMP ISO IEA,ISO NAS,IEA,IMP,IDA | ENSMUST00000072631,ENSMUST00000163691,ENSMUST00000229842,ENSMUST00000096232,ENSMUST00000044752,ENSMUST00000173623,ENSMUST00000107571,ENSMUST00000052478,ENSMUST00000097921,ENSMUST00000127047,ENSMUST00000196245,ENSMUST00000188970,ENSMUST00000153833,ENSMUST00000151464,ENSMUST00000162069,ENSMUST00000121862,ENSMUST00000210871,ENSMUST00000124203,ENSMUST00000033310,ENSMUST00000027629,ENSMUST00000020308,ENSMUST00000108793,ENSMUST00000000095,ENSMUST00000021413,ENSMUST00000138970,ENSMUST00000055458,ENSMUST00000114278,ENSMUST00000108313,ENSMUST00000227683,ENSMUST00000108458,ENSMUST00000110993,ENSMUST00000129905,ENSMUST00000051241,ENSMUST00000128241,ENSMUST00000054002,ENSMUST00000161459,ENSMUST00000078508,ENSMUST00000073536,ENSMUST00000106248,ENSMUST00000093113,ENSMUST00000114795,ENSMUST00000028020,ENSMUST00000105520,ENSMUST00000025025,ENSMUST00000023829,ENSMUST00000199615,ENSMUST00000061427,ENSMUST00000167323,ENSMUST00000107348,ENSMUST00000034232,ENSMUST00000141380,ENSMUST00000136850,ENSMUST00000050785,ENSMUST00000039164,ENSMUST00000149655,ENSMUST00000105393,ENSMUST00000064265,ENSMUST00000066237,ENSMUST00000235000,ENSMUST00000017841,ENSMUST00000015664,ENSMUST00000057944,ENSMUST00000029846,ENSMUST00000174143,ENSMUST00000035244,ENSMUST00000005607,ENSMUST00000051264 |
| 4 | query_1 | TRUE | 0.000314876352868127 | 9152 | 161 | 86 | 0.53416149068323 | 0.00939685314685315 | GO:0032501 | GO:BP | multicellular organismal process | 27205 | 8031 | GO:0008150 | ISS ISO IEA,ISO IEA,IMP,IGI IBA,IDA,IMP ISO IEA,IEA,ISO IEA,IMP IGI,IDA IMP,IDA IMP ISO IBA IEA,IMP IBA,IEA,IDA IMP ISO IEA,IMP ISO IEA,IEA,IEA,IEA,IMP,IMP ISO IBA IEA,IMP ISO IEA,ISO IBA,IEA,IDA,IMP,ISS ISO,ISO IEA,IDA IMP IGI ISS ISO IEA,IMP,IDA IMP,IMP ISS ISO IEA,IMP IGI,IEA,ISO,IMP IEA,ISS ISO IEA,IMP ISS ISO IEA,IDA IMP IGI ISS ISO IBA IEA,ISS ISO IBA IEA,IMP,ISS,IMP ISO IEA,IBA,IMP IBA IEA,IMP,IMP ISO IEA,ISS ISO IEA,IMP ISS ISO IEA,IMP,IDA IMP ISS ISO IBA IEA,IEA,IBA,IMP IGI,IDA ISO IEA,IMP ISO IEA,IMP ISO IEA,IBA,IMP IBA,ISO IBA,IEA,IMP,IMP,IDA IGI ISO IBA IEA,IMP ISO,IMP,IMP,IDA,IMP IBA TAS NAS IEA,IDA IMP ISS ISO IEA,IEA,IMP,IMP ISO IBA,IMP IGI ISO IEA,IGI,NAS IEA,IDA IGI IEA,IMP IGI ISO IBA IEA,IMP ISO IEA,IMP TAS,IDA IMP IGI ISO IEA,NAS,IMP,IMP ISO,IEA,IMP IEA,ISO | ENSMUST00000132462,ENSMUST00000093942,ENSMUST00000072631,ENSMUST00000163691,ENSMUST00000096232,ENSMUST00000044752,ENSMUST00000177197,ENSMUST00000125346,ENSMUST00000173623,ENSMUST00000176059,ENSMUST00000107571,ENSMUST00000052478,ENSMUST00000097921,ENSMUST00000127047,ENSMUST00000196245,ENSMUST00000188970,ENSMUST00000153833,ENSMUST00000151464,ENSMUST00000162069,ENSMUST00000121862,ENSMUST00000210871,ENSMUST00000124203,ENSMUST00000204980,ENSMUST00000033310,ENSMUST00000027629,ENSMUST00000020308,ENSMUST00000108793,ENSMUST00000000095,ENSMUST00000021413,ENSMUST00000138970,ENSMUST00000114278,ENSMUST00000108313,ENSMUST00000227683,ENSMUST00000121044,ENSMUST00000108458,ENSMUST00000129905,ENSMUST00000051241,ENSMUST00000128241,ENSMUST00000079590,ENSMUST00000054002,ENSMUST00000161459,ENSMUST00000078508,ENSMUST00000073536,ENSMUST00000131920,ENSMUST00000106248,ENSMUST00000093113,ENSMUST00000114795,ENSMUST00000238845,ENSMUST00000176249,ENSMUST00000028020,ENSMUST00000105520,ENSMUST00000025025,ENSMUST00000023829,ENSMUST00000199615,ENSMUST00000049346,ENSMUST00000061427,ENSMUST00000167323,ENSMUST00000107348,ENSMUST00000000808,ENSMUST00000029648,ENSMUST00000188073,ENSMUST00000141380,ENSMUST00000136850,ENSMUST00000050785,ENSMUST00000208216,ENSMUST00000039164,ENSMUST00000149655,ENSMUST00000105393,ENSMUST00000064265,ENSMUST00000066237,ENSMUST00000045921,ENSMUST00000091668,ENSMUST00000023295,ENSMUST00000025002,ENSMUST00000235000,ENSMUST00000015234,ENSMUST00000017841,ENSMUST00000015664,ENSMUST00000057944,ENSMUST00000029846,ENSMUST00000174143,ENSMUST00000005607,ENSMUST00000051264,ENSMUST00000058610,ENSMUST00000029547,ENSMUST00000031314 |
| 5 | query_1 | TRUE | 0.00230355379857685 | 4873 | 161 | 54 | 0.335403726708075 | 0.011081469320747 | GO:0007275 | GO:BP | multicellular organism development | 27205 | 2871 | c("GO:0032501", "GO:0048856") | IMP,IGI IBA,IMP ISO IEA,IMP IGI,IDA IMP ISO IBA IEA,IMP,IEA,IDA IMP ISO IEA,ISO IEA,IEA,IEA,IEA,IMP,IMP ISO IBA IEA,IMP ISO IEA,ISO IBA,IDA,IMP,ISS ISO,ISO IEA,IDA IMP IGI ISS ISO IEA,IMP,IDA IMP,ISS ISO IEA,IMP IGI,IEA,ISS ISO IEA,IMP ISS ISO IEA,IDA IMP ISS ISO IBA IEA,IMP,ISS,IBA,ISO IEA,ISO,IDA IMP ISS ISO IEA,IBA,IMP,IDA ISO IEA,IMP,IBA,IMP IBA,IDA ISO IEA,ISO,IMP,IDA,NAS,IMP ISO IEA,IEA,IMP ISO,IMP,IMP TAS,IDA IMP ISO IEA,NAS,IMP | ENSMUST00000072631,ENSMUST00000163691,ENSMUST00000044752,ENSMUST00000173623,ENSMUST00000107571,ENSMUST00000052478,ENSMUST00000097921,ENSMUST00000127047,ENSMUST00000196245,ENSMUST00000188970,ENSMUST00000153833,ENSMUST00000151464,ENSMUST00000162069,ENSMUST00000121862,ENSMUST00000210871,ENSMUST00000124203,ENSMUST00000033310,ENSMUST00000027629,ENSMUST00000020308,ENSMUST00000108793,ENSMUST00000000095,ENSMUST00000021413,ENSMUST00000138970,ENSMUST00000114278,ENSMUST00000108313,ENSMUST00000227683,ENSMUST00000129905,ENSMUST00000051241,ENSMUST00000128241,ENSMUST00000054002,ENSMUST00000161459,ENSMUST00000073536,ENSMUST00000093113,ENSMUST00000114795,ENSMUST00000028020,ENSMUST00000025025,ENSMUST00000023829,ENSMUST00000199615,ENSMUST00000061427,ENSMUST00000167323,ENSMUST00000107348,ENSMUST00000136850,ENSMUST00000050785,ENSMUST00000039164,ENSMUST00000149655,ENSMUST00000105393,ENSMUST00000064265,ENSMUST00000066237,ENSMUST00000017841,ENSMUST00000015664,ENSMUST00000057944,ENSMUST00000029846,ENSMUST00000174143,ENSMUST00000005607 |
| 6 | query_1 | TRUE | 0.00463999527862854 | 4715 | 161 | 52 | 0.322981366459627 | 0.0110286320254507 | GO:0030154 | GO:BP | cell differentiation | 27205 | 7050 | GO:0048869 | IMP IBA,IMP IGI IBA,IMP,IDA IGI IBA,ISO IEA,IDA IMP ISO IBA IEA,IBA,IEA,IMP,IDA,IEA,IEA,IMP ISO IBA IEA,IMP ISO IEA,ISO IBA,IMP,ISS ISO,ISO IEA,IDA IMP IGI IBA,IMP ISO IEA,IDA IMP IBA,IMP,IMP,ISO,IMP IEA,IBA,ISS ISO IEA,IDA IMP ISS ISO IEA,IGI IBA,IDA IGI,IMP ISO IEA,IBA,IDA,ISO,IDA IMP ISS ISO IBA IEA,IEA,IBA,IMP IBA,IMP,IMP,IMP,IDA ISO IEA,IMP,IDA,IMP ISS ISO IEA,NAS IEA,IMP ISO,IEA,IMP,IDA IGI ISO IEA,IDA,IEA | ENSMUST00000072631,ENSMUST00000163691,ENSMUST00000229842,ENSMUST00000096232,ENSMUST00000044752,ENSMUST00000107571,ENSMUST00000052478,ENSMUST00000097921,ENSMUST00000127047,ENSMUST00000196245,ENSMUST00000188970,ENSMUST00000151464,ENSMUST00000121862,ENSMUST00000210871,ENSMUST00000124203,ENSMUST00000027629,ENSMUST00000020308,ENSMUST00000108793,ENSMUST00000000095,ENSMUST00000021413,ENSMUST00000138970,ENSMUST00000114278,ENSMUST00000108313,ENSMUST00000121044,ENSMUST00000108458,ENSMUST00000110993,ENSMUST00000129905,ENSMUST00000051241,ENSMUST00000128241,ENSMUST00000054002,ENSMUST00000078508,ENSMUST00000073536,ENSMUST00000106248,ENSMUST00000114795,ENSMUST00000028020,ENSMUST00000105520,ENSMUST00000167323,ENSMUST00000107348,ENSMUST00000034232,ENSMUST00000040312,ENSMUST00000141380,ENSMUST00000136850,ENSMUST00000039164,ENSMUST00000149655,ENSMUST00000064265,ENSMUST00000235000,ENSMUST00000017841,ENSMUST00000015664,ENSMUST00000057944,ENSMUST00000029846,ENSMUST00000051264,ENSMUST00000029547 |
| 7 | query_1 | TRUE | 0.00572563929490556 | 4748 | 161 | 52 | 0.322981366459627 | 0.0109519797809604 | GO:0048869 | GO:BP | cellular developmental process | 27205 | 13852 | c("GO:0009987", "GO:0032502") | IMP IBA,IMP IGI IBA,IMP,IDA IGI IBA,ISO IEA,IDA IMP ISO IBA IEA,IBA,IEA,IMP,IDA,IEA,IEA,IMP ISO IBA IEA,IMP ISO IEA,ISO IBA,IMP,ISS ISO,ISO IEA,IDA IMP IGI IBA,IMP ISO IEA,IDA IMP IBA,IMP,IMP,ISO,IMP IEA,IBA,ISS ISO IEA,IDA IMP ISS ISO IEA,IGI IBA,IDA IGI,IMP ISO IEA,IBA,IDA,ISO,IDA IMP ISS ISO IBA IEA,IEA,IBA,IMP IBA,IMP,IMP,IMP,IDA ISO IEA,IMP,IDA,IMP ISS ISO IEA,NAS IEA,IMP ISO,IEA,IMP,IDA IGI ISO IEA,IDA,IEA | ENSMUST00000072631,ENSMUST00000163691,ENSMUST00000229842,ENSMUST00000096232,ENSMUST00000044752,ENSMUST00000107571,ENSMUST00000052478,ENSMUST00000097921,ENSMUST00000127047,ENSMUST00000196245,ENSMUST00000188970,ENSMUST00000151464,ENSMUST00000121862,ENSMUST00000210871,ENSMUST00000124203,ENSMUST00000027629,ENSMUST00000020308,ENSMUST00000108793,ENSMUST00000000095,ENSMUST00000021413,ENSMUST00000138970,ENSMUST00000114278,ENSMUST00000108313,ENSMUST00000121044,ENSMUST00000108458,ENSMUST00000110993,ENSMUST00000129905,ENSMUST00000051241,ENSMUST00000128241,ENSMUST00000054002,ENSMUST00000078508,ENSMUST00000073536,ENSMUST00000106248,ENSMUST00000114795,ENSMUST00000028020,ENSMUST00000105520,ENSMUST00000167323,ENSMUST00000107348,ENSMUST00000034232,ENSMUST00000040312,ENSMUST00000141380,ENSMUST00000136850,ENSMUST00000039164,ENSMUST00000149655,ENSMUST00000064265,ENSMUST00000235000,ENSMUST00000017841,ENSMUST00000015664,ENSMUST00000057944,ENSMUST00000029846,ENSMUST00000051264,ENSMUST00000029547 |
| 8 | query_1 | TRUE | 0.0205633512919337 | 4156 | 161 | 46 | 0.285714285714286 | 0.0110683349374398 | GO:0006950 | GO:BP | response to stress | 27205 | 2640 | GO:0050896 | ISS ISO IEA,IMP,IDA IBA,IEA,IEA,IMP ISS ISO IEA,IMP IBA,IGI IBA,IDA,IEA,IMP ISO IBA IEA,IDA ISS ISO IEA,IEA,IDA ISO IEA,ISS IEA,IDA,IMP ISS ISO,IDA IMP,IDA IMP ISS ISO IEA,IGI,IEA,IMP IEA,IMP ISO IEA,IMP ISO IBA IEA,ISO IBA IEA,IDA IMP ISS ISO IBA IEA,IMP,ISO,ISO IBA TAS,IEA,ISS ISO IEA,IBA,ISS ISO IEA,IDA IMP IBA IEA,IMP ISS ISO IEA,IMP ISO IEA,IEA,IMP,IDA,IMP IGI ISO IEA,IEA,ISO IEA,ISS ISO,IEA,IEA,ISS ISO IEA | ENSMUST00000132462,ENSMUST00000127047,ENSMUST00000153833,ENSMUST00000162069,ENSMUST00000121862,ENSMUST00000210871,ENSMUST00000034214,ENSMUST00000034215,ENSMUST00000124203,ENSMUST00000196375,ENSMUST00000020308,ENSMUST00000000095,ENSMUST00000017637,ENSMUST00000021413,ENSMUST00000221634,ENSMUST00000055458,ENSMUST00000114278,ENSMUST00000129905,ENSMUST00000051241,ENSMUST00000054002,ENSMUST00000078508,ENSMUST00000106248,ENSMUST00000093113,ENSMUST00000114795,ENSMUST00000025025,ENSMUST00000023829,ENSMUST00000049346,ENSMUST00000000808,ENSMUST00000034232,ENSMUST00000026552,ENSMUST00000102985,ENSMUST00000188073,ENSMUST00000040312,ENSMUST00000136850,ENSMUST00000050785,ENSMUST00000064265,ENSMUST00000125094,ENSMUST00000045921,ENSMUST00000091668,ENSMUST00000017841,ENSMUST00000015664,ENSMUST00000029846,ENSMUST00000051264,ENSMUST00000058610,ENSMUST00000029547,ENSMUST00000031314 |
| 9 | query_1 | TRUE | 0.0267515601776784 | 3173 | 161 | 38 | 0.236024844720497 | 0.0119760479041916 | GO:0070887 | GO:BP | cellular response to chemical stimulus | 27205 | 17294 | c("GO:0042221", "GO:0051716") | IMP,ISO IBA IEA,ISO IEA,IDA,IBA,ISO IBA,ISO IEA,ISS IBA,IDA IBA,IDA ISO,ISO IEA,IDA,IDA ISO IBA IEA,IDA,ISS ISO IEA,IDA,IDA IMP ISS ISO IEA,IDA IEP,IDA IEA,IDA IMP ISO IBA IEA,IEA,ISO IEA,IDA IBA,ISO IBA IEA,IBA,ISO IBA IEA,IDA,IMP,IDA,ISO,IDA,ISS ISO IBA IEA,IEA,IEA,IEA,IMP,ISO IBA IEA,IEA | ENSMUST00000132462,ENSMUST00000177197,ENSMUST00000107571,ENSMUST00000196245,ENSMUST00000153833,ENSMUST00000162069,ENSMUST00000210871,ENSMUST00000034214,ENSMUST00000034215,ENSMUST00000124203,ENSMUST00000196375,ENSMUST00000020308,ENSMUST00000021413,ENSMUST00000055458,ENSMUST00000114278,ENSMUST00000129905,ENSMUST00000051241,ENSMUST00000054002,ENSMUST00000106248,ENSMUST00000114795,ENSMUST00000105520,ENSMUST00000025025,ENSMUST00000013910,ENSMUST00000000808,ENSMUST00000034232,ENSMUST00000026552,ENSMUST00000040312,ENSMUST00000141380,ENSMUST00000136850,ENSMUST00000050785,ENSMUST00000149655,ENSMUST00000020699,ENSMUST00000066237,ENSMUST00000125094,ENSMUST00000015664,ENSMUST00000051264,ENSMUST00000058610,ENSMUST00000029547 |
| 10 | query_1 | TRUE | 0.0291471186056853 | 5997 | 161 | 59 | 0.366459627329193 | 0.00983825245956312 | GO:0048522 | GO:BP | positive regulation of cellular process | 27205 | 13543 | c("GO:0009987", "GO:0048518", "GO:0050794") | IMP,ISO IEA,ISO IEA,IDA ISO IEA,ISO IEA,IDA IMP ISO IEA,IMP,ISO,IMP ISO IBA,IBA,IMP ISO IEA,ISO,IDA,ISO IEA,IEA,ISS ISO,ISO IEA,IDA IMP IEA,IGI,IMP ISO IEA,IBA,ISO IEA,IDA IMP IGI ISO IEA,ISO IBA IEA,IMP ISS ISO IEA,IGI,IGI ISS ISO IBA IEA,ISS ISO IEA,IDA IMP ISS ISO IEA,IDA IMP ISS ISO IBA IEA,IDA IGI,IBA,IDA ISO IEA,IMP ISO,ISS ISO IEA,ISO,IMP IGI ISO IEA,IDA IGI ISS ISO IEA,ISO IEA,ISO IBA,IBA,IDA ISO IEA,IDA IMP ISO IEA,IMP,IDA ISO IEA,IDA,ISO,IDA IMP,IDA,TAS,IMP ISS ISO IEA,IMP,IMP ISO,IMP,IDA IGI ISO IEA,IBA,IDA IMP,ISO IEA,IDA IMP ISO IEA | ENSMUST00000132462,ENSMUST00000072631,ENSMUST00000163691,ENSMUST00000096232,ENSMUST00000177197,ENSMUST00000107571,ENSMUST00000127047,ENSMUST00000153833,ENSMUST00000162069,ENSMUST00000121862,ENSMUST00000210871,ENSMUST00000124203,ENSMUST00000204980,ENSMUST00000196375,ENSMUST00000027629,ENSMUST00000020308,ENSMUST00000108793,ENSMUST00000000095,ENSMUST00000017637,ENSMUST00000021413,ENSMUST00000221634,ENSMUST00000001497,ENSMUST00000138970,ENSMUST00000055458,ENSMUST00000114278,ENSMUST00000108313,ENSMUST00000121044,ENSMUST00000129905,ENSMUST00000051241,ENSMUST00000128241,ENSMUST00000054002,ENSMUST00000161459,ENSMUST00000078508,ENSMUST00000114795,ENSMUST00000028020,ENSMUST00000025025,ENSMUST00000023829,ENSMUST00000199615,ENSMUST00000061427,ENSMUST00000000808,ENSMUST00000034232,ENSMUST00000165487,ENSMUST00000019808,ENSMUST00000141380,ENSMUST00000136850,ENSMUST00000031646,ENSMUST00000050785,ENSMUST00000039164,ENSMUST00000149655,ENSMUST00000105393,ENSMUST00000064265,ENSMUST00000045921,ENSMUST00000090071,ENSMUST00000017841,ENSMUST00000029846,ENSMUST00000102975,ENSMUST00000051264,ENSMUST00000058610,ENSMUST00000029547 |
| 11 | query_1 | TRUE | 0.0305185681125186 | 2702 | 161 | 34 | 0.211180124223602 | 0.0125832716506292 | GO:0050793 | GO:BP | regulation of developmental process | 27205 | 14007 | c("GO:0032502", "GO:0050789") | IGI IBA,IDA IBA,IDA IMP ISO IEA,IMP,IMP,ISO IBA IEA,IMP ISO IEA,ISO,ISO IEA,IMP,IMP ISO IEA,IMP,ISO IBA IEA,IMP,ISO,ISS ISO IEA,IDA IMP ISS ISO IEA,IDA IGI,ISO IEA,IDA,IEA,IMP,ISO IEA,IBA,IMP,IMP,ISO IEA,ISO,IMP,IDA,IMP ISS ISO IEA,IMP ISO,IMP,IDA IMP ISO IEA | ENSMUST00000163691,ENSMUST00000096232,ENSMUST00000107571,ENSMUST00000127047,ENSMUST00000162069,ENSMUST00000121862,ENSMUST00000210871,ENSMUST00000124203,ENSMUST00000108793,ENSMUST00000000095,ENSMUST00000021413,ENSMUST00000138970,ENSMUST00000055458,ENSMUST00000108313,ENSMUST00000121044,ENSMUST00000129905,ENSMUST00000051241,ENSMUST00000054002,ENSMUST00000078508,ENSMUST00000106248,ENSMUST00000105520,ENSMUST00000023829,ENSMUST00000199615,ENSMUST00000167323,ENSMUST00000034232,ENSMUST00000040312,ENSMUST00000136850,ENSMUST00000050785,ENSMUST00000039164,ENSMUST00000149655,ENSMUST00000064265,ENSMUST00000017841,ENSMUST00000015664,ENSMUST00000029846 |
| 12 | query_1 | TRUE | 0.0398238981866993 | 6489 | 161 | 62 | 0.385093167701863 | 0.00955463091385421 | GO:1901564 | GO:BP | organonitrogen compound metabolic process | 27205 | 22563 | c("GO:0006807", "GO:0071704") | IDA IMP ISO IEA,IBA IEA,IDA ISO IEA,IMP ISO IEA,IDA IBA,IDA ISO IBA IEA,IDA ISO IBA TAS IEA,ISO IEA,ISS ISO,ISO IEA,ISO IEA,ISS IEA,ISO IEA,IBA IEA,IMP ISO IBA,IEA,ISS ISO IEA,IEA,IBA,ISS ISO IBA IEA,IBA,IDA IMP ISS ISO IEA,IEA,IDA ISO IEA,IBA IEA,IBA IEA,IEA,IMP IBA,IEA,IBA IEA,IBA IEA,IEA,IMP ISS ISO IEA,IDA IMP ISO IBA IEA,IDA ISO IEA,IDA ISO IEA,IEA,IDA IBA IEA,IEA,IMP,IBA IEA,IDA ISS ISO IBA TAS IC IEA,IDA IGI,IMP ISS ISO IEA,ISO IBA IEA,IPI ISO IBA IEA,ISO IEA,IDA ISO,IMP ISO IBA TAS IEA,IDA IMP ISO IBA TAS IEA,IMP ISS ISO IBA IEA,IBA,ISS ISO IBA IEA,IDA IGI ISO IEA,IBA,IDA,IDA,IEA,IBA IEA,IEA,IBA,IEA | ENSMUST00000132462,ENSMUST00000125346,ENSMUST00000097921,ENSMUST00000127047,ENSMUST00000162069,ENSMUST00000124203,ENSMUST00000204980,ENSMUST00000196375,ENSMUST00000020308,ENSMUST00000108793,ENSMUST00000021413,ENSMUST00000221634,ENSMUST00000001497,ENSMUST00000161069,ENSMUST00000002846,ENSMUST00000161674,ENSMUST00000114278,ENSMUST00000030677,ENSMUST00000121044,ENSMUST00000032157,ENSMUST00000210412,ENSMUST00000051241,ENSMUST00000128241,ENSMUST00000054002,ENSMUST00000161459,ENSMUST00000233870,ENSMUST00000121394,ENSMUST00000106248,ENSMUST00000093113,ENSMUST00000022460,ENSMUST00000171265,ENSMUST00000105520,ENSMUST00000025025,ENSMUST00000023829,ENSMUST00000199615,ENSMUST00000061427,ENSMUST00000167323,ENSMUST00000108353,ENSMUST00000107348,ENSMUST00000000808,ENSMUST00000188073,ENSMUST00000040312,ENSMUST00000136850,ENSMUST00000064265,ENSMUST00000066237,ENSMUST00000091668,ENSMUST00000023295,ENSMUST00000090071,ENSMUST00000028838,ENSMUST00000017841,ENSMUST00000015664,ENSMUST00000000573,ENSMUST00000057944,ENSMUST00000029846,ENSMUST00000102975,ENSMUST00000160534,ENSMUST00000174143,ENSMUST00000043400,ENSMUST00000058266,ENSMUST00000054107,ENSMUST00000106527,ENSMUST00000036426 |
| 13 | query_1 | TRUE | 0.0404287925160028 | 7674 | 161 | 70 | 0.434782608695652 | 0.00912170966901225 | GO:0007154 | GO:BP | cell communication | 27205 | 2781 | GO:0009987 | IMP ISS ISO IEA,IEA,IGI,EXP IDA IMP ISO IBA IEA,IBA IEA,IDA IMP ISO IBA IEA,IMP,IDA ISO IBA IEA,ISO IBA,IMP,IMP,IMP,ISO IBA,ISO IEA,IMP ISS ISO IBA IEA,IDA ISO IBA IEA,IDA IMP,IGI IBA,IDA IMP ISO IEA,ISS,ISO IEA,IBA,ISS ISO IEA,IEA,IDA IGI ISO IBA IEA,IBA,IMP,IDA IMP ISO IEA,IDA IGI ISO IEA,IEA,IMP IBA IEA,IDA IGI IEA,IDA IMP ISO IBA IEA,IMP,IBA IEA,IDA IBA,IDA ISS ISO IBA IEA,IEA,IMP ISS ISO IBA IEA,IDA IMP ISS ISO IEA,IDA IBA IEA,IDA IGI ISS ISO IEA,ISO IEA,IDA,ISO IBA IEA,IBA TAS,IDA,IBA IEA,ISO IBA IEA,ISS ISO IEA,IDA IGI IEA,IBA,IDA,IDA IMP ISO IBA IEA,IDA,IBA NAS,IMP ISO IEA,ISS ISO IBA IEA,IDA,NAS,IMP ISO IBA IEA,IEA,ISO IBA IEA,ISS ISO IBA IEA,IEA,IEA,IMP ISS ISO,IDA ISO IBA TAS IEA,IEA,ISS ISO IEA | ENSMUST00000132462,ENSMUST00000093942,ENSMUST00000044752,ENSMUST00000177197,ENSMUST00000125346,ENSMUST00000107571,ENSMUST00000127047,ENSMUST00000196245,ENSMUST00000162069,ENSMUST00000210871,ENSMUST00000034214,ENSMUST00000034215,ENSMUST00000124203,ENSMUST00000196375,ENSMUST00000020308,ENSMUST00000108793,ENSMUST00000000095,ENSMUST00000017637,ENSMUST00000021413,ENSMUST00000221634,ENSMUST00000001497,ENSMUST00000055458,ENSMUST00000114278,ENSMUST00000030677,ENSMUST00000121044,ENSMUST00000110993,ENSMUST00000129905,ENSMUST00000051241,ENSMUST00000054002,ENSMUST00000078508,ENSMUST00000131920,ENSMUST00000106248,ENSMUST00000114795,ENSMUST00000238845,ENSMUST00000176249,ENSMUST00000171265,ENSMUST00000028020,ENSMUST00000105520,ENSMUST00000025025,ENSMUST00000023829,ENSMUST00000013910,ENSMUST00000199615,ENSMUST00000061427,ENSMUST00000108353,ENSMUST00000000808,ENSMUST00000034232,ENSMUST00000019808,ENSMUST00000029648,ENSMUST00000188073,ENSMUST00000040312,ENSMUST00000136850,ENSMUST00000146512,ENSMUST00000050785,ENSMUST00000039164,ENSMUST00000149655,ENSMUST00000105393,ENSMUST00000064265,ENSMUST00000020699,ENSMUST00000045921,ENSMUST00000235000,ENSMUST00000017841,ENSMUST00000015664,ENSMUST00000029846,ENSMUST00000102975,ENSMUST00000054107,ENSMUST00000029406,ENSMUST00000051264,ENSMUST00000058610,ENSMUST00000029547,ENSMUST00000031314 |
| 14 | query_1 | TRUE | 0.00431020593676538 | 22796 | 167 | 158 | 0.946107784431138 | 0.00693104053342692 | GO:0110165 | GO:CC | cellular anatomical entity | 27262 | 3449 | GO:0005575 | IDA ISO IBA TAS IEA,IDA IEA,ISO IBA IEA,IEA,ISO IBA TAS IEA,IEA,IDA ISO TAS IEA,IDA IBA IEA,IEA,ISO IEA,EXP IDA IMP ISO IBA IEA,IEA,IBA IEA,IDA IPI ISO IBA TAS IEA,IDA ISS ISO IBA IEA,IBA IEA,ISO IEA,IDA IMP ISO IBA IEA,IDA ISS ISO IBA IEA,IEA,IDA HDA IBA IEA,HDA ISO IBA IEA,ISO IC IEA,ISO IBA IEA,IDA ISO IBA TAS IEA,IDA ISO IBA IEA,IDA ISO IBA IEA,IDA ISS ISO IC IEA,ISO IBA IEA,ISS ISO IBA TAS,IDA ISS ISO IBA TAS,IEA,ISO IBA TAS IEA,IDA ISO IBA IEA,IDA ISO ISA IBA IEA,IBA,IDA ISO IBA IEA,IDA IBA IEA,IBA IEA,IDA ISS ISO IEA,IDA ISO IEA,IDA ISS ISO IEA,IDA HDA IBA IEA,IDA ISO IBA IEA,ISS ISO IBA IEA,ISO IEA,IDA ISO IBA IEA,IDA IBA IEA,ISS ISO IBA IEA,IEA,IDA ISO IBA IEA,ISO IEA,IEA,ISS ISO IBA IEA,IDA ISS ISO IBA IEA,IEA,IDA IPI IMP ISO IBA IEA,IBA,IDA ISS ISO IBA IEA,ISO TAS IEA,IEA,IBA IEA,IDA ISS ISO IEA,IDA IBA IEA,IDA ISS ISO IBA IEA,IDA ISS ISO IBA IEA,ISO IBA TAS,ISS ISO IBA IEA,IDA ISO TAS IEA,ISO IBA TAS IEA,IDA IBA IEA,ISO IBA TAS IEA,IEA,IDA IBA TAS IEA,IBA IEA,ISO IBA IEA,IDA ISO IEA,ISO IBA IEA,IEA,IDA ISS ISO IBA IEA,IBA IEA,IDA IEA,IDA ISS ISO IBA IEA,IEA,IBA,ISS ISO IBA IEA,IDA ISS ISO IBA IEA,IDA IBA IEA,IDA ISO IBA IEA,ISO IEA,ISS ISO IBA IEA,IDA ISO IBA TAS IEA,IDA IBA IEA,IEA,IDA ISO IBA IEA,ISO IBA IEA,IDA IBA IEA,IEA,IDA IBA IEA,IDA IBA IEA,IDA ISO IBA TAS IEA,IBA IEA,IDA ISS ISO IBA IEA,IDA ISO IBA IEA,IDA ISO IBA IEA,ISS ISO IBA IEA,IDA ISS ISO IBA IEA,IBA IEA,IBA TAS,IDA ISO IBA TAS IEA,IDA IEA,IDA ISO IEA,ISO IBA IEA,IDA IEA,IDA HDA ISS ISO IBA TAS IEA,IEA,IDA ISO IBA IEA,IDA IMP ISS ISO IEA,IEA,IDA ISO IBA NAS IEA,ISS ISO IEA,ISS ISO IBA IEA,IDA ISO IBA IEA,IEA,HDA ISS ISO IBA IEA,IDA ISO IBA IEA,IDA HDA ISO IBA IEA,IDA ISO TAS IEA,IDA ISS IBA IEA,IDA IPI HDA ISO IEA,IDA ISS ISO IBA IEA,IDA ISS ISO IBA TAS IEA,IDA IBA IEA,ISO IBA,IDA ISO IBA IEA,IDA ISO IEA,IDA ISO IBA IEA,IDA ISO IBA IEA,ISS ISO IBA IEA,HDA IBA IEA,IBA,IDA ISO IEA,IEA,IDA ISO ISA NAS IEA,IEA,IEA,IBA IEA,IDA HDA ISO IBA TAS IEA,ISO IBA IEA,IDA ISO IBA IEA,HDA IEA,IEA,IEA,IBA IEA,ISO IBA IEA,ISO IBA TAS IEA,IDA HDA ISO IBA IEA,IEA | ENSMUST00000132462,ENSMUST00000093942,ENSMUST00000072631,ENSMUST00000183431,ENSMUST00000163691,ENSMUST00000229842,ENSMUST00000096232,ENSMUST00000231584,ENSMUST00000135217,ENSMUST00000044752,ENSMUST00000177197,ENSMUST00000125346,ENSMUST00000173623,ENSMUST00000176059,ENSMUST00000107571,ENSMUST00000052478,ENSMUST00000097921,ENSMUST00000127047,ENSMUST00000196245,ENSMUST00000201345,ENSMUST00000188970,ENSMUST00000184143,ENSMUST00000153833,ENSMUST00000151464,ENSMUST00000162069,ENSMUST00000138092,ENSMUST00000121862,ENSMUST00000210871,ENSMUST00000212405,ENSMUST00000034214,ENSMUST00000034215,ENSMUST00000170664,ENSMUST00000124203,ENSMUST00000204980,ENSMUST00000196375,ENSMUST00000042993,ENSMUST00000033310,ENSMUST00000027629,ENSMUST00000208001,ENSMUST00000020308,ENSMUST00000108793,ENSMUST00000000095,ENSMUST00000017637,ENSMUST00000021413,ENSMUST00000221634,ENSMUST00000001497,ENSMUST00000161069,ENSMUST00000138970,ENSMUST00000002846,ENSMUST00000161674,ENSMUST00000055458,ENSMUST00000114278,ENSMUST00000029440,ENSMUST00000108313,ENSMUST00000121044,ENSMUST00000032157,ENSMUST00000108458,ENSMUST00000110993,ENSMUST00000129905,ENSMUST00000051091,ENSMUST00000174695,ENSMUST00000174063,ENSMUST00000051241,ENSMUST00000128241,ENSMUST00000079590,ENSMUST00000039388,ENSMUST00000054002,ENSMUST00000161459,ENSMUST00000078508,ENSMUST00000233870,ENSMUST00000073536,ENSMUST00000131920,ENSMUST00000121394,ENSMUST00000106248,ENSMUST00000093113,ENSMUST00000200534,ENSMUST00000114795,ENSMUST00000022460,ENSMUST00000178238,ENSMUST00000238845,ENSMUST00000176249,ENSMUST00000171265,ENSMUST00000028020,ENSMUST00000105520,ENSMUST00000238402,ENSMUST00000025025,ENSMUST00000023829,ENSMUST00000013910,ENSMUST00000002908,ENSMUST00000108917,ENSMUST00000098273,ENSMUST00000199615,ENSMUST00000038684,ENSMUST00000053263,ENSMUST00000049346,ENSMUST00000061427,ENSMUST00000032127,ENSMUST00000167323,ENSMUST00000108353,ENSMUST00000107348,ENSMUST00000000808,ENSMUST00000034232,ENSMUST00000026552,ENSMUST00000165487,ENSMUST00000172854,ENSMUST00000102985,ENSMUST00000019808,ENSMUST00000029648,ENSMUST00000188073,ENSMUST00000040312,ENSMUST00000141380,ENSMUST00000136850,ENSMUST00000146512,ENSMUST00000031646,ENSMUST00000050785,ENSMUST00000208216,ENSMUST00000039164,ENSMUST00000149655,ENSMUST00000123490,ENSMUST00000105393,ENSMUST00000064265,ENSMUST00000020699,ENSMUST00000066237,ENSMUST00000140122,ENSMUST00000125094,ENSMUST00000045921,ENSMUST00000091668,ENSMUST00000023295,ENSMUST00000025002,ENSMUST00000024944,ENSMUST00000235000,ENSMUST00000015234,ENSMUST00000090071,ENSMUST00000028838,ENSMUST00000017841,ENSMUST00000094218,ENSMUST00000015664,ENSMUST00000000573,ENSMUST00000057944,ENSMUST00000029846,ENSMUST00000102975,ENSMUST00000160534,ENSMUST00000050780,ENSMUST00000174143,ENSMUST00000043400,ENSMUST00000035244,ENSMUST00000058266,ENSMUST00000106527,ENSMUST00000026681,ENSMUST00000005607,ENSMUST00000036426,ENSMUST00000041142,ENSMUST00000029406,ENSMUST00000051264,ENSMUST00000058610,ENSMUST00000029547,ENSMUST00000031314,ENSMUST00000110160 |
| 15 | query_1 | TRUE | 0.00464866794216382 | 2694 | 167 | 35 | 0.209580838323353 | 0.0129918337045286 | GO:0005576 | GO:CC | extracellular region | 27262 | 198 | GO:0110165 | IEA,IEA,IBA IEA,IDA ISO IEA,IDA HDA IBA IEA,IEA,IDA ISO IBA IEA,IBA,ISO,IBA,IEA,IEA,ISO IEA,ISO IEA,IDA ISS ISO IBA IEA,IEA,IDA ISO TAS IEA,ISO IEA,IDA IBA IEA,IEA,IBA IEA,IEA,IEA,IDA HDA ISS ISO IBA TAS IEA,IEA,HDA ISS ISO IBA IEA,IDA ISO IBA IEA,IDA ISO IBA IEA,ISO,IDA ISO IBA IEA,IDA IBA,IEA,IEA,IBA IEA,IDA ISO IEA | ENSMUST00000183431,ENSMUST00000044752 (Nrtn (neurturin) ,ENSMUST00000188970,ENSMUST00000210871 (hmgb2),ENSMUST00000017637 (Insulin-like growth factor-binding protein 4),ENSMUST00000029440,ENSMUST00000121044 (btc),ENSMUST00000110993 (Tgfbr3l),ENSMUST00000051091,ENSMUST00000174063 (h2-t24),ENSMUST00000121394,ENSMUST00000093113,ENSMUST00000200534 (gc vitamin binding protein),ENSMUST00000114795, (enpp1),ENSMUST00000105520,ENSMUST00000199615 (egf),ENSMUST00000061427,ENSMUST00000032127 (gastrokine3),ENSMUST00000167323 (polipoprotein L domain containing 1),ENSMUST00000034232 (Ccl17),ENSMUST00000172854,ENSMUST00000136850 (IL33),ENSMUST00000050785 (lcn2),ENSMUST00000064265 (pla2g3 ),ENSMUST00000125094 (glpx3 oxidative stress protein in dm),ENSMUST00000091668,ENSMUST00000015234 (Prostaglandin D2 synthase - płynotok pourazowy),ENSMUST00000017841,ENSMUST00000015664,ENSMUST00000000573,ENSMUST00000029846,ENSMUST00000036426,ENSMUST00000058610,ENSMUST00000031314 |
| 16 | query_1 | TRUE | 0.000162502059070664 | 10491 | 163 | 100 | 0.613496932515337 | 0.00953197979220284 | GO:0005515 | GO:MF | protein binding | 25177 | 1496 | GO:0005488 | IPI ISO IBA IEA,IPI,IEA,IPI,IPI IBA,IDA ISO IBA IEA,IEA,IPI IEA,IPI ISO IEA,IPI IEA,IPI ISO IEA,IDA IPI ISO IBA IEA,IEA,IPI IEA,ISO IEA,IPI ISO IBA,IPI ISO IEA,IEA,IDA IPI ISO IEA,IPI ISO,IPI ISO,IPI ISO IEA,IPI,IEA,IPI,IBA IEA,IDA IBA,IPI,IPI IBA IEA,IEA,IPI ISO IBA IEA,ISO IEA,IPI ISO,ISO IBA IEA,IEA,IPI ISO IEA,IDA IGI ISO IBA IEA,IDA IPI IBA IEA,IBA,IEA,ISO IEA,IEA,IDA IPI ISS ISO IEA,IPI ISS ISO IBA IEA,IDA IBA IEA,IPI IMP,IEA,IPI,IPI ISO IEA,IDA IPI IBA IEA,ISO IEA,IDA IPI ISO IEA,ISS ISO IEA,IPI ISO IBA IEA,ISS ISO IEA,IBA,IPI ISO IEA,IEA,IDA IPI ISO,IDA IPI ISO IEA,IDA IBA,ISO IBA IEA,ISO IBA IEA,IDA IPI ISO IEA,IDA IPI IBA,IPI ISO IBA IEA,ISO IBA TAS IEA,ISS ISO IEA,IPI,IEA,IPI ISO IEA,IPI ISO IEA,ISO IBA IEA,IDA IPI ISS ISO IBA IEA,IPI IEA,IDA IPI ISO IBA IEA,ISO,IPI,IPI ISO,ISO,ISO IEA,IPI ISO IBA TAS,ISO IEA,ISO IEA,ISS ISO IEA,IPI,IPI,IPI,IPI ISS ISO,ISO,ISO IEA,IDA IBA IEA,IBA,IEA,IDA IBA,IEA,IDA IPI ISO,IPI ISO IBA TAS IEA,ISO IEA,IEA | ENSMUST00000132462,ENSMUST00000093942,ENSMUST00000229842,ENSMUST00000096232,ENSMUST00000231584,ENSMUST00000044752,ENSMUST00000125346,ENSMUST00000176059,ENSMUST00000107571,ENSMUST00000052478,ENSMUST00000097921,ENSMUST00000196245,ENSMUST00000188970,ENSMUST00000153833,ENSMUST00000151464,ENSMUST00000162069,ENSMUST00000138092,ENSMUST00000121862,ENSMUST00000210871,ENSMUST00000124203,ENSMUST00000204980,ENSMUST00000196375,ENSMUST00000042993,ENSMUST00000033310,ENSMUST00000027629,ENSMUST00000208001,ENSMUST00000020308,ENSMUST00000000095,ENSMUST00000017637,ENSMUST00000140447,ENSMUST00000021413,ENSMUST00000001497,ENSMUST00000161069,ENSMUST00000002846,ENSMUST00000161674,ENSMUST00000055458,ENSMUST00000121044,ENSMUST00000108458,ENSMUST00000110993,ENSMUST00000210412,ENSMUST00000051091,ENSMUST00000174695,ENSMUST00000051241,ENSMUST00000128241,ENSMUST00000079590,ENSMUST00000054002,ENSMUST00000161459,ENSMUST00000078508,ENSMUST00000233870,ENSMUST00000073536,ENSMUST00000131920,ENSMUST00000106248,ENSMUST00000200534,ENSMUST00000114795,ENSMUST00000238845,ENSMUST00000176249,ENSMUST00000028020,ENSMUST00000105520,ENSMUST00000025025,ENSMUST00000023829,ENSMUST00000013910,ENSMUST00000108917,ENSMUST00000098273,ENSMUST00000199615,ENSMUST00000038684,ENSMUST00000000808,ENSMUST00000034232,ENSMUST00000026552,ENSMUST00000165487,ENSMUST00000172854,ENSMUST00000102985,ENSMUST00000019808,ENSMUST00000188073,ENSMUST00000040312,ENSMUST00000141380,ENSMUST00000136850,ENSMUST00000146512,ENSMUST00000031646,ENSMUST00000050785,ENSMUST00000039164,ENSMUST00000149655,ENSMUST00000105393,ENSMUST00000020699,ENSMUST00000066237,ENSMUST00000125094,ENSMUST00000091668,ENSMUST00000023295,ENSMUST00000024944,ENSMUST00000235000,ENSMUST00000028838,ENSMUST00000015664,ENSMUST00000029846,ENSMUST00000058266,ENSMUST00000054107,ENSMUST00000005607,ENSMUST00000221069,ENSMUST00000051264,ENSMUST00000058610,ENSMUST00000031314,ENSMUST00000110160 |
| 17 | query_1 | TRUE | 0.00783201670217198 | 8 | 163 | 3 | 0.0184049079754601 | 0.375 | GO:0005527 | GO:MF | macrolide binding | 25177 | 1506 | c("GO:0097159", "GO:1901363") | IEA,ISS ISO,ISS ISO IEA | ENSMUST00000233870,ENSMUST00000050785,ENSMUST00000031314 |
| 18 | query_1 | TRUE | 0.0105871982940873 | 16296 | 163 | 130 | 0.797546012269939 | 0.0079774177712322 | GO:0005488 | GO:MF | binding | 25177 | 1484 | GO:0003674 | IDA IPI ISO IBA IEA,IPI,ISO IBA IEA,ISO IBA IEA,IEA,IDA IPI ISO IBA IEA,IPI IBA,IDA ISO IBA IEA,IEA,ISO IBA IEA,IDA IPI IEA,IPI ISO IEA,IDA IPI ISO IBA IEA,IPI ISO IEA,IDA IPI ISO IBA IEA,IEA,IPI ISO IBA IEA,ISO IEA,IPI ISO IBA,IPI ISO IEA,IDA ISO IBA IEA,IDA IPI ISS ISO IBA IEA,ISS ISO IBA TAS IEA,IDA ISS ISO IBA TAS IEA,IEA,IPI ISO IEA,IPI ISO,IPI ISO IEA,IPI IEA,IEA,IDA IPI ISO IBA IEA,IBA IEA,IDA IBA,IDA IPI IMP ISO IBA IEA,IPI IBA IEA,IEA,IPI ISO IBA IEA,ISO IEA,IPI ISO IBA IEA,ISO IEA,IDA ISO IBA IEA,IEA,IPI ISO IEA,ISS ISO IBA IEA,IEA,IEA,IEA,IDA IGI ISO IBA IEA,IDA IPI IBA IEA,IBA,IEA,IEA,ISO IBA IEA,IEA,IDA IPI ISS ISO IEA,IPI ISS ISO IBA IEA,IDA IBA IEA,IBA IEA,IDA IPI IMP,IEA,IDA IPI ISS IBA IEA,IPI ISO IEA,IDA IPI IBA IEA,ISO IEA,IDA IPI ISO IEA,IEA,ISS ISO IBA IEA,IPI ISO IBA IEA,IEA,ISS ISO IEA,ISO IBA IEA,IEA,IPI ISO IEA,IEA,IDA IPI ISO,IDA IPI ISO IEA,IDA IBA,ISO IBA IEA,ISO IBA IEA,IDA IPI ISO IEA,IDA IPI IBA IEA,IEA,IBA IEA,IEA,IEA,IPI ISO IBA IEA,ISO IBA TAS IEA,ISS ISO IBA IEA,IPI,IEA,IPI ISO IBA IEA,IPI ISO IEA,ISO IBA IEA,IDA IPI ISS ISO IBA IEA,IPI IEA,IDA IPI ISO IBA IEA,ISO,IPI IEA,IEA,IDA IPI ISS ISO IEA,ISO,ISO IEA,IPI ISO IBA TAS,IEA,ISS ISO IBA IEA,ISO IEA,ISS ISO IBA IEA,IPI,IPI ISS ISO IEA,IPI ISO IEA,IDA IPI ISS ISO IBA TAS IEA,IDA ISS ISO IEA,IDA IBA IEA,ISO IEA,IDA IMP ISO IEA,ISO IEA,IBA IEA,IDA IBA IEA,IEA,IBA IEA,IEA,IEA,ISS ISO IEA,IDA IBA,IEA,IDA IPI IMP ISO IEA,IPI ISO IBA TAS IEA,IDA ISO IBA IEA,ISS ISO IBA IEA,IEA | ENSMUST00000132462,ENSMUST00000093942,ENSMUST00000072631,ENSMUST00000163691,ENSMUST00000229842,ENSMUST00000096232,ENSMUST00000231584,ENSMUST00000044752,ENSMUST00000125346,ENSMUST00000173623,ENSMUST00000176059,ENSMUST00000107571,ENSMUST00000052478,ENSMUST00000097921,ENSMUST00000196245,ENSMUST00000188970,ENSMUST00000153833,ENSMUST00000151464,ENSMUST00000162069,ENSMUST00000138092,ENSMUST00000121862,ENSMUST00000210871,ENSMUST00000034214,ENSMUST00000034215,ENSMUST00000170664,ENSMUST00000124203,ENSMUST00000204980,ENSMUST00000196375,ENSMUST00000042993,ENSMUST00000033310,ENSMUST00000027629,ENSMUST00000208001,ENSMUST00000020308,ENSMUST00000000095,ENSMUST00000017637,ENSMUST00000140447,ENSMUST00000021413,ENSMUST00000001497,ENSMUST00000161069,ENSMUST00000138970,ENSMUST00000002846,ENSMUST00000161674,ENSMUST00000055458,ENSMUST00000108313,ENSMUST00000030677,ENSMUST00000227683,ENSMUST00000008451,ENSMUST00000121044,ENSMUST00000108458,ENSMUST00000110993,ENSMUST00000210412,ENSMUST00000129905,ENSMUST00000051091,ENSMUST00000174695,ENSMUST00000051241,ENSMUST00000128241,ENSMUST00000079590,ENSMUST00000039388,ENSMUST00000054002,ENSMUST00000161459,ENSMUST00000078508,ENSMUST00000233870,ENSMUST00000073536,ENSMUST00000131920,ENSMUST00000106248,ENSMUST00000093113,ENSMUST00000200534,ENSMUST00000114795,ENSMUST00000022460,ENSMUST00000238845,ENSMUST00000176249,ENSMUST00000171265,ENSMUST00000028020,ENSMUST00000105520,ENSMUST00000025025,ENSMUST00000023829,ENSMUST00000013910,ENSMUST00000108917,ENSMUST00000098273,ENSMUST00000199615,ENSMUST00000038684,ENSMUST00000061427,ENSMUST00000167323,ENSMUST00000108353,ENSMUST00000107348,ENSMUST00000000808,ENSMUST00000034232,ENSMUST00000026552,ENSMUST00000165487,ENSMUST00000172854,ENSMUST00000102985,ENSMUST00000019808,ENSMUST00000188073,ENSMUST00000040312,ENSMUST00000141380,ENSMUST00000136850,ENSMUST00000146512,ENSMUST00000031646,ENSMUST00000150695,ENSMUST00000050785,ENSMUST00000039164,ENSMUST00000149655,ENSMUST00000105393,ENSMUST00000064265,ENSMUST00000020699,ENSMUST00000066237,ENSMUST00000125094,ENSMUST00000091668,ENSMUST00000023295,ENSMUST00000024944,ENSMUST00000235000,ENSMUST00000015234,ENSMUST00000090071,ENSMUST00000028838,ENSMUST00000017841,ENSMUST00000015664,ENSMUST00000000573,ENSMUST00000029846,ENSMUST00000102975,ENSMUST00000058266,ENSMUST00000054107,ENSMUST00000106527,ENSMUST00000026681,ENSMUST00000005607,ENSMUST00000221069,ENSMUST00000051264,ENSMUST00000058610,ENSMUST00000029547,ENSMUST00000031314,ENSMUST00000110160 |
| 19 | query_1 | TRUE | 0.0223974700706989 | 2 | 163 | 2 | 0.0122699386503067 | 1 | GO:1903981 | GO:MF | enterobactin binding | 25177 | 11045 | c("GO:0005527", "GO:0043168") | ISS ISO,ISS ISO IEA | ENSMUST00000050785,ENSMUST00000031314 |
| 20 | query_1 | TRUE | 0.043161290175529 | 275 | 163 | 9 | 0.0552147239263804 | 0.0327272727272727 | GO:0019207 | GO:MF | kinase regulator activity | 25177 | 3679 | GO:0030234 | IDA ISO IEA,IDA IGI ISO IEA,IBA,IDA ISO IBA IEA,ISS IBA IEA,IDA ISO IEA,IBA,IEA,IBA | ENSMUST00000097921,ENSMUST00000121044,ENSMUST00000054002,ENSMUST00000023829,ENSMUST00000098273,ENSMUST00000199615,ENSMUST00000188073,ENSMUST00000040312,ENSMUST00000102975 |
| 21 | query_1 | TRUE | 0.0306677387447952 | 54 | 34 | 5 | 0.147058823529412 | 0.0925925925925926 | HP:0000777 | HP | Abnormality of the thymus | 4965 | 608 | c("HP:0000818", "HP:0100763") | HP,HP,HP,HP,HP | ENSMUST00000097921,ENSMUST00000000095,ENSMUST00000023829,ENSMUST00000102985,ENSMUST00000017841 |
| 22 | query_1 | TRUE | 0.0451014475806717 | 359 | 72 | 10 | 0.138888888888889 | 0.0278551532033426 | KEGG:04151 | KEGG | PI3K-Akt signaling pathway | 8947 | 279 | KEGG:00000 | KEGG,KEGG,KEGG,KEGG,KEGG,KEGG,KEGG,KEGG,KEGG,KEGG | ENSMUST00000177197,ENSMUST00000125346,ENSMUST00000107571,ENSMUST00000124203,ENSMUST00000020308,ENSMUST00000171265,ENSMUST00000023829,ENSMUST00000199615,ENSMUST00000039164,ENSMUST00000029547 |
| 23 | query_1 | TRUE | 0.000167216820534954 | 4412 | 171 | 64 | 0.374269005847953 | 0.014505893019039 | TF:M00257_1 | TF | Factor: RREB-1; motif: CCCCAAACMMCCCC; match class: 1 | 21866 | 2935 | TF:M00257 | TF,TF,TF,TF,TF,TF,TF,TF,TF,TF,TF,TF,TF,TF,TF,TF,TF,TF,TF,TF,TF,TF,TF,TF,TF,TF,TF,TF,TF,TF,TF,TF,TF,TF,TF,TF,TF,TF,TF,TF,TF,TF,TF,TF,TF,TF,TF,TF,TF,TF,TF,TF,TF,TF,TF,TF,TF,TF,TF,TF,TF,TF,TF,TF | ENSMUST00000163691,ENSMUST00000229842,ENSMUST00000135217,ENSMUST00000044752,ENSMUST00000125346,ENSMUST00000107571,ENSMUST00000196245,ENSMUST00000188970,ENSMUST00000184143,ENSMUST00000153833,ENSMUST00000138092,ENSMUST00000121862,ENSMUST00000034215,ENSMUST00000170664,ENSMUST00000196375,ENSMUST00000042993,ENSMUST00000017637,ENSMUST00000140447,ENSMUST00000001497,ENSMUST00000002846,ENSMUST00000161674,ENSMUST00000114278,ENSMUST00000108313,ENSMUST00000032157,ENSMUST00000174063,ENSMUST00000078508,ENSMUST00000131920,ENSMUST00000093113,ENSMUST00000171265,ENSMUST00000201734,ENSMUST00000025025,ENSMUST00000013910,ENSMUST00000002908,ENSMUST00000108917,ENSMUST00000098273,ENSMUST00000038684,ENSMUST00000049346,ENSMUST00000061427,ENSMUST00000108353,ENSMUST00000000808,ENSMUST00000128007,ENSMUST00000165487,ENSMUST00000172854,ENSMUST00000040312,ENSMUST00000141380,ENSMUST00000050785,ENSMUST00000208216,ENSMUST00000105393,ENSMUST00000045921,ENSMUST00000091668,ENSMUST00000090071,ENSMUST00000017841,ENSMUST00000094218,ENSMUST00000057944,ENSMUST00000160534,ENSMUST00000050780,ENSMUST00000035244,ENSMUST00000054107,ENSMUST00000106527,ENSMUST00000005607,ENSMUST00000036426,ENSMUST00000041142,ENSMUST00000029547,ENSMUST00000110160 |
| 24 | query_1 | TRUE | 0.00156593232160241 | 10750 | 171 | 115 | 0.672514619883041 | 0.0106976744186047 | TF:M00257 | TF | Factor: RREB-1; motif: CCCCAAACMMCCCC | 21866 | 2934 | TF:M00000 | TF,TF,TF,TF,TF,TF,TF,TF,TF,TF,TF,TF,TF,TF,TF,TF,TF,TF,TF,TF,TF,TF,TF,TF,TF,TF,TF,TF,TF,TF,TF,TF,TF,TF,TF,TF,TF,TF,TF,TF,TF,TF,TF,TF,TF,TF,TF,TF,TF,TF,TF,TF,TF,TF,TF,TF,TF,TF,TF,TF,TF,TF,TF,TF,TF,TF,TF,TF,TF,TF,TF,TF,TF,TF,TF,TF,TF,TF,TF,TF,TF,TF,TF,TF,TF,TF,TF,TF,TF,TF,TF,TF,TF,TF,TF,TF,TF,TF,TF,TF,TF,TF,TF,TF,TF,TF,TF,TF,TF,TF,TF,TF,TF,TF,TF | ENSMUST00000132462,ENSMUST00000072631,ENSMUST00000163691,ENSMUST00000229842,ENSMUST00000135217,ENSMUST00000044752,ENSMUST00000125346,ENSMUST00000107571,ENSMUST00000052478,ENSMUST00000196245,ENSMUST00000201345,ENSMUST00000188970,ENSMUST00000184143,ENSMUST00000153833,ENSMUST00000162069,ENSMUST00000138092,ENSMUST00000121862,ENSMUST00000210871,ENSMUST00000034215,ENSMUST00000170664,ENSMUST00000196375,ENSMUST00000042993,ENSMUST00000033310,ENSMUST00000020308,ENSMUST00000108793,ENSMUST00000000095,ENSMUST00000017637,ENSMUST00000140447,ENSMUST00000021413,ENSMUST00000001497,ENSMUST00000002846,ENSMUST00000161674,ENSMUST00000055458,ENSMUST00000114278,ENSMUST00000029440,ENSMUST00000108313,ENSMUST00000030677,ENSMUST00000032157,ENSMUST00000110993,ENSMUST00000129905,ENSMUST00000174695,ENSMUST00000174063,ENSMUST00000051241,ENSMUST00000128241,ENSMUST00000054002,ENSMUST00000078508,ENSMUST00000073536,ENSMUST00000131920,ENSMUST00000121394,ENSMUST00000093113,ENSMUST00000114795,ENSMUST00000178238,ENSMUST00000238845,ENSMUST00000171265,ENSMUST00000028020,ENSMUST00000105520,ENSMUST00000238402,ENSMUST00000201734,ENSMUST00000025025,ENSMUST00000013910,ENSMUST00000002908,ENSMUST00000108917,ENSMUST00000098273,ENSMUST00000038684,ENSMUST00000053263,ENSMUST00000049346,ENSMUST00000061427,ENSMUST00000101254,ENSMUST00000167323,ENSMUST00000108353,ENSMUST00000000808,ENSMUST00000034232,ENSMUST00000128007,ENSMUST00000165487,ENSMUST00000172854,ENSMUST00000019808,ENSMUST00000029648,ENSMUST00000188073,ENSMUST00000040312,ENSMUST00000141380,ENSMUST00000146512,ENSMUST00000031646,ENSMUST00000150695,ENSMUST00000050785,ENSMUST00000208216,ENSMUST00000039164,ENSMUST00000123490,ENSMUST00000105393,ENSMUST00000020699,ENSMUST00000045921,ENSMUST00000091668,ENSMUST00000024944,ENSMUST00000235000,ENSMUST00000015234,ENSMUST00000090071,ENSMUST00000017841,ENSMUST00000094218,ENSMUST00000015664,ENSMUST00000057944,ENSMUST00000029846,ENSMUST00000160534,ENSMUST00000050780,ENSMUST00000174143,ENSMUST00000043400,ENSMUST00000035244,ENSMUST00000058266,ENSMUST00000054107,ENSMUST00000106527,ENSMUST00000005607,ENSMUST00000036426,ENSMUST00000041142,ENSMUST00000221069,ENSMUST00000029547,ENSMUST00000031314,ENSMUST00000110160 |
| 25 | query_1 | TRUE | 0.00192267884633426 | 14470 | 171 | 141 | 0.824561403508772 | 0.00974429854872149 | TF:M00378_1 | TF | Factor: Pax-4; motif: NNNNNYCACCCB; match class: 1 | 21866 | 2615 | TF:M00378 | TF,TF,TF,TF,TF,TF,TF,TF,TF,TF,TF,TF,TF,TF,TF,TF,TF,TF,TF,TF,TF,TF,TF,TF,TF,TF,TF,TF,TF,TF,TF,TF,TF,TF,TF,TF,TF,TF,TF,TF,TF,TF,TF,TF,TF,TF,TF,TF,TF,TF,TF,TF,TF,TF,TF,TF,TF,TF,TF,TF,TF,TF,TF,TF,TF,TF,TF,TF,TF,TF,TF,TF,TF,TF,TF,TF,TF,TF,TF,TF,TF,TF,TF,TF,TF,TF,TF,TF,TF,TF,TF,TF,TF,TF,TF,TF,TF,TF,TF,TF,TF,TF,TF,TF,TF,TF,TF,TF,TF,TF,TF,TF,TF,TF,TF,TF,TF,TF,TF,TF,TF,TF,TF,TF,TF,TF,TF,TF,TF,TF,TF,TF,TF,TF,TF,TF,TF,TF,TF,TF,TF | ENSMUST00000132462,ENSMUST00000093942,ENSMUST00000072631,ENSMUST00000163691,ENSMUST00000229842,ENSMUST00000096232,ENSMUST00000231584,ENSMUST00000135217,ENSMUST00000044752,ENSMUST00000177197,ENSMUST00000125346,ENSMUST00000173623,ENSMUST00000176059,ENSMUST00000107571,ENSMUST00000052478,ENSMUST00000097921,ENSMUST00000127047,ENSMUST00000196245,ENSMUST00000201345,ENSMUST00000188970,ENSMUST00000184143,ENSMUST00000153833,ENSMUST00000151464,ENSMUST00000162069,ENSMUST00000138092,ENSMUST00000121862,ENSMUST00000210871,ENSMUST00000212405,ENSMUST00000034214,ENSMUST00000124203,ENSMUST00000204980,ENSMUST00000042993,ENSMUST00000033310,ENSMUST00000027629,ENSMUST00000020308,ENSMUST00000108793,ENSMUST00000000095,ENSMUST00000017637,ENSMUST00000140447,ENSMUST00000021413,ENSMUST00000001497,ENSMUST00000161069,ENSMUST00000138970,ENSMUST00000002846,ENSMUST00000055458,ENSMUST00000114278,ENSMUST00000029440,ENSMUST00000108313,ENSMUST00000030677,ENSMUST00000227683,ENSMUST00000008451,ENSMUST00000032157,ENSMUST00000108458,ENSMUST00000110993,ENSMUST00000210412,ENSMUST00000129905,ENSMUST00000051091,ENSMUST00000174695,ENSMUST00000174063,ENSMUST00000051241,ENSMUST00000128241,ENSMUST00000079590,ENSMUST00000039388,ENSMUST00000054002,ENSMUST00000078508,ENSMUST00000073536,ENSMUST00000121394,ENSMUST00000106248,ENSMUST00000093113,ENSMUST00000022460,ENSMUST00000178238,ENSMUST00000238845,ENSMUST00000171265,ENSMUST00000028020,ENSMUST00000238402,ENSMUST00000201734,ENSMUST00000025025,ENSMUST00000023829,ENSMUST00000013910,ENSMUST00000002908,ENSMUST00000108917,ENSMUST00000098273,ENSMUST00000199615,ENSMUST00000038684,ENSMUST00000053263,ENSMUST00000049346,ENSMUST00000061427,ENSMUST00000167323,ENSMUST00000108353,ENSMUST00000000808,ENSMUST00000034232,ENSMUST00000026552,ENSMUST00000128007,ENSMUST00000165487,ENSMUST00000172854,ENSMUST00000019808,ENSMUST00000029648,ENSMUST00000188073,ENSMUST00000146512,ENSMUST00000031646,ENSMUST00000150695,ENSMUST00000050785,ENSMUST00000208216,ENSMUST00000039164,ENSMUST00000149655,ENSMUST00000123490,ENSMUST00000105393,ENSMUST00000064265,ENSMUST00000020699,ENSMUST00000066237,ENSMUST00000140122,ENSMUST00000125094,ENSMUST00000045921,ENSMUST00000091668,ENSMUST00000023295,ENSMUST00000024944,ENSMUST00000235000,ENSMUST00000015234,ENSMUST00000028838,ENSMUST00000017841,ENSMUST00000094218,ENSMUST00000015664,ENSMUST00000057944,ENSMUST00000029846,ENSMUST00000102975,ENSMUST00000160534,ENSMUST00000050780,ENSMUST00000174143,ENSMUST00000043400,ENSMUST00000238521,ENSMUST00000035244,ENSMUST00000058266,ENSMUST00000054107,ENSMUST00000106527,ENSMUST00000005607,ENSMUST00000036426,ENSMUST00000221069,ENSMUST00000051264,ENSMUST00000029547,ENSMUST00000031314,ENSMUST00000110160 |
| 26 | query_1 | TRUE | 0.00414241967974566 | 3177 | 171 | 48 | 0.280701754385965 | 0.0151085930122757 | TF:M10367_1 | TF | Factor: Smad3; motif: NNCTSNCWSCWS; match class: 1 | 21866 | 3057 | TF:M10367 | TF,TF,TF,TF,TF,TF,TF,TF,TF,TF,TF,TF,TF,TF,TF,TF,TF,TF,TF,TF,TF,TF,TF,TF,TF,TF,TF,TF,TF,TF,TF,TF,TF,TF,TF,TF,TF,TF,TF,TF,TF,TF,TF,TF,TF,TF,TF,TF | ENSMUST00000072631,ENSMUST00000229842,ENSMUST00000096232,ENSMUST00000044752,ENSMUST00000177197,ENSMUST00000125346,ENSMUST00000196245,ENSMUST00000153833,ENSMUST00000162069,ENSMUST00000170664,ENSMUST00000196375,ENSMUST00000042993,ENSMUST00000033310,ENSMUST00000027629,ENSMUST00000208001,ENSMUST00000020308,ENSMUST00000017637,ENSMUST00000140447,ENSMUST00000161069,ENSMUST00000138970,ENSMUST00000029440,ENSMUST00000108458,ENSMUST00000110993,ENSMUST00000079590,ENSMUST00000078508,ENSMUST00000073536,ENSMUST00000093113,ENSMUST00000022460,ENSMUST00000025025,ENSMUST00000013910,ENSMUST00000199615,ENSMUST00000053263,ENSMUST00000107348,ENSMUST00000172854,ENSMUST00000019808,ENSMUST00000146512,ENSMUST00000150695,ENSMUST00000050785,ENSMUST00000105393,ENSMUST00000045921,ENSMUST00000025002,ENSMUST00000235000,ENSMUST00000094218,ENSMUST00000174143,ENSMUST00000238521,ENSMUST00000035244,ENSMUST00000036426,ENSMUST00000029406 |
| 27 | query_1 | TRUE | 0.0141040957898754 | 10879 | 171 | 113 | 0.660818713450292 | 0.0103869840978031 | TF:M01113 | TF | Factor: CACD; motif: CCACRCCC | 21866 | 346 | TF:M00000 | TF,TF,TF,TF,TF,TF,TF,TF,TF,TF,TF,TF,TF,TF,TF,TF,TF,TF,TF,TF,TF,TF,TF,TF,TF,TF,TF,TF,TF,TF,TF,TF,TF,TF,TF,TF,TF,TF,TF,TF,TF,TF,TF,TF,TF,TF,TF,TF,TF,TF,TF,TF,TF,TF,TF,TF,TF,TF,TF,TF,TF,TF,TF,TF,TF,TF,TF,TF,TF,TF,TF,TF,TF,TF,TF,TF,TF,TF,TF,TF,TF,TF,TF,TF,TF,TF,TF,TF,TF,TF,TF,TF,TF,TF,TF,TF,TF,TF,TF,TF,TF,TF,TF,TF,TF,TF,TF,TF,TF,TF,TF,TF,TF | ENSMUST00000132462,ENSMUST00000072631,ENSMUST00000183431,ENSMUST00000163691,ENSMUST00000229842,ENSMUST00000096232,ENSMUST00000135217,ENSMUST00000044752,ENSMUST00000177197,ENSMUST00000125346,ENSMUST00000176059,ENSMUST00000107571,ENSMUST00000097921,ENSMUST00000127047,ENSMUST00000196245,ENSMUST00000201345,ENSMUST00000184143,ENSMUST00000153833,ENSMUST00000151464,ENSMUST00000138092,ENSMUST00000121862,ENSMUST00000212405,ENSMUST00000034214,ENSMUST00000170664,ENSMUST00000124203,ENSMUST00000196375,ENSMUST00000042993,ENSMUST00000033310,ENSMUST00000027629,ENSMUST00000208001,ENSMUST00000020308,ENSMUST00000108793,ENSMUST00000000095,ENSMUST00000017637,ENSMUST00000140447,ENSMUST00000021413,ENSMUST00000221634,ENSMUST00000001497,ENSMUST00000161069,ENSMUST00000002846,ENSMUST00000161674,ENSMUST00000055458,ENSMUST00000114278,ENSMUST00000029440,ENSMUST00000108313,ENSMUST00000030677,ENSMUST00000121044,ENSMUST00000108458,ENSMUST00000129905,ENSMUST00000051091,ENSMUST00000174695,ENSMUST00000174063,ENSMUST00000051241,ENSMUST00000128241,ENSMUST00000079590,ENSMUST00000039388,ENSMUST00000161459,ENSMUST00000078508,ENSMUST00000073536,ENSMUST00000093113,ENSMUST00000114795,ENSMUST00000238845,ENSMUST00000171265,ENSMUST00000025025,ENSMUST00000023829,ENSMUST00000013910,ENSMUST00000002908,ENSMUST00000108917,ENSMUST00000098273,ENSMUST00000199615,ENSMUST00000038684,ENSMUST00000053263,ENSMUST00000061427,ENSMUST00000101254,ENSMUST00000167323,ENSMUST00000108353,ENSMUST00000107348,ENSMUST00000000808,ENSMUST00000034232,ENSMUST00000026552,ENSMUST00000128007,ENSMUST00000019808,ENSMUST00000040312,ENSMUST00000141380,ENSMUST00000031646,ENSMUST00000050785,ENSMUST00000208216,ENSMUST00000039164,ENSMUST00000105393,ENSMUST00000064265,ENSMUST00000020699,ENSMUST00000140122,ENSMUST00000045921,ENSMUST00000023295,ENSMUST00000025002,ENSMUST00000235000,ENSMUST00000015234,ENSMUST00000017841,ENSMUST00000094218,ENSMUST00000015664,ENSMUST00000057944,ENSMUST00000029846,ENSMUST00000238521,ENSMUST00000058266,ENSMUST00000054107,ENSMUST00000005607,ENSMUST00000041142,ENSMUST00000029406,ENSMUST00000221069,ENSMUST00000051264,ENSMUST00000029547,ENSMUST00000031314,ENSMUST00000110160 |
| 28 | query_1 | TRUE | 0.0162305536978614 | 1742 | 171 | 31 | 0.181286549707602 | 0.0177956371986223 | TF:M10226_1 | TF | Factor: ER-alpha; motif: RGGTCASMNTGACCY; match class: 1 | 21866 | 993 | TF:M10226 | TF,TF,TF,TF,TF,TF,TF,TF,TF,TF,TF,TF,TF,TF,TF,TF,TF,TF,TF,TF,TF,TF,TF,TF,TF,TF,TF,TF,TF,TF,TF | ENSMUST00000093942,ENSMUST00000044752,ENSMUST00000173623,ENSMUST00000196245,ENSMUST00000201345,ENSMUST00000188970,ENSMUST00000210871,ENSMUST00000034215,ENSMUST00000124203,ENSMUST00000001497,ENSMUST00000008451,ENSMUST00000121044,ENSMUST00000129905,ENSMUST00000039388,ENSMUST00000233870,ENSMUST00000178238,ENSMUST00000238402,ENSMUST00000023829,ENSMUST00000013910,ENSMUST00000199615,ENSMUST00000053263,ENSMUST00000034232,ENSMUST00000165487,ENSMUST00000025002,ENSMUST00000090071,ENSMUST00000000573,ENSMUST00000043400,ENSMUST00000005607,ENSMUST00000221069,ENSMUST00000051264,ENSMUST00000029547 |
| 29 | query_1 | TRUE | 0.0410748607691748 | 7327 | 171 | 83 | 0.485380116959064 | 0.0113279650607343 | TF:M03944 | TF | Factor: Zfp740; motif: NCCCCCCCAC | 21866 | 4050 | TF:M00000 | TF,TF,TF,TF,TF,TF,TF,TF,TF,TF,TF,TF,TF,TF,TF,TF,TF,TF,TF,TF,TF,TF,TF,TF,TF,TF,TF,TF,TF,TF,TF,TF,TF,TF,TF,TF,TF,TF,TF,TF,TF,TF,TF,TF,TF,TF,TF,TF,TF,TF,TF,TF,TF,TF,TF,TF,TF,TF,TF,TF,TF,TF,TF,TF,TF,TF,TF,TF,TF,TF,TF,TF,TF,TF,TF,TF,TF,TF,TF,TF,TF,TF,TF | ENSMUST00000093942,ENSMUST00000072631,ENSMUST00000183431,ENSMUST00000163691,ENSMUST00000231584,ENSMUST00000135217,ENSMUST00000044752,ENSMUST00000177197,ENSMUST00000125346,ENSMUST00000176059,ENSMUST00000107571,ENSMUST00000097921,ENSMUST00000127047,ENSMUST00000196245,ENSMUST00000188970,ENSMUST00000184143,ENSMUST00000153833,ENSMUST00000151464,ENSMUST00000138092,ENSMUST00000210871,ENSMUST00000034214,ENSMUST00000034215,ENSMUST00000124203,ENSMUST00000196375,ENSMUST00000042993,ENSMUST00000033310,ENSMUST00000027629,ENSMUST00000020308,ENSMUST00000140447,ENSMUST00000021413,ENSMUST00000001497,ENSMUST00000161069,ENSMUST00000138970,ENSMUST00000161674,ENSMUST00000114278,ENSMUST00000108313,ENSMUST00000051241,ENSMUST00000128241,ENSMUST00000039388,ENSMUST00000054002,ENSMUST00000078508,ENSMUST00000073536,ENSMUST00000121394,ENSMUST00000093113,ENSMUST00000200534,ENSMUST00000238845,ENSMUST00000171265,ENSMUST00000201734,ENSMUST00000025025,ENSMUST00000023829,ENSMUST00000013910,ENSMUST00000002908,ENSMUST00000108917,ENSMUST00000098273,ENSMUST00000053263,ENSMUST00000049346,ENSMUST00000061427,ENSMUST00000167323,ENSMUST00000107348,ENSMUST00000000808,ENSMUST00000034232,ENSMUST00000128007,ENSMUST00000172854,ENSMUST00000019808,ENSMUST00000141380,ENSMUST00000150695,ENSMUST00000149655,ENSMUST00000105393,ENSMUST00000064265,ENSMUST00000020699,ENSMUST00000023295,ENSMUST00000017841,ENSMUST00000094218,ENSMUST00000057944,ENSMUST00000029846,ENSMUST00000035244,ENSMUST00000058266,ENSMUST00000026681,ENSMUST00000005607,ENSMUST00000036426,ENSMUST00000041142,ENSMUST00000029406,ENSMUST00000110160 |
