# Supplementary material for: Novel insights into neuropathy: The impact of prolonged hyperglycemia on long non-coding RNA expression
Source: PLoS One. 2025 Oct 27;20(10):e0334245. doi: 10.1371/journal.pone.0334245 (PMC12558608; doi:10.1371/journal.pone.0334245)
Supplement: S3 Table — (DOCX) [file pone.0334245.s003.docx]

| Supplementary Table 3. Trans-acting | | | | | | | | | | | | | | | | |
| --- | --- | --- | --- | --- | --- | --- | --- | --- | --- | --- | --- | --- | --- | --- | --- | --- |
|  | query | significant | p_value | term_size | query_size | intersection_size | precision | recall | term_id | source | term_name | effective_domain_size | source_order | parents | evidence_codes | intersection |
| 1 | query_1 | TRUE | 0.000509746683425827 | 6228 | 133 | 57 | 0.428571428571429 | 0.00915221579961464 | GO:0048856 | GO:BP | anatomical structure development | 27205 | 13840 | GO:0032502 | ISS ISO IEA,IDA IMP ISS ISO IEA,ISO IBA,IEA,ISS ISO,IDA IMP IGI ISS ISO IBA IEA,NAS,IMP ISO IEA,IEA,ISO IEA,IDA IMP IGI ISS ISO IEA,IMP,IMP,IEA,ISO IBA NAS IEA,ISS,IMP IGI IBA,IMP,IBA,IDA IMP,IMP,IMP ISO IEA,NAS IEA,ISO IBA IEA,IDA ISO IEA,IBA,IMP ISS ISO IEA,IMP ISO,IMP IEA,IMP TAS,IDA IGI ISO IEA,IDA,IDA IMP ISO IEA,IMP,IDA IMP ISO IBA IEA,IDA IMP ISO IEA,IEA,IMP,ISO IEA,IEA,ISO NAS,IEA,IEA,IBA,IMP,IMP,IMP ISS ISO IEA,IMP IBA,IEA,IDA IMP,IDA,IMP ISO IBA IEA,IBA,IMP ISO IEA,IMP,IMP,ISO IEA | ENSMUSG00000052688,ENSMUSG00000026697,ENSMUSG00000019970,ENSMUSG00000037370,ENSMUSG00000020108,ENSMUSG00000000731,ENSMUSG00000000732,ENSMUSG00000034579,ENSMUSG00000020182,ENSMUSG00000043448,ENSMUSG00000000093,ENSMUSG00000021025,ENSMUSG00000058669,ENSMUSG00000033715,ENSMUSG00000118537,ENSMUSG00000071350,ENSMUSG00000042622,ENSMUSG00000047034,ENSMUSG00000024190,ENSMUSG00000024215,ENSMUSG00000023067,ENSMUSG00000039481,ENSMUSG00000024430,ENSMUSG00000045664,ENSMUSG00000024810,ENSMUSG00000049134,ENSMUSG00000036587,ENSMUSG00000017697,ENSMUSG00000028111,ENSMUSG00000032854,ENSMUSG00000028017,ENSMUSG00000037994,ENSMUSG00000028195,ENSMUSG00000036832,ENSMUSG00000038668,ENSMUSG00000028655,ENSMUSG00000012123,ENSMUSG00000043635,ENSMUSG00000005057,ENSMUSG00000029797,ENSMUSG00000079495,ENSMUSG00000041377,ENSMUSG00000004328,ENSMUSG00000051965,ENSMUSG00000085601,ENSMUSG00000040714,ENSMUSG00000044786,ENSMUSG00000038763,ENSMUSG00000048782,ENSMUSG00000042828,ENSMUSG00000031004,ENSMUSG00000009248,ENSMUSG00000089736,ENSMUSG00000054717,ENSMUSG00000005470,ENSMUSG00000031780,ENSMUSG00000053399 |
| 2 | query_1 | TRUE | 0.00102351831091141 | 6865 | 133 | 60 | 0.451127819548872 | 0.00873998543335761 | GO:0032502 | GO:BP | developmental process | 27205 | 8032 | GO:0008150 | ISS ISO IEA,IDA IMP ISS ISO IBA IEA,ISO IBA,IEA,ISS ISO,IDA IMP IGI ISS ISO IBA IEA,NAS,IMP ISS ISO IEA,IEA,ISO IEA,IDA IMP IGI ISS ISO IBA IEA,IMP ISO IEA,IMP IBA,IEA,ISO IBA NAS IEA,ISS,IMP IGI IBA,IMP,IBA,IDA IMP IBA,IMP,IMP ISO IEA,NAS IEA,ISO IBA IEA,IDA ISO IEA,IBA,IMP ISS ISO IEA,IMP,IMP ISO,IMP IEA,IEA,IMP IEA,IMP TAS,IDA IGI ISO IEA,IDA,IDA IMP IGI ISO IEA,IMP,IDA IMP ISO IBA IEA,IDA IMP ISO IEA,IEA,IMP,IDA ISO IEA,IEA,ISO NAS,IEA,IEA,IMP IBA IEA,IMP,IMP IEA,IDA IMP ISS ISO IEA,IMP IBA,IEA,IDA IMP,IDA,IMP ISO IBA IEA,IBA,IMP ISO IEA,IMP,IMP,ISO IEA | ENSMUSG00000052688,ENSMUSG00000026697,ENSMUSG00000019970,ENSMUSG00000037370,ENSMUSG00000020108,ENSMUSG00000000731,ENSMUSG00000000732,ENSMUSG00000034579,ENSMUSG00000020182,ENSMUSG00000043448,ENSMUSG00000000093,ENSMUSG00000021025,ENSMUSG00000058669,ENSMUSG00000033715,ENSMUSG00000118537,ENSMUSG00000071350,ENSMUSG00000042622,ENSMUSG00000047034,ENSMUSG00000024190,ENSMUSG00000024215,ENSMUSG00000023067,ENSMUSG00000039481,ENSMUSG00000024430,ENSMUSG00000045664,ENSMUSG00000024810,ENSMUSG00000049134,ENSMUSG00000036587,ENSMUSG00000032715,ENSMUSG00000017697,ENSMUSG00000027938,ENSMUSG00000028141,ENSMUSG00000028111,ENSMUSG00000032854,ENSMUSG00000028017,ENSMUSG00000037994,ENSMUSG00000028195,ENSMUSG00000036832,ENSMUSG00000038668,ENSMUSG00000028655,ENSMUSG00000012123,ENSMUSG00000043635,ENSMUSG00000005057,ENSMUSG00000029797,ENSMUSG00000079495,ENSMUSG00000041377,ENSMUSG00000004328,ENSMUSG00000051965,ENSMUSG00000085601,ENSMUSG00000040714,ENSMUSG00000044786,ENSMUSG00000038763,ENSMUSG00000048782,ENSMUSG00000042828,ENSMUSG00000031004,ENSMUSG00000009248,ENSMUSG00000089736,ENSMUSG00000054717,ENSMUSG00000005470,ENSMUSG00000031780,ENSMUSG00000053399 |
| 3 | query_1 | TRUE | 0.00639857883836481 | 4873 | 133 | 46 | 0.345864661654135 | 0.00943977016211779 | GO:0007275 | GO:BP | multicellular organism development | 27205 | 2871 | c("GO:0032501", "GO:0048856") | ISS ISO IEA,IDA IMP ISS ISO IEA,ISO IBA,ISS ISO,IDA IMP ISS ISO IBA IEA,NAS,IMP ISO IEA,IEA,ISO IEA,IDA IMP IGI ISS ISO IEA,IMP,IMP,IEA,ISO IBA NAS IEA,ISS,IGI IBA,IBA,IDA IMP,IMP,IMP ISO IEA,IDA ISO IEA,IBA,ISS ISO IEA,IMP ISO,IMP,IMP TAS,IDA ISO IEA,IDA,IDA IMP ISO IEA,IMP,IDA IMP ISO IBA IEA,IDA IMP ISO IEA,IEA,IMP,ISO IEA,IEA,NAS,IEA,IMP ISS ISO IEA,IMP IBA,IEA,IDA,IMP ISO IBA IEA,IMP ISO IEA,IMP,ISO IEA | ENSMUSG00000052688,ENSMUSG00000026697,ENSMUSG00000019970,ENSMUSG00000020108,ENSMUSG00000000731,ENSMUSG00000000732,ENSMUSG00000034579,ENSMUSG00000020182,ENSMUSG00000043448,ENSMUSG00000000093,ENSMUSG00000021025,ENSMUSG00000058669,ENSMUSG00000033715,ENSMUSG00000118537,ENSMUSG00000071350,ENSMUSG00000042622,ENSMUSG00000024190,ENSMUSG00000024215,ENSMUSG00000023067,ENSMUSG00000039481,ENSMUSG00000024810,ENSMUSG00000049134,ENSMUSG00000036587,ENSMUSG00000017697,ENSMUSG00000028111,ENSMUSG00000032854,ENSMUSG00000028017,ENSMUSG00000037994,ENSMUSG00000028195,ENSMUSG00000036832,ENSMUSG00000038668,ENSMUSG00000028655,ENSMUSG00000012123,ENSMUSG00000043635,ENSMUSG00000005057,ENSMUSG00000029797,ENSMUSG00000079495,ENSMUSG00000004328,ENSMUSG00000044786,ENSMUSG00000038763,ENSMUSG00000048782,ENSMUSG00000031004,ENSMUSG00000009248,ENSMUSG00000054717,ENSMUSG00000005470,ENSMUSG00000053399 |
| 4 | query_1 | TRUE | 0.0101097176860165 | 9152 | 133 | 70 | 0.526315789473684 | 0.0076486013986014 | GO:0032501 | GO:BP | multicellular organismal process | 27205 | 8031 | GO:0008150 | ISS ISO IEA,IDA IMP ISS ISO IBA IEA,ISO IBA,IEA,ISS ISO,IDA IMP IGI ISS ISO IBA IEA,IMP IBA TAS NAS IEA,ISS ISO IBA IEA,IDA IMP ISS ISO IEA,IEA,ISO IEA,ISS ISO IEA,IDA IMP IGI ISS ISO IEA,IMP,IMP,IMP,IEA,IEA,IMP ISO IBA,ISO IBA NAS IEA,ISS,IGI IBA,IMP IGI ISO IEA,IEA,IBA,IDA IMP,IMP IGI,IMP ISO IEA,NAS IEA,IDA IGI ISO IBA IEA,IBA,IMP ISS ISO IEA,IDA IGI IEA,IMP IGI ISO IBA IEA,IMP,IMP IEA,IEA,IMP ISO IEA,IMP TAS,IDA ISO IEA,IEA,IDA,IDA IMP IGI ISO IEA,IMP,IMP IBA IEA,IDA IMP ISO IBA IEA,IDA IMP ISO IEA,IEA,IMP ISO IEA,IMP ISO IEA,IMP ISO IEA,IMP ISS ISO IEA,IEA,NAS,IEA,IMP IBA IEA,IMP,IMP IEA,IMP ISS ISO IEA,IMP IBA,IEA,IMP,IMP,IDA,IMP ISO IBA IEA,IMP ISO IEA,ISO IBA,IMP,IMP ISO IEA,ISA IBA IEA | ENSMUSG00000052688,ENSMUSG00000026697,ENSMUSG00000019970,ENSMUSG00000037370,ENSMUSG00000020108,ENSMUSG00000000731,ENSMUSG00000000732,ENSMUSG00000025401,ENSMUSG00000034579,ENSMUSG00000020182,ENSMUSG00000043448,ENSMUSG00000020893,ENSMUSG00000000093,ENSMUSG00000037336,ENSMUSG00000021025,ENSMUSG00000058669,ENSMUSG00000044988,ENSMUSG00000033715,ENSMUSG00000044734,ENSMUSG00000118537,ENSMUSG00000071350,ENSMUSG00000042622,ENSMUSG00000022622,ENSMUSG00000022766,ENSMUSG00000024190,ENSMUSG00000024215,ENSMUSG00000023067,ENSMUSG00000039481,ENSMUSG00000024430,ENSMUSG00000024810,ENSMUSG00000049134,ENSMUSG00000036587,ENSMUSG00000015090,ENSMUSG00000017697,ENSMUSG00000109293,ENSMUSG00000027938,ENSMUSG00000028141,ENSMUSG00000028111,ENSMUSG00000032854,ENSMUSG00000028017,ENSMUSG00000028012,ENSMUSG00000037994,ENSMUSG00000028195,ENSMUSG00000036832,ENSMUSG00000023267,ENSMUSG00000038668,ENSMUSG00000028655,ENSMUSG00000012123,ENSMUSG00000038276,ENSMUSG00000043635,ENSMUSG00000005057,ENSMUSG00000029862,ENSMUSG00000029797,ENSMUSG00000079495,ENSMUSG00000004328,ENSMUSG00000051965,ENSMUSG00000085601,ENSMUSG00000040714,ENSMUSG00000044786,ENSMUSG00000038763,ENSMUSG00000048782,ENSMUSG00000042828,ENSMUSG00000070369,ENSMUSG00000031004,ENSMUSG00000009248,ENSMUSG00000054717,ENSMUSG00000000791,ENSMUSG00000005470,ENSMUSG00000053399,ENSMUSG00000061039 |
| 5 | query_1 | TRUE | 0.027763827663523 | 6489 | 133 | 54 | 0.406015037593985 | 0.00832177531206657 | GO:1901564 | GO:BP | organonitrogen compound metabolic process | 27205 | 22563 | c("GO:0006807", "GO:0071704") | IDA ISO IBA IEA,IEA,ISS ISO,IEA,IMP ISS ISO IEA,ISO IBA IEA,ISO IEA,IDA IMP ISO IEA,ISO IEA,IPI ISO IBA IEA,ISS IEA,IBA IEA,ISO IEA,IBA IEA,IBA IEA,ISO IEA,IBA IEA,IMP ISS ISO IEA,IBA IEA,IDA IMP ISO IBA IEA,IMP ISO IBA,IEA,IDA IGI,ISS ISO IEA,IDA ISO,IDA ISS ISO IBA TAS IC IEA,IDA IMP ISO IBA TAS IEA,IDA ISO IBA TAS IEA,IMP ISS ISO IBA IEA,IBA,ISS ISO IBA IEA,IDA ISO IEA,IDA IGI ISO IEA,IBA,IMP ISO IEA,IEA,IDA ISO IEA,ISO IEA,IDA,IDA,IEA,ISS ISO IBA IEA,ISO IBA IEA,IDA IBA IEA,IDA IMP ISS ISO IEA,IBA IEA,IEA,IEA,IBA,IMP IBA,IMP,IBA,IEA,TAS | ENSMUSG00000019970,ENSMUSG00000037370,ENSMUSG00000020108,ENSMUSG00000000731,ENSMUSG00000034579,ENSMUSG00000020182,ENSMUSG00000043448,ENSMUSG00000020893,ENSMUSG00000021025,ENSMUSG00000044734,ENSMUSG00000033102,ENSMUSG00000021903,ENSMUSG00000022219,ENSMUSG00000071350,ENSMUSG00000022096,ENSMUSG00000022622,ENSMUSG00000022766,ENSMUSG00000024190,ENSMUSG00000024222,ENSMUSG00000023067,ENSMUSG00000002769,ENSMUSG00000044176,ENSMUSG00000024810,ENSMUSG00000036587,ENSMUSG00000050211,ENSMUSG00000032715,ENSMUSG00000017697,ENSMUSG00000028141,ENSMUSG00000028111,ENSMUSG00000074340,ENSMUSG00000032854,ENSMUSG00000028017,ENSMUSG00000028195,ENSMUSG00000073910,ENSMUSG00000028655,ENSMUSG00000028862,ENSMUSG00000043635,ENSMUSG00000029716,ENSMUSG00000089694,ENSMUSG00000079495,ENSMUSG00000033508,ENSMUSG00000030074,ENSMUSG00000051965,ENSMUSG00000040424,ENSMUSG00000044786,ENSMUSG00000045467,ENSMUSG00000038763,ENSMUSG00000044952,ENSMUSG00000030935,ENSMUSG00000042828,ENSMUSG00000000791,ENSMUSG00000041679,ENSMUSG00000053399,ENSMUSG00000064342 |
| 6 | query_1 | TRUE | 0.0104341634705472 | 16296 | 134 | 109 | 0.813432835820896 | 0.00668875797741777 | GO:0005488 | GO:MF | binding | 25177 | 1484 | GO:0003674 | IEA,IPI ISO IEA,IBA IEA,IPI ISO IEA,IEA,IDA IBA,IPI ISS ISO IBA IEA,IPI ISO IBA TAS,IDA IBA IEA,IEA,ISS ISO IBA IEA,ISO IEA,IDA IPI ISO IBA IEA,IDA IPI IMP ISO IBA IEA,IBA IEA,IEA,IPI ISO IBA IEA,ISO IBA IEA,IPI ISO IBA TAS IEA,ISO IBA,ISO IBA IEA,IPI,IEA,ISO IEA,IEA,IPI ISO IBA IEA,ISO IBA IEA,IPI ISS ISO IEA,IEA,IEA,IPI IBA,IDA IPI ISO,ISO IEA,IPI ISO IEA,IDA IPI ISO IEA,IEA,IDA IBA,IEA,IDA ISO IBA IEA,IPI ISO IEA,IDA ISO IBA IEA,IPI ISO IEA,IDA IPI ISS ISO IBA TAS IEA,IEA,IPI ISO IEA,IDA IPI ISO IBA IEA,IDA IPI IBA IEA,IPI ISO IBA IEA,IEA,IDA ISS ISO IEA,IEA,IDA IBA IEA,IDA IPI ISS ISO IBA IEA,IDA IMP ISO IEA,ISO IBA IEA,ISO,IDA ISO IBA IEA,IPI ISO,ISO IEA,ISO IBA IEA,IBA IEA,IDA IPI ISO IEA,IEA,ISO IEA,IDA IBA IEA,ISO,ISO IEA,IEA,IPI ISO IEA,IEA,IEA,IEA,IEA,IDA IPI ISO IBA IEA,IPI ISO IEA,IPI IEA,ISS ISO IEA,IEA,IEA,IPI ISO IBA IEA,IDA IPI IBA IEA,IPI IEA,IDA IPI IBA IEA,IEA,IDA IPI ISS ISO IEA,IBA IEA,IEA,IEA,ISO IEA,IEA,IDA IPI ISO IEA,ISO IBA IEA,IEA,IPI,IPI ISO IEA,IDA ISO IBA IEA,IBA,IDA IPI ISS ISO IBA IEA,ISS ISO IEA,IPI ISO IBA IEA,IDA IBA,ISS ISO IBA TAS IEA,IDA ISS ISO IBA TAS IEA,ISO IBA TAS IEA,IEA,IEA,IBA,ISS IEA,IEA | ENSMUSG00000052688,ENSMUSG00000026697,ENSMUSG00000047369,ENSMUSG00000019970,ENSMUSG00000037370,ENSMUSG00000020108,ENSMUSG00000000731,ENSMUSG00000000732,ENSMUSG00000025401,ENSMUSG00000034579,ENSMUSG00000020424,ENSMUSG00000020182,ENSMUSG00000020893,ENSMUSG00000000093,ENSMUSG00000034936,ENSMUSG00000078607,ENSMUSG00000021025,ENSMUSG00000058669,ENSMUSG00000044988,ENSMUSG00000033715,ENSMUSG00000047246,ENSMUSG00000044734,ENSMUSG00000021903,ENSMUSG00000022219,ENSMUSG00000071350,ENSMUSG00000022096,ENSMUSG00000042622,ENSMUSG00000022622,ENSMUSG00000047034,ENSMUSG00000022766,ENSMUSG00000116780,ENSMUSG00000024190,ENSMUSG00000024215,ENSMUSG00000024222,ENSMUSG00000023067,ENSMUSG00000024300,ENSMUSG00000013766,ENSMUSG00000043807,ENSMUSG00000002769,ENSMUSG00000011305,ENSMUSG00000039481,ENSMUSG00000024131,ENSMUSG00000024430,ENSMUSG00000044176,ENSMUSG00000045664,ENSMUSG00000024810,ENSMUSG00000049134,ENSMUSG00000026669,ENSMUSG00000026774,ENSMUSG00000015090,ENSMUSG00000079484,ENSMUSG00000050211,ENSMUSG00000032715,ENSMUSG00000017697,ENSMUSG00000027525,ENSMUSG00000015852,ENSMUSG00000027938,ENSMUSG00000028141,ENSMUSG00000028111,ENSMUSG00000048458,ENSMUSG00000074340,ENSMUSG00000028017,ENSMUSG00000019232,ENSMUSG00000037994,ENSMUSG00000028195,ENSMUSG00000036832,ENSMUSG00000023267,ENSMUSG00000073910,ENSMUSG00000038668,ENSMUSG00000028862,ENSMUSG00000012123,ENSMUSG00000008307,ENSMUSG00000043635,ENSMUSG00000005057,ENSMUSG00000029716,ENSMUSG00000029641,ENSMUSG00000029862,ENSMUSG00000029797,ENSMUSG00000042213,ENSMUSG00000004328,ENSMUSG00000051965,ENSMUSG00000085601,ENSMUSG00000040714,ENSMUSG00000040424,ENSMUSG00000044786,ENSMUSG00000045467,ENSMUSG00000038763,ENSMUSG00000044952,ENSMUSG00000048782,ENSMUSG00000030935,ENSMUSG00000042828,ENSMUSG00000070369,ENSMUSG00000031004,ENSMUSG00000025509,ENSMUSG00000025511,ENSMUSG00000009248,ENSMUSG00000089736,ENSMUSG00000054717 (hmgb2),ENSMUSG00000025591,ENSMUSG00000000791,ENSMUSG00000005470,ENSMUSG00000031762,ENSMUSG00000031765,ENSMUSG00000031780,ENSMUSG00000041679,ENSMUSG00000053399,ENSMUSG00000061039,ENSMUSG00000064342,ENSMUSG00000045802 (hsf3) |
| 7 | query_1 | TRUE | 0.0474015322113349 | 10491 | 134 | 78 | 0.582089552238806 | 0.00743494423791822 | GO:0005515 | GO:MF | protein binding | 25177 | 1496 | GO:0005488 | IPI ISO IEA,IBA IEA,IPI ISO,IEA,IDA IBA,IPI ISS ISO IBA IEA,IPI ISO IBA TAS,IDA IBA IEA,ISO IEA,ISO IEA,IPI ISO IBA IEA,IPI,IEA,IPI ISO IBA IEA,IPI ISO IBA TAS IEA,ISO IEA,IPI,ISO IEA,IEA,IPI ISO,IPI,IEA,IPI IBA,IDA IPI ISO,IPI ISO IEA,IDA IPI ISO IEA,IEA,IDA IBA,IEA,ISO IBA IEA,IPI ISO IEA,IDA ISO IBA IEA,IPI,IPI ISS ISO,IEA,IPI ISO IEA,IDA IPI ISO IBA IEA,IDA IPI IBA IEA,IPI ISO IEA,IEA,IDA IPI ISS ISO IBA IEA,ISO IBA IEA,ISO,IPI ISO,ISO IEA,ISO IBA IEA,IDA IPI ISO IEA,ISO IEA,IDA IBA IEA,ISO,ISO IEA,IPI ISO IEA,IDA IPI ISO IBA IEA,IPI ISO IEA,IPI,ISS ISO IEA,IEA,IEA,IPI IEA,IPI,IPI IEA,IDA IPI IBA IEA,IDA IPI ISS ISO IEA,IBA,IEA,ISO IEA,IDA IPI ISO IEA,IBA,IEA,IPI,IPI ISO IEA,IEA,IBA,IDA IPI ISO IEA,IPI ISO IBA IEA,IDA IBA,ISO IBA TAS IEA,IEA | ENSMUSG00000026697 (myoc),ENSMUSG00000047369 (dnah14 dyneina),ENSMUSG00000019970 (sgk1),ENSMUSG00000037370 (enpp1 insulin),ENSMUSG00000020108 (ddit4 mielinacja),ENSMUSG00000000731 (aire mielination neuropathy), ENSMUSG00000000732,ENSMUSG00000025401 (myo1a),ENSMUSG00000020424 (castor1 mtorc pathway), ENSMUSG00000020182 (ddc) ,ENSMUSG00000020893 (per1),ENSMUSG00000000093,ENSMUSG00000078607,ENSMUSG00000021025 (nfkbia),ENSMUSG00000044988 (ucn 3 kortykotropina),ENSMUSG00000047246,ENSMUSG00000044734 (serpinb1a) ,ENSMUSG00000022219,ENSMUSG00000071350 (setdb2),ENSMUSG00000022096 (hr),ENSMUSG00000022622 (acr),ENSMUSG00000047034 9ANKRD 33),ENSMUSG00000116780 (DYNLT2A3, DYNEINA LANCUCH LEKKI) ,ENSMUSG00000024190 (DUSP1),ENSMUSG00000024222 (FKBP5),ENSMUSG00000023067 (CDKN1A) ,ENSMUSG00000024300 (MYO1F),ENSMUSG00000013766 ( LY6G6E),ENSMUSG00000043807 (( LY6G5B)),ENSMUSG00000002769,ENSMUSG00000011305 (PLIN5),ENSMUSG00000039481 (NRTN, CZYNNIK TROFINCZY ),ENSMUSG00000024131 (SLC3A1),ENSMUSG00000024430 (CABYR),ENSMUSG00000044176 (SPIN10),ENSMUSG00000045664 (CDC42EP2 FORMATION OF FACTIN),ENSMUSG00000024810 (IL33 LINGAND IL 1),ENSMUSG00000049134 (NRAP NEBULINA),ENSMUSG00000026669,ENSMUSG00000026774 (YME1 FOLDING PROTEIN PROCESS),ENSMUSG00000032715 (TRIB3),ENSMUSG00000027525 (Phosphatase and actin regulator 3 is an enzyme that in humans is encoded by the PHACTR3),ENSMUSG00000015852 (FCRL) ,ENSMUSG00000028141 (OAZ3),ENSMUSG00000028111 (Ctsk cathepsin K),ENSMUSG00000048458,ENSMUSG00000028017 (egf),ENSMUSG00000037994 (SLC9B2),ENSMUSG00000028195 (ccn1),ENSMUSG00000036832 (lpar3 bol neuropathyczny),ENSMUSG00000023267,ENSMUSG00000038668 (lpar1),ENSMUSG00000005057(SH2B2 (also known as rAPS) decreased in DPN patients),ENSMUSG00000029716 (Transferrin receptor 2),ENSMUSG00000029641 RASL11A (RAS Like Family 11 Member A),ENSMUSG00000029862 ( Clcn1),ENSMUSG00000029797 (SCO-spondyna to białko, które u ludzi jest kodowane przez gen SSPO),ENSMUSG00000042213 (Zfand4),ENSMUSG00000004328 (hif3a),ENSMUSG00000051965 (nanos2),ENSMUSG00000085601 (meiosin),ENSMUSG00000040714 (klc3),ENSMUSG00000044786 (zfp36),ENSMUSG00000045467 (Ttll13),ENSMUSG00000044952 (KCTD21),ENSMUSG00000048782 (INSC),ENSMUSG00000042828 (trim72),ENSMUSG00000070369 (itgad integrin),ENSMUSG00000031004 (mki67),ENSMUSG00000025509 (Pnpla2),ENSMUSG00000025511 (tspan4),ENSMUSG00000009248 (Ascl2),ENSMUSG00000089736 (TGFBR3L),ENSMUSG00000054717 (hmgb2),ENSMUSG00000000791 (il12RB1),ENSMUSG00000005470 (Histone chaperone ASF1B is a protein that in humans is encoded by the ASF1B ),ENSMUSG00000031780 (Ccl17),ENSMUSG00000041679 (F-box and leucine-rich repeat protein 9 ) |
| 8 | query_1 | TRUE | 0.000127281658520633 | 4412 | 142 | 56 | 0.394366197183099 | 0.0126926563916591 | TF:M00257_1 | TF | Factor: RREB-1; motif: CCCCAAACMMCCCC; match class: 1 | 21866 | 2935 | TF:M00257 | TF,TF,TF,TF,TF,TF,TF,TF,TF,TF,TF,TF,TF,TF,TF,TF,TF,TF,TF,TF,TF,TF,TF,TF,TF,TF,TF,TF,TF,TF,TF,TF,TF,TF,TF,TF,TF,TF,TF,TF,TF,TF,TF,TF,TF,TF,TF,TF,TF,TF,TF,TF,TF,TF,TF,TF | ENSMUSG00000000732,ENSMUSG00000078607,ENSMUSG00000037336,ENSMUSG00000044734,ENSMUSG00000022219,ENSMUSG00000042622,ENSMUSG00000047034,ENSMUSG00000043747,ENSMUSG00000024190,ENSMUSG00000057246,ENSMUSG00000013766,ENSMUSG00000043807,ENSMUSG00000053835,ENSMUSG00000002769,ENSMUSG00000002831,ENSMUSG00000039481,ENSMUSG00000044176,ENSMUSG00000036587,ENSMUSG00000050211,ENSMUSG00000032715,ENSMUSG00000017697,ENSMUSG00000027525,ENSMUSG00000023393,ENSMUSG00000042784,ENSMUSG00000109293,ENSMUSG00000027938,ENSMUSG00000048458,ENSMUSG00000032854,ENSMUSG00000019232,ENSMUSG00000023267,ENSMUSG00000038668,ENSMUSG00000028784,ENSMUSG00000038276,ENSMUSG00000043635,ENSMUSG00000005057,ENSMUSG00000029716,ENSMUSG00000029797,ENSMUSG00000089694,ENSMUSG00000051262,ENSMUSG00000030074,ENSMUSG00000041377,ENSMUSG00000051811,ENSMUSG00000004328,ENSMUSG00000085601,ENSMUSG00000040424,ENSMUSG00000044952,ENSMUSG00000030935,ENSMUSG00000025509,ENSMUSG00000025511,ENSMUSG00000009248,ENSMUSG00000000791,ENSMUSG00000005470,ENSMUSG00000031765,ENSMUSG00000053399,ENSMUSG00000045802,ENSMUSG00000072969 |
| 9 | query_1 | TRUE | 0.00355462135460628 | 10750 | 142 | 97 | 0.683098591549296 | 0.00902325581395349 | TF:M00257 | TF | Factor: RREB-1; motif: CCCCAAACMMCCCC | 21866 | 2934 | TF:M00000 | TF,TF,TF,TF,TF,TF,TF,TF,TF,TF,TF,TF,TF,TF,TF,TF,TF,TF,TF,TF,TF,TF,TF,TF,TF,TF,TF,TF,TF,TF,TF,TF,TF,TF,TF,TF,TF,TF,TF,TF,TF,TF,TF,TF,TF,TF,TF,TF,TF,TF,TF,TF,TF,TF,TF,TF,TF,TF,TF,TF,TF,TF,TF,TF,TF,TF,TF,TF,TF,TF,TF,TF,TF,TF,TF,TF,TF,TF,TF,TF,TF,TF,TF,TF,TF,TF,TF,TF,TF,TF,TF,TF,TF,TF,TF,TF,TF | ENSMUSG00000052688,ENSMUSG00000045968,ENSMUSG00000026697,ENSMUSG00000037370,ENSMUSG00000020108,ENSMUSG00000000731,ENSMUSG00000000732,ENSMUSG00000020424,ENSMUSG00000043448,ENSMUSG00000020893,ENSMUSG00000000093,ENSMUSG00000078607,ENSMUSG00000037336,ENSMUSG00000021025,ENSMUSG00000058669,ENSMUSG00000044734,ENSMUSG00000118537,ENSMUSG00000022219,ENSMUSG00000042622,ENSMUSG00000047034,ENSMUSG00000043747,ENSMUSG00000024190,ENSMUSG00000024300,ENSMUSG00000057246,ENSMUSG00000013766,ENSMUSG00000043807,ENSMUSG00000053835,ENSMUSG00000002769,ENSMUSG00000011305,ENSMUSG00000002831,ENSMUSG00000039481,ENSMUSG00000024131,ENSMUSG00000024430,ENSMUSG00000044176,ENSMUSG00000045664,ENSMUSG00000049134,ENSMUSG00000036587,ENSMUSG00000015090,ENSMUSG00000079484,ENSMUSG00000050211,ENSMUSG00000032715,ENSMUSG00000017697,ENSMUSG00000027525,ENSMUSG00000023393,ENSMUSG00000015852,ENSMUSG00000042784,ENSMUSG00000109293,ENSMUSG00000027938,ENSMUSG00000028111,ENSMUSG00000027848,ENSMUSG00000048458,ENSMUSG00000032854,ENSMUSG00000028012,ENSMUSG00000019232,ENSMUSG00000028195,ENSMUSG00000036832,ENSMUSG00000023267,ENSMUSG00000038668,ENSMUSG00000028784,ENSMUSG00000028862,ENSMUSG00000043085,ENSMUSG00000038276,ENSMUSG00000043635,ENSMUSG00000005057,ENSMUSG00000029716,ENSMUSG00000029641,ENSMUSG00000071537,ENSMUSG00000029862,ENSMUSG00000029797,ENSMUSG00000089694,ENSMUSG00000079495,ENSMUSG00000051262,ENSMUSG00000033508,ENSMUSG00000030074,ENSMUSG00000042213,ENSMUSG00000041377,ENSMUSG00000051811,ENSMUSG00000004328,ENSMUSG00000085601,ENSMUSG00000040424,ENSMUSG00000044786,ENSMUSG00000045467,ENSMUSG00000044952,ENSMUSG00000030935,ENSMUSG00000031004,ENSMUSG00000025509,ENSMUSG00000025511,ENSMUSG00000009248,ENSMUSG00000089736,ENSMUSG00000054717,ENSMUSG00000000791,ENSMUSG00000005470,ENSMUSG00000031765,ENSMUSG00000031780,ENSMUSG00000053399,ENSMUSG00000045802,ENSMUSG00000072969 |
| 10 | query_1 | TRUE | 0.0369485729482635 | 14470 | 142 | 116 | 0.816901408450704 | 0.00801658604008293 | TF:M00378_1 | TF | Factor: Pax-4; motif: NNNNNYCACCCB; match class: 1 | 21866 | 2615 | TF:M00378 | TF,TF,TF,TF,TF,TF,TF,TF,TF,TF,TF,TF,TF,TF,TF,TF,TF,TF,TF,TF,TF,TF,TF,TF,TF,TF,TF,TF,TF,TF,TF,TF,TF,TF,TF,TF,TF,TF,TF,TF,TF,TF,TF,TF,TF,TF,TF,TF,TF,TF,TF,TF,TF,TF,TF,TF,TF,TF,TF,TF,TF,TF,TF,TF,TF,TF,TF,TF,TF,TF,TF,TF,TF,TF,TF,TF,TF,TF,TF,TF,TF,TF,TF,TF,TF,TF,TF,TF,TF,TF,TF,TF,TF,TF,TF,TF,TF,TF,TF,TF,TF,TF,TF,TF,TF,TF,TF,TF,TF,TF,TF,TF,TF,TF,TF,TF | ENSMUSG00000052688,ENSMUSG00000045968,ENSMUSG00000026697,ENSMUSG00000019970,ENSMUSG00000020108,ENSMUSG00000000731,ENSMUSG00000000732,ENSMUSG00000025401,ENSMUSG00000034579,ENSMUSG00000020424,ENSMUSG00000020182,ENSMUSG00000020286,ENSMUSG00000043448,ENSMUSG00000020893,ENSMUSG00000000093,ENSMUSG00000034936,ENSMUSG00000078607,ENSMUSG00000037336,ENSMUSG00000021025,ENSMUSG00000058669,ENSMUSG00000033715,ENSMUSG00000047246,ENSMUSG00000044734,ENSMUSG00000118537,ENSMUSG00000021903,ENSMUSG00000022219,ENSMUSG00000022096,ENSMUSG00000042622,ENSMUSG00000022622,ENSMUSG00000047034,ENSMUSG00000116780,ENSMUSG00000043747,ENSMUSG00000024190,ENSMUSG00000024215,ENSMUSG00000023067,ENSMUSG00000024300,ENSMUSG00000057246,ENSMUSG00000013766,ENSMUSG00000043807,ENSMUSG00000053835,ENSMUSG00000002769,ENSMUSG00000011305,ENSMUSG00000002831,ENSMUSG00000039481,ENSMUSG00000024131,ENSMUSG00000024430,ENSMUSG00000045664,ENSMUSG00000049134,ENSMUSG00000036587,ENSMUSG00000015090,ENSMUSG00000079484,ENSMUSG00000017697,ENSMUSG00000027525,ENSMUSG00000023393,ENSMUSG00000015852,ENSMUSG00000109293,ENSMUSG00000027938,ENSMUSG00000028141,ENSMUSG00000028111,ENSMUSG00000027848,ENSMUSG00000048458,ENSMUSG00000032854,ENSMUSG00000028017,ENSMUSG00000028012,ENSMUSG00000037994,ENSMUSG00000028195,ENSMUSG00000036832,ENSMUSG00000073910,ENSMUSG00000038668,ENSMUSG00000028655,ENSMUSG00000028784,ENSMUSG00000028862,ENSMUSG00000012123,ENSMUSG00000043085,ENSMUSG00000008307,ENSMUSG00000038276,ENSMUSG00000043635,ENSMUSG00000005057,ENSMUSG00000029641,ENSMUSG00000071537,ENSMUSG00000029862,ENSMUSG00000029797,ENSMUSG00000089694,ENSMUSG00000079495,ENSMUSG00000051262,ENSMUSG00000033508,ENSMUSG00000089997,ENSMUSG00000030074,ENSMUSG00000042213,ENSMUSG00000041377,ENSMUSG00000051811,ENSMUSG00000004328,ENSMUSG00000051965,ENSMUSG00000040714,ENSMUSG00000040424,ENSMUSG00000044786,ENSMUSG00000045467,ENSMUSG00000044952,ENSMUSG00000048782,ENSMUSG00000030935,ENSMUSG00000042828,ENSMUSG00000031004,ENSMUSG00000025509,ENSMUSG00000025511,ENSMUSG00000009248,ENSMUSG00000089736,ENSMUSG00000054717,ENSMUSG00000000791,ENSMUSG00000002910,ENSMUSG00000005470,ENSMUSG00000031762,ENSMUSG00000031780,ENSMUSG00000041679,ENSMUSG00000053399,ENSMUSG00000045802,ENSMUSG00000072969 |
