# Supplementary figures and images for: Novel insights into neuropathy: The impact of prolonged hyperglycemia on long non-coding RNA expression
Source: PLoS One. 2025 Oct 27;20(10):e0334245. doi: 10.1371/journal.pone.0334245 (PMC12558608; doi:10.1371/journal.pone.0334245)

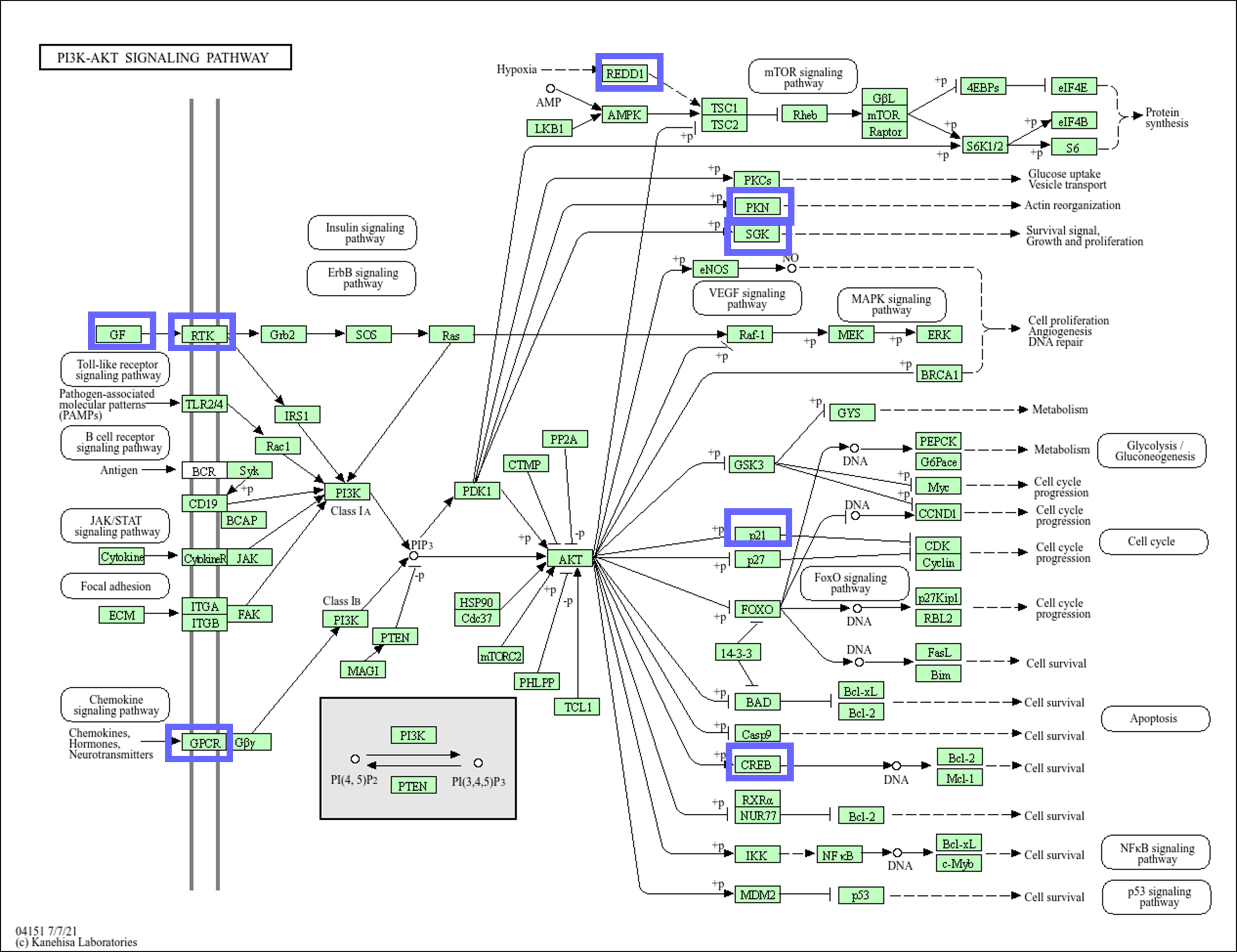

Supplement: S1 Fig — PI3K-Akt signaling pathway (KEGG: mmu04151). Blue rectangles without background indicate the site of lncRNA interaction. (TIF) [file pone.0334245.s004.tif]
