# Supplementary material for: Novel insights into neuropathy: The impact of prolonged hyperglycemia on long non-coding RNA expression
Source: PLoS One. 2025 Oct 27;20(10):e0334245. doi: 10.1371/journal.pone.0334245 (PMC12558608; doi:10.1371/journal.pone.0334245)
Supplement: Attachment — Submitted filename: Reviewer 3rev.docx [file pone.0334245.s006.docx]

Reviewer #3: The analysis of long non coding RNAs is not so deeply studied as other non conding RNAs, so a study focused on lncRNAs in neuropathy is interesting. Major points should be implemented:

Response: We would like to thank you for the very thorough reading of our manuscript. We are very excited by the positive critiques of our manuscript. Please find our point-by-point response below.

Reviewer #3:

- the model of diabetic neuropathy is not described, information is demanded to references 2 and 18. Ref 2 is a review, ref 18 does not seem related to diabetes model. Please clarify and clear detail the model used.

Response: Thank you for your suggestion. We have clarified this issue in the text of our revised MS (lines:58-64). We apologize for the inaccuracies.

Reviewer #3:

- Please report data regarding the development of diabetes and neuropathy in the animals used for transcriptomic analysis; at least at the time point considered for sacrifice (6 months)

Response: We have clarified the issue in the text of our revised MS (lines:64-66). We apologize for the inaccuracies.

Reviewer #3:

- Depending on the model, not all the animals with diabetes develop neuropathy, please report here the percentage. Were the non neuropathic animals excluded by the omic analysis?

Response: We have clarified this information in our revised MS (lines:64-66). We apologize for the inaccuracies. Briefly, we monitored blood glucose levels after five days after the last dose of STZ injection and for the following weeks to confirm diabetes. Nevertheless, we have many years of experience in inducing type 1 diabetes in a mouse model of the disease. Our experience has been supported by numerous scientific publications since 2013. Laboratorymouse is an excellent model for the induction of type 1 diabetes. Evidence supporting our thesis is provided by numerous publications using a mouse model in studies on the type 1 diabetes.

We have put a lot of effort into the presented analyses and studies in our MS. Moreover, mouse experiments were performed in accordance with the Local Ethical Committee of Experiments on Animals in Olsztyn (Poland; decision no. 57/2019) and the studies were reported in accordance with ARRIVE guidelines (https://arriveguidelines.org) and the three Rs principle. The 3 Rs stand for Replacement, Reduction and Refinement. Additionally, we report annually on the number of animals used in studies. We cannot exceed the number of animals used in study that is permitted by the Local Animal Ethics Committee. Hence, the number of animals that will not develop diabetes is less than 1%.

Reviewer #3:

- Snhg15 lncRNA expression was validated by RT-PCR. Why the attention was focused on this particular target? Since among several others one only was verified, the choice must be well supported.

Response: Our previous studies showed that the elevated expression of thioredoxin-interacting protein (TXNIP) in the T1D spinal cord of mouse (Zglejc-Waszak et al. 2023). Increased TXNIP expression in mouse spinal cord may induce endothelial cell dysfunction in the case of long-term hyperglycemia. Nevertheless, studies indicated molecular crosstalk between TXNIP and Snhg15 during diabetes. Overall, Snhg15 overexpression may improve endothelial function during diabetes and thus erase the negative effect of TXNIP in the spinal cord of mice. The analysis of Sngh15 was well-thought-out and results from the analysis of our previous research data.

In our previous work we validated data obtained from diabetic lumbar spinal cord samples. Our current results are a continuation of our previous studies (Zglejc-Waszak et al. 2023).

Reviewer #3:

- The discussion is mainly based on the description of Snhg15 and its relationship with PI3K (not measured here); the authors state "We assume that Snhg15 lncRNA may be responsible for malfunctions in nervous system during T1D", the authors should better analyze this hypothesis or strongly revise the discussion reducing the impact of only probabilistic suggestions.

Response: Thank you for your comment. We have slightly edited the Discussion section to soften its tone. However, our current results are a continuation of our previous studies (Zglejc-Waszak et al. 2023). Thus, we have well-established evidence that the spinal cord plays a key role in diabetic neuropathy in a mouse model of the disease. Identifying molecular changes occurring in the spinal cord during diabetes may be a milestone in inhibiting the development of peripheral neuropathy.
